# Insights into the κ-P,N Coordination of 1,3,5-Triaza-7-phosphaadamantane and Derivatives: κ-P,N-Heterometallic Complexes and a ^15^N Nuclear Magnetic Resonance Survey

**DOI:** 10.1021/acs.inorgchem.1c03831

**Published:** 2022-04-04

**Authors:** Andrés Alguacil, Franco Scalambra, Antonio Romerosa

**Affiliations:** Área de Química Inorgánica-CIESOL, Universidad de Almería, 04120 Almería, Spain

## Abstract

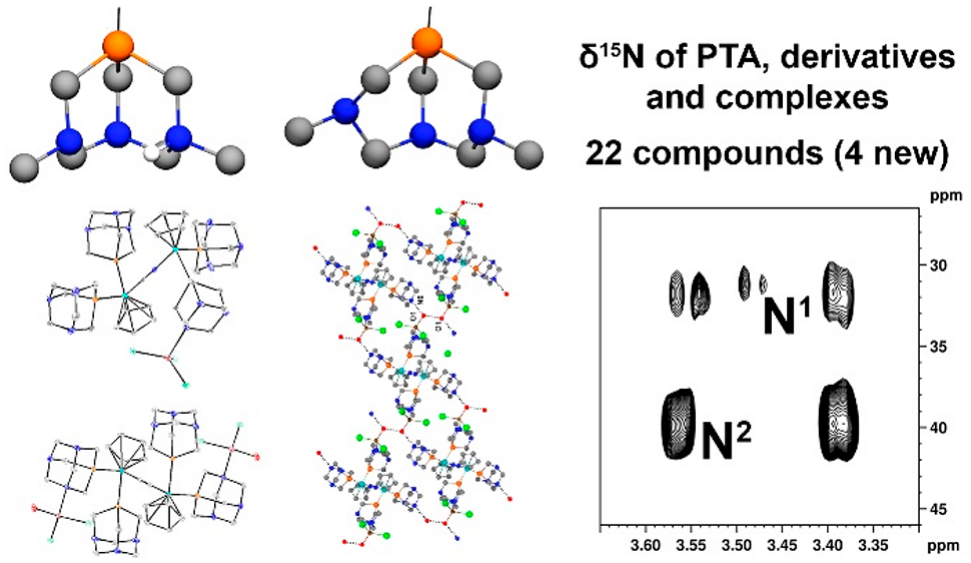

Complexes {[(PTA)_2_CpRu-μ-CN-1*κC*:2*κ*^2^*N*-RuCp(PTA)_2_-ZnCl_3_]}·2DMSO (**13**) {[ZnCl_2_(H_2_O)]-(PTA-1κ*P*:2κ^2^*N*)(PTA)CpRu-μ-CN-1κ*C*:2κ^2^*N*-RuCp(PTA)(PTA-1κ*P*:2κ^2^*N*)-[ZnCl_2_(H_2_O)]}Cl (**14**), [RuCp(HdmoPTA)(PPh_3_)(PTA)](CF_3_SO_3_)_2_ (**20**), [RuCp(HdmoPTA)(HPTA)(PPh_3_)](CF_3_SO_3_)_3_ (**21**), and [RuCp(dmoPTA)(PPh_3_)(PTA)](CF_3_SO_3_) (**22**) were obtained and characterized, and their crystal structure together with that of the previously published complex **18** is reported. The behavior of the 1,3,5-triaza-7-phosphatricyclo[3.3.1.13,7]decane (PTA) and 3,7-dimethyl-1,3,7-triaza-5-phosphabicyclo[3.3.1]nonane (dmoPTA) ligands against protonation and κ*N*-coordination is discussed, on the basis of ^15^N nuclear magnetic resonance data collected on 22 different compounds, including PTA (**1**), HdmoPTA (**7**H), and some common derivatives as free ligands (**2–6** and **8**), along with mono- and polymetallic complexes containing PTA and/or HdmoPTA (**9–22**). ^15^N detection via ^1^H–^15^N heteronuclear multiple bond correlation allowed the construction of a small library of ^15^N chemical shifts that shed light on important features regarding κ*N*-coordination in PTA and its derivatives.

## Abstract 2

To shed light on the behavior of the triazaphosphines 1,3,5-triaza-7-phosphatricyclo[3.3.1.13,7]decane (PTA) and 3,7-dimethyl-1,3,7-triaza-5-phosphabicyclo[3.3.1]nonane (dmoPTA) upon κN coordination and N protonation, the ^15^N chemical shifts of 22 compounds, including PTA and a representative variety of its derivatives, piano-stool complexes, were collected by ^1^H−^15^N heteronuclear multiple bond correlation nuclear magnetic resonance. New heterometallic complexes containing PTA were also synthesized and fully characterized.

## Introduction

**Figure 1:**
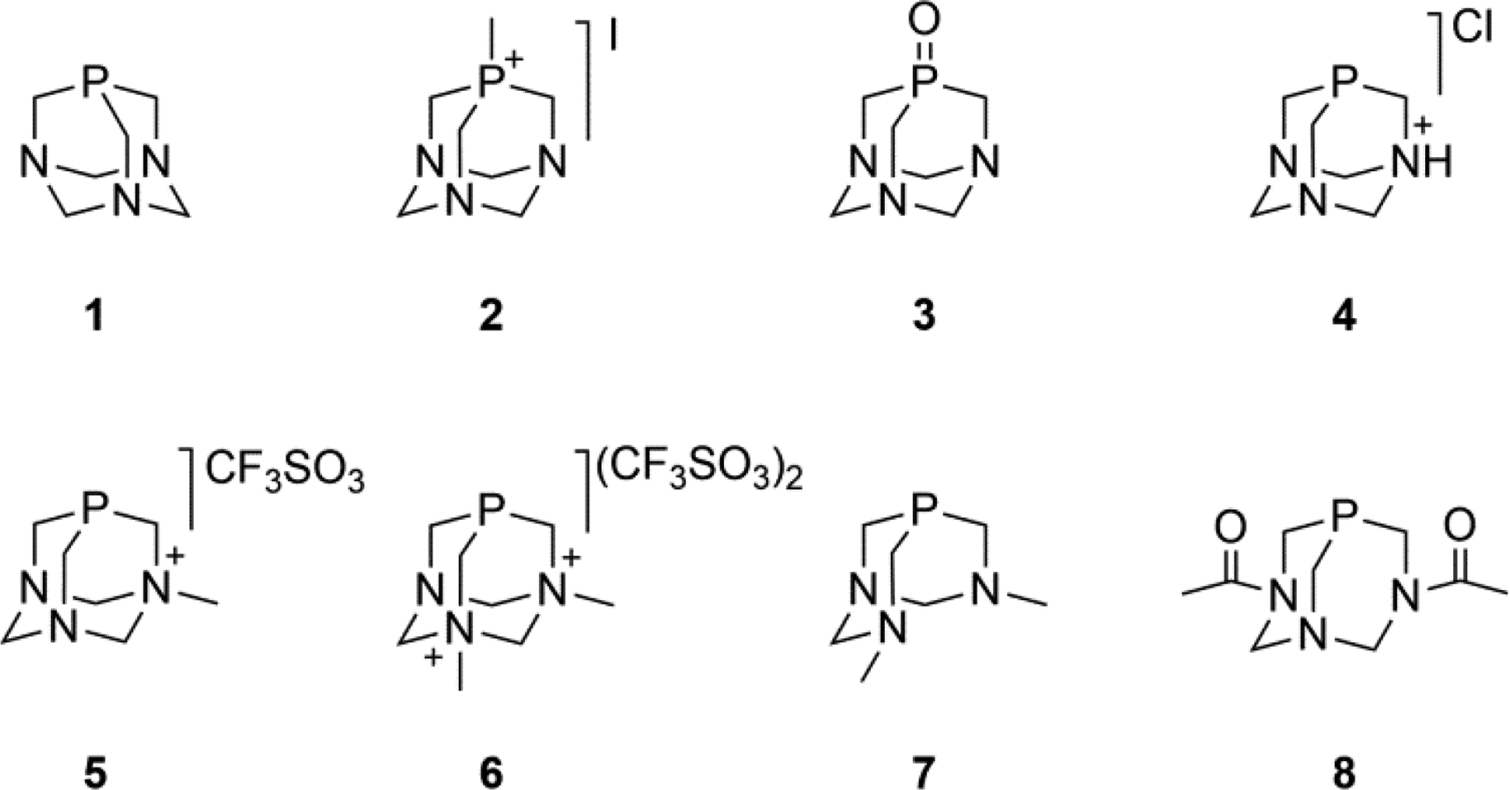
Structures of 1,3,5-triaza-7-phosphaadamantane (PTA) (**1**) and the derivatives studied in this work.

Today, hydrophilic phosphines are very common ligands in organometallic and coordination chemistry.^1,2^ In this class of compounds, monodentate *m*-monosulfonated PPh_3_ (*m*-TPPMS) and tris-*m*-sulfonated PPh_3_ (*m*-TPPTS) are among the most popular examples, but bidentate diphosphines and tridentate tripodal phosphines are also known and have been used.^3^ There are also examples of hydrosoluble cage-like phosphines such as Verkade-type phospha-amides^4^ and 1,3,5-triaza-7-phosphaadamantane (**1**), which was first reported in 1974 by Daigle et al. (usually abbreviated as PTA or pta; the acronym TPA and the name “monophosphaurotropine” have been also used to indicate the ligand; the IUPAC name is rarely used in the scientific literature).^5^ This ligand contains a soft phosphorus atom and three hard nitrogen atoms, which can be functionalized providing a large variety of derivatives (some examples are shown in Figure 1),^6^ useful for obtaining catalysts,^7−13^ bioactive agents,^14−25^ luminescent compounds,^26,27^ and new materials.^28−35^

**Figure 2:**
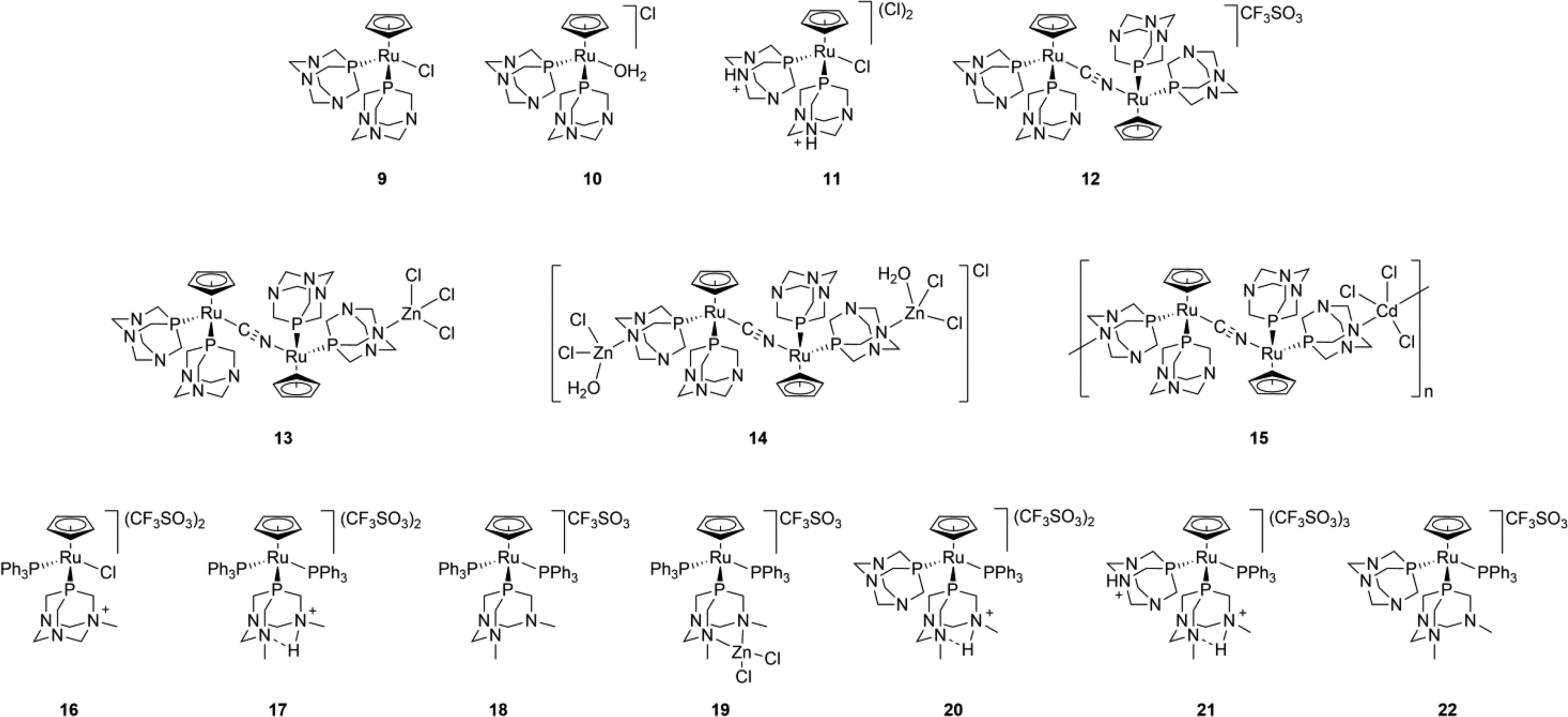
Structures of the complexes studied in this work.

During the past several years, we have devoted a great deal of effort to synthesizing mono- and polymetallic complexes containing PTA and its derivatives, affording good homogeneous catalysts for the isomerization of allylic alcohols in water^36−42^ like complex **9**,^43^ highly antiproliferative compounds^44−47^ such as compounds **17–19**,^48,49^ and heterometallic polymers like **15** built via assembly of dimetallic complex **12** through metallic moieties (Figure 2).^50^ The simplest functionalizations of the PTA cage are mono- and bis-N-methylation to afford the cationic ligands *N*-methyl-PTA (mPTA) (**5**) and *N*,*N′*-dimethyl-1,3,5-triaza-7-phosphaadamantane (dmPTA) (**6**).^46^ While the first is very stable, the latter decomposes under mild conditions, providing the ligand 3,7-dimethyl-1,3,7-triaza-5-phosphabicyclo[3.3.1]nonane (dmoPTA) (**7**). Half-sandwich Ru(II) complexes containing **7** and the protonated ligand 3,7-H-3,7-dimethyl-1,3,7-triaza-5-phosphabicyclo[3.3.1]nonane (HdmoPTA) (**7**H^+^), such as **17** and **18** (Figure 2), exhibit great antiproliferative activities^48^ and the ability to chelate a second metallic moiety through the methylated nitrogen atoms. It was shown that the chelation of a second metal, such as in the bimetallic complexes [RuCp(PPh_3_)_2_-μ-dmoPTA-1κ*P*:2κ^2^*N*,*N*′-ZnCl_2_](CF_3_SO_3_) (**19**) and [RuCp(PPh_3_)_2_-μ-dmoPTA-1κ*P*:2κ^2^*N*,*N*′-CoCl_2_](CF_3_SO_3_), improved the antiproliferative activity that was found to be 200 times higher than that of cisplatin for T-47D and WiDr human solid tumor cell lines.^47,49^

Most of the time, the coordination of PTA and its derivatives to one metal through the phosphorus atom can be proven by ^31^P nuclear magnetic resonance (NMR). Nevertheless, spectroscopic characterization of κ*N*-coordination by infrared (IR), Raman, ultraviolet–visible (UV–vis), and ^1^H and ^13^C NMR is not straightforward, and only single-crystal X-ray diffraction can provide the needed confirmation,^51−54^ which only ensures the N coordination only in the solid state. Thus, to obtain more information about whether and how PTA and its derivatives are coordinated by the N atoms, we thought to lean on nitrogen NMR.

The most abundant isotope of nitrogen, ^14^N, is quadrupolar; thus, most of the time, its detection is not practical. On the contrary, ^15^N has a spin of ^1^/_2_ but its natural abundance is only 0.365%; therefore, the duration for data collection for non-enriched samples is usually very long. Nevertheless, this issue can be bypassed by the detection of ^1^N via ^1^H through long-range correlation experiments, today run by using routine and robust pulse sequences as heteronuclear multiple-bond correlation (HMBC) or heteronuclear multiple quantum correlation.^55^ It is well established that ^1^H–^15^N HMBC is a very useful tool for the structural assignments in protein sequences^56^ and was also used for the characterization of natural products^57−61^ and the structural resolution of isomers.^62^ Also, various studies were published, including ^15^N-enriched coordination compounds such as bimetallic clusters,^63^ and in recent years, organometallic complexes were also characterized by this technique.^64−76^ In this work, we used the ^1^H–^15^N HMBC pulse sequence to investigate in solution some noncoordinated PTA derivatives (Figure 1) as well as some previously published representative Ru half-sandwich complexes displaying κ*P-* and κ*P,N*coordination (Figure 2). Additionally, the new monometallic (**20–22**) and polymetallic (**13** and **14**) complexes containing dmoPTA and/or PTA were synthesized and characterized by single-crystal X-ray diffraction, expanding the family of PTA-κ*P,N*-complexes, illuminating new aspects of the coordination behavior of the PTA and dmoPTA ligand in the solid state and solution.

## Experimental Procedures

### Synthesis and Characterization of **13** and **14**

**Scheme 1:**
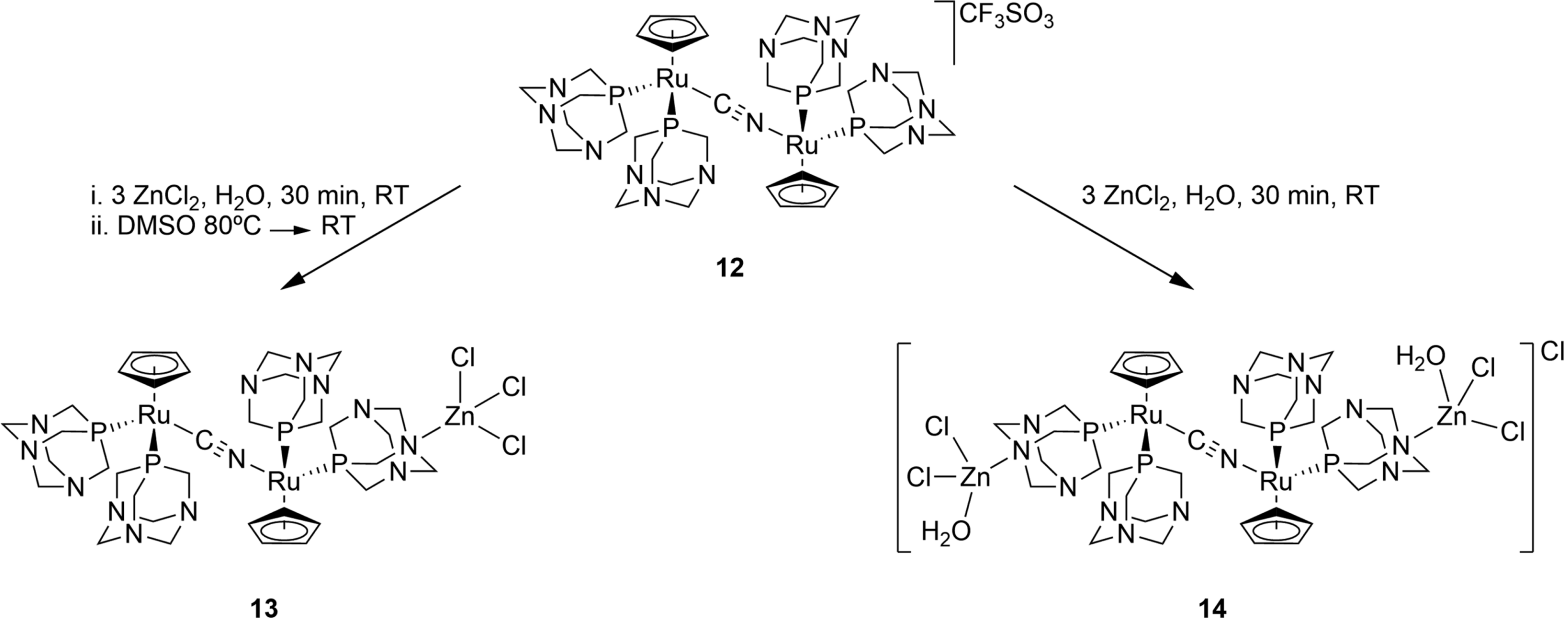
Synthesis of **13** and **14**

Trying to obtain an analogue of polymer **15** with Zn instead of Cd, we reacted bimetallic complex **12** with 3 equiv of ZnCl_2_ in water. Immediately, a light brown precipitated formed, which was redissolved in dimethyl sulfoxide (DMSO) and water. When the DMSO solution is cooled, complex [(PTA)_2_CpRu-μ-CN-1κ*C*:2κ^2^*N*-RuCp(PTA)_2_-ZnCl_3_] (**13**) crystallizes as the DMSO solvate, while upon evaporation of the water solution, tetrametallic complex {[ZnCl_2_(H_2_O)]-(PTA-1κ*P*:2κ^2^*N*)(PTA)CpRu-μ-CN-1κ*C*:2κ^2^*N*-RuCp(PTA)(PTA-1κ*P*:2κ^2^*N*)-[ZnCl_2_(H_2_O)]}Cl (**14**) was obtained (Scheme 1). Structures of these complexes were characterized by single-crystal X-ray diffraction, as described below, but the first assessment of their different composition was first allowed by comparison of their IR spectra. The cyanide vibration frequency is significantly different for **13** (2131 cm^–1^) and **14** (2109 cm^–1^), and the IR spectrum of **14** shows H_2_O stretching and bending bands (3479 and 1623 cm^–1^, respectively), which are absent in **13**.

The ^31^P{^1^H} NMR spectra in D_2_O of both complexes **13** and **14** display two singlets in a 1:1 ratio, at −19.8 and −22.4 ppm, respectively. This behavior agrees with the cleavage of the PTA-Zn bond upon dissolution, as previously reported for other PTA-k-*P,N* complexes.^50,77,78^

### Synthesis and Characterization of **20–22**

**Scheme 2:**
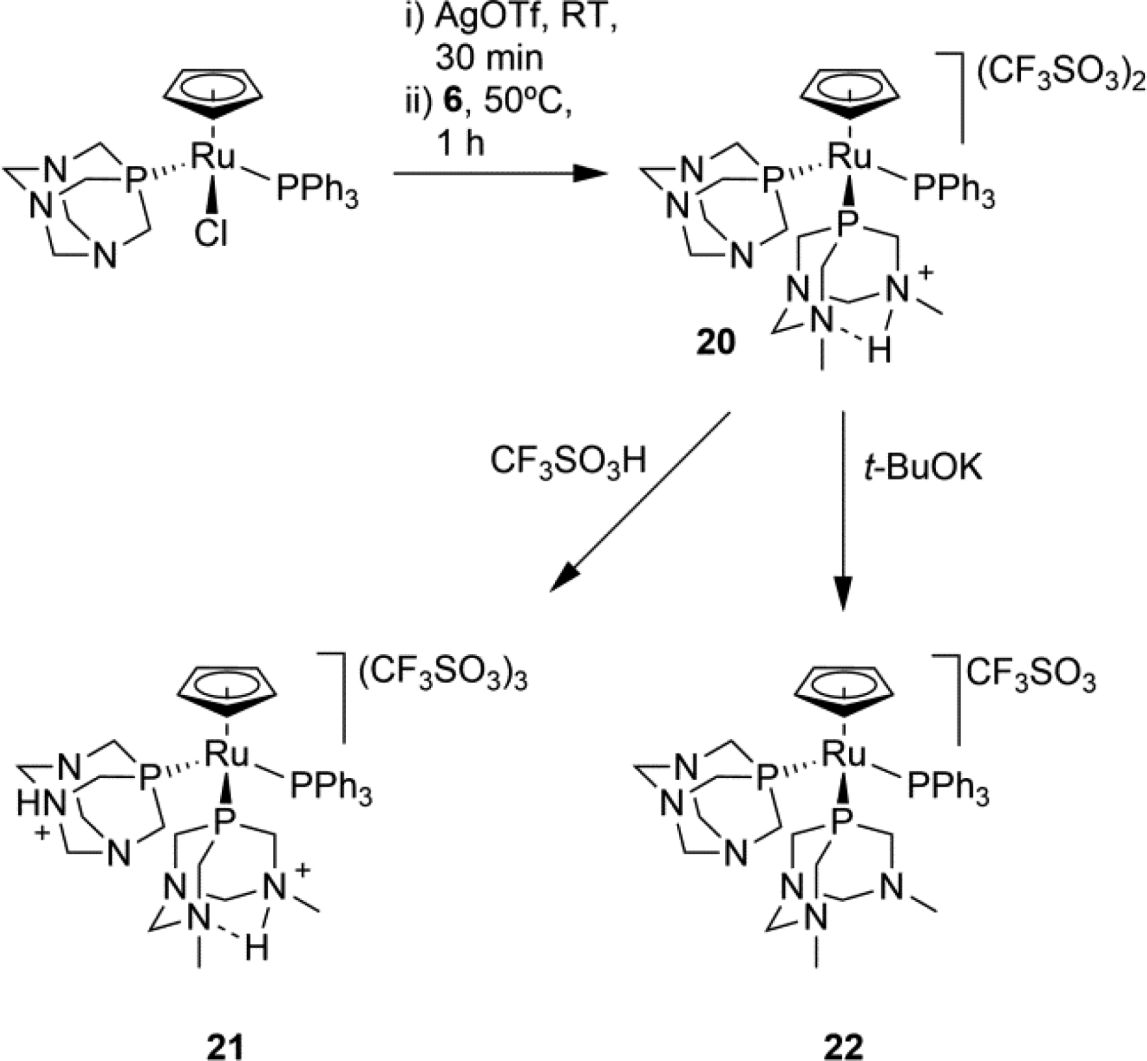
Synthesis of **20–22**

Complex [RuCp(HdmoPTA)(PPh_3_)(PTA)](CF_3_SO_3_)_2_ (**20**) was obtained in 81% yield by treatment of [RuClCp(PPh_3_)(PTA)] with AgOTf and further reaction with dmPTA (**6**) at 50 °C (Scheme 2). The presence of the CF_3_SO_3_^–^ anion in the complex composition was confirmed by both its ^13^C{^1^H} NMR and infrared spectra. Except for the resonances assigned to PTA, the ^1^H and ^13^C chemical shifts are similar to those of complex **17**, supporting that the ligand dmoPTA is protonated.^48^ Its ^31^P{^1^H} NMR spectrum in CD_3_OD displays the signals relative to PTA (−39.40 ppm), protonated dmoPTA (−3.99 ppm), and PPh_3_ (46.33 ppm) in a 1:1:1 ratio as an AMX pattern and agrees with those of previously reported compounds containing these ligands.^43,48^ Addition of 1.9 equiv of triflic acid to a solution of **20** in CD_3_OD shifts the phosphine signals to higher fields, arising at −27.09 ppm (HPTA^+^), −5.91 ppm (HdmoPTA^+^), and 44.81 ppm (PPh_3_). The large variation of the chemical shift for PTA (Δδ^31^P = 12.31 ppm) suggests its protonation to give complex [RuCp(HdmoPTA)(PPh_3_)(HPTA)](CF_3_SO_3_)_3_ (**21**). The ^1^H multiplets of the NC*H*_*2*_N atoms (4.82 ppm) are shifted to a higher field in **20** (Δδ^1^H = 0.32 ppm), as found after elucidating its ^1^H NMR spectrum by ^1^H COSY and ^1^H–^13^C HSQC NMR. The values observed for ^31^P, ^1^H, and ^13^C resonances agree with those of the similar previously reported complex. Finally, when **20** is reacted with 1.7 equiv of ^*t*^BuOK, deprotonated complex [RuCp(dmoPTA)(PPh_3_)(PTA)](CF_3_SO_3_) (**22**) forms. Its ^31^P{^1^H} NMR spectrum displays the signals of PTA, dmoPTA, and PPh_3_ at −37.54, 5.48, and 50.03 ppm, respectively, which are similar to what was observed for **18**. It is also worth mentioning that the carbons of the methyl groups are inequivalent, arising at 44.19 and 44.27 ppm in the ^13^C{^1^H} NMR spectrum, as evidenced by its ^1^H–^13^C HSQC spectrum.^48^

### Crystal Structure of **13** and **14**

**Figure 3:**
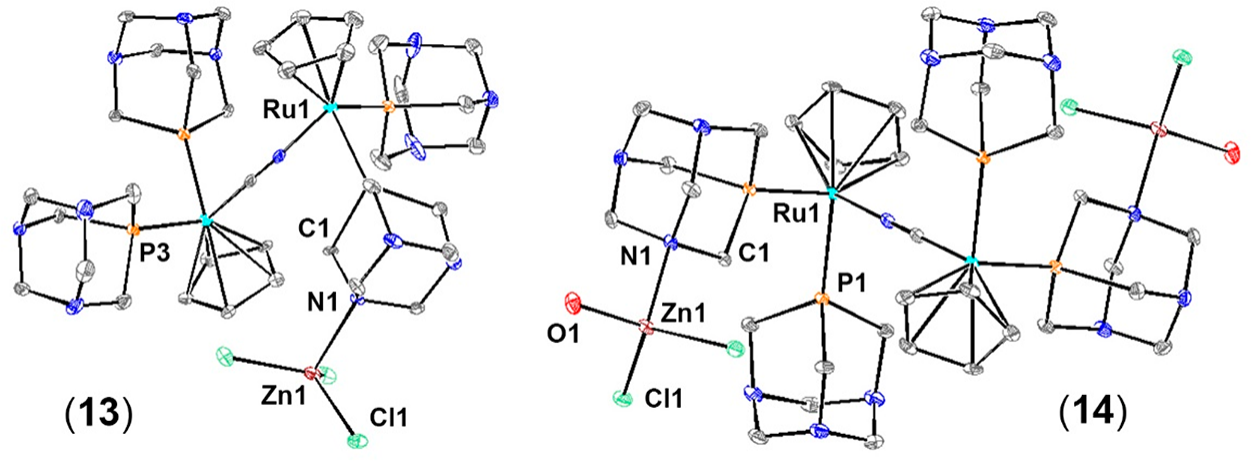
Thermal ellipsoid representation of the complex unit in the crystal structures of **13** and **14**. Their relevant bond lengths, plane angles, and torsion angles are listed in Tables S7, S8, S12, and S13. Anions, solvent molecules, and hydrogen atoms connected to the carbon atoms have been omitted for the sake of clarity.

Complexes **13** and **14** crystallize in the *P*_21_/*n* and *P*1̅ space groups, respectively. Selected bond lengths and angles are listed in Table 1, while a complete list can be found in Tables S7, S8, S12, and S13. In terms of **13**, the asymmetric unit contains one molecule of trimetallic complex [(PTA)_2_CpRu-μ-CN-1κ*C*:2κ^2^*N*-RuCp(PTA)-μ-PTA-1κ*P*:2κ^2^*N*-ZnCl_3_] (Figure 3) and two molecules of DMSO. The complex unit consists of two cyanide-bridged piano-stool {RuCp(PTA)_2_}^+^ moieties, which coordinate a {ZnCl_3_}^−^ anion through one nitrogen of a PTA ligand. In the case of **14**, the asymmetric unit contains a {ZnCl_2_(H_2_O)-μ-(PTA-1κ*P*:2κ^2^*N*)(PTA)CpRu-C*(N)*}^1/2+^ moiety (Figure 3) and ^1^/_2_Cl^–^, which upon growing around the inversion center affords a tetrametallic complex with a formula of {ZnCl_2_(H_2_O)-μ-(PTA-1κ*P*:2κ^2^*N*)(PTA)CpRu-μ-CN-1κ*C*:2κ^2^*N*-RuCp(PTA)-μ-(PTA-1κ*P*:2κ^2^*N*)-ZnCl_2_(H_2_O)}Cl. The bond lengths between the ruthenium and the phosphorus of the bidentate κ*P,N*-PTA ligands are almost identical in both complexes **13** and **14** [**13**, Ru1–P1 = 2.256(2) Å; **14**, Ru1–P1 = 2.2515(10) Å], while the same distances from monodentate κ*P*-PTA are longer in **13** and shorter in **14** [**13**, Ru1–P2 = 2.277(2) Å, Ru1–P3 = 2.264(2) Å, and Ru1–P4 = 2.272(2) Å; **14**, Ru1–P2 = 2.2477(11) Å]. The C≡N bond in **13** is slightly longer than that in **14** [**13**, CCNA–NCNA = 1.156(11) Å; **14**, CCN–NCN = 1.138(6) Å], which agrees with their C≡N vibration energies, as mentioned above, and the lengths are in the range found for complex **12**, polymer **15**, and its analogue polymers with Au, Co, and Ni, where the PTA acts as a bidentate κP,N ligand [**12**, 1.137(9) Å; **15**, 1.158(7) Å; *trans*-(**12**-CoCl_3_)_*n*_, 1.147(4) Å; *cis*-(**12**-CoCl_3_)*_n_*, 1.140(7) Å; *trans*-(**12**-NiCl_3_)*_n_*, 1.141(10) Å; *trans*-(**12**-Au(CN)_4_)_*n*_, 1.152(6) Å].^28,30,31,79^ In both complexes, Ru–C(*N*) distances are in the range found for similar compounds, as well as the bond lengths found for the PTA ligand.^35,80−83^

**Table 1:** Selected Bond Lengths and Angles for **13** and **14**

bond lengths (Å)						bond angles (deg)							
**13**			**14**			**13**				**14**			
Ru1	P1	2.256(2)	Ru1	P1	2.2515(10)	P1	Ru1	P2	95.60(8)	P1	Ru1	P2	96.89(4)
Ru1	P2	2.277(2)				P3	Ru2	P4	97.60(8)				
Ru2	P3	2.264(2)	Ru1	P2	2.2477(11)	N(*C*)CN	Ru1	P1	88.8(2)	NCN1	Ru1	P1	85.50(11)
Ru2	P4	2.272(2)				N(*C*)CN	Ru1	P2	86.5(2)				
Ru1	NCNA	2.021(8)	Ru1	N(*C*)CN	2.027(4)	C(*N*)CN	Ru2	P3	86.7(2)	NCN1	Ru1	P2	88.69(11)
Ru2	CCNA	2.023(8)				C(*N*)CN	Ru2	P4	89.0(2)				
NCNA	CCNA	1.156(11)	NCN	CCN	1.138(6)	Cl1	Zn1	Cl2	115.51(10)	Cl2	Zn1	Cl1	115.91(4)
Zn1	N1	2.123(7)	Zn1	N1	2.114(3)	Cl1	Zn1	Cl3	117.91(10)	N1	Zn1	Cl1	104.93(9)
Zn1	Cl1	2.235(3)	Zn1	O1	1.940(3)	Cl2	Zn1	Cl3	112.85(10)	N1	Zn1	Cl2	102.40(10)
Zn1	Cl2	2.246(2)	Zn1	Cl1	2.2455(12)	N1	Zn1	Cl1	99.34(19)	O1	Zn1	Cl1	110.90(10)
Zn1	Cl3	2.251(3)	Zn1	Cl2	2.2380(11)	N1	Zn1	Cl2	104.60(19)	O1	Zn1	Cl2	114.99(10)
						N1	Zn1	Cl3	103.8(2)	O1	Zn1	N1	106.32(13)

The zinc atoms in **13** and **14** display a distorted tetrahedral geometry and complete the coordination spheres with three chlorides in **13** and two chlorides and one H_2_O in **14**. The Zn–Cl bond lengths are comparable in both complexes (**13**, *d*_Zn–Cl_ = 2.244 Å; **14**, *d*_Zn–Cl_ = 2.242 Å), similar to the typical bond lengths in the structure of zinc chloride complexes in aqueous solution {[ZnCl_2_(PTA)_2_], *d*_Zn–Cl_ = 2.231 Å; [ZnCl_2_(μ-O=PTA)]_*n*_, *d*_Zn–Cl_ = 2.218 Å; [ZnCl_2_(O=PTA)(H_2_O)], *d*_Zn–Cl_ = 2.218 Å; [ZnCl_2_(S=PTAH)(S=PTAZnCl_3_)], *d*_Zn–Cl_ = 2.245 Å},^77,78,84^ while the Zn–N bond in **13** is slightly longer than that in **14** [**13**, Zn1–N1 = 2.123(7) Å; **14**, Zn1–N1 = 2.114(3) Å] as well as other examples of Zn–N_PTA_ bonds {[ZnCl_2_(PTA)_2_], Zn1–N1 = 2.055(3) Å; [ZnCl_2_(μ-O=PTA)]*_n_*, Zn1–N1 = 2.108(16) Å; [ZnCl_2_(O=PTA)(H_2_O)], Zn1–N1 = 2.093(10) Å; [ZnCl_2_(S=PTAH)(S=PTAZnCl_3_)], Zn1–N1 = 2.085(15) Å}.^77,78,85^

**Figure 4:**
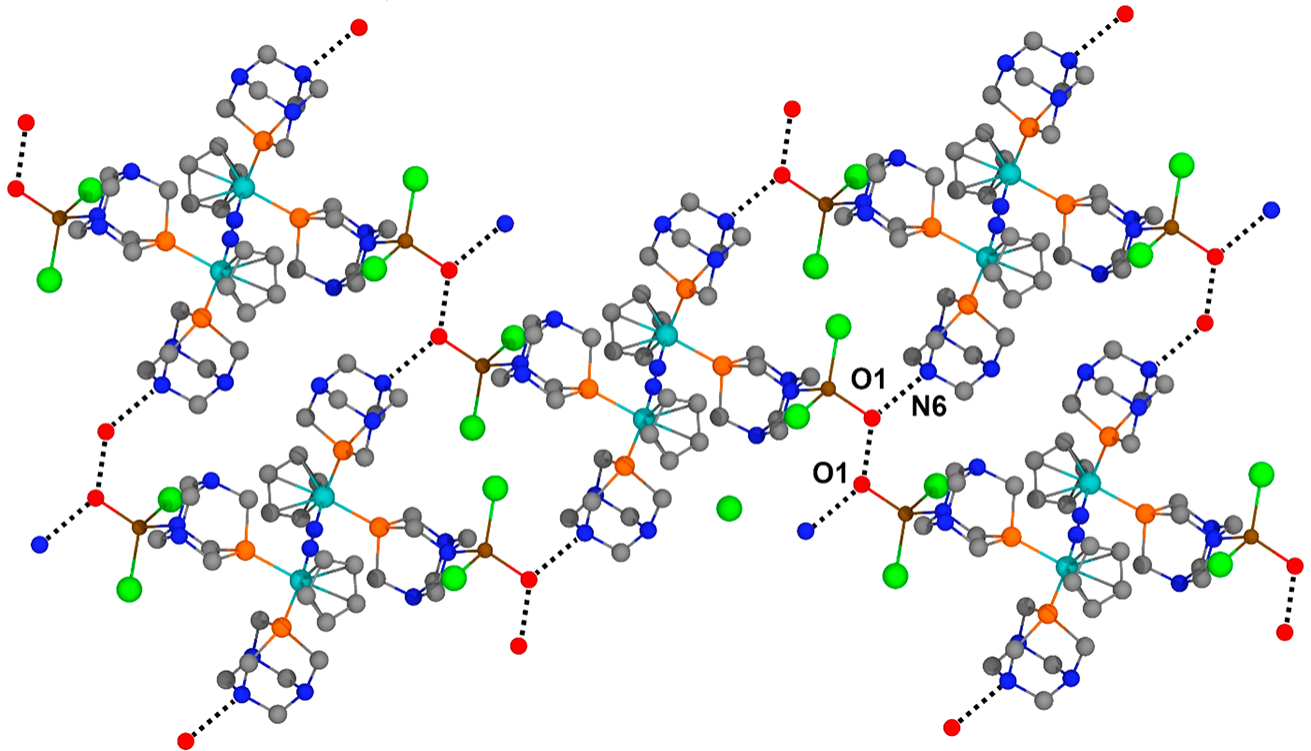
Hydrogen bonds around **14** forming a layered structure.

With regard to the intermolecular contacts, in the crystal packing of **13**, there is no interaction worth mentioning, while in **14**, each tetrametallic moiety of **14** is connected to four neighboring complexes via the O1 and N6 atoms, forming hydrogen-bonded layers along the reciprocal b*–c* plane (Figure 4).

### Crystal Structure of **18**, **21**, and **22**

**Figure 5:**
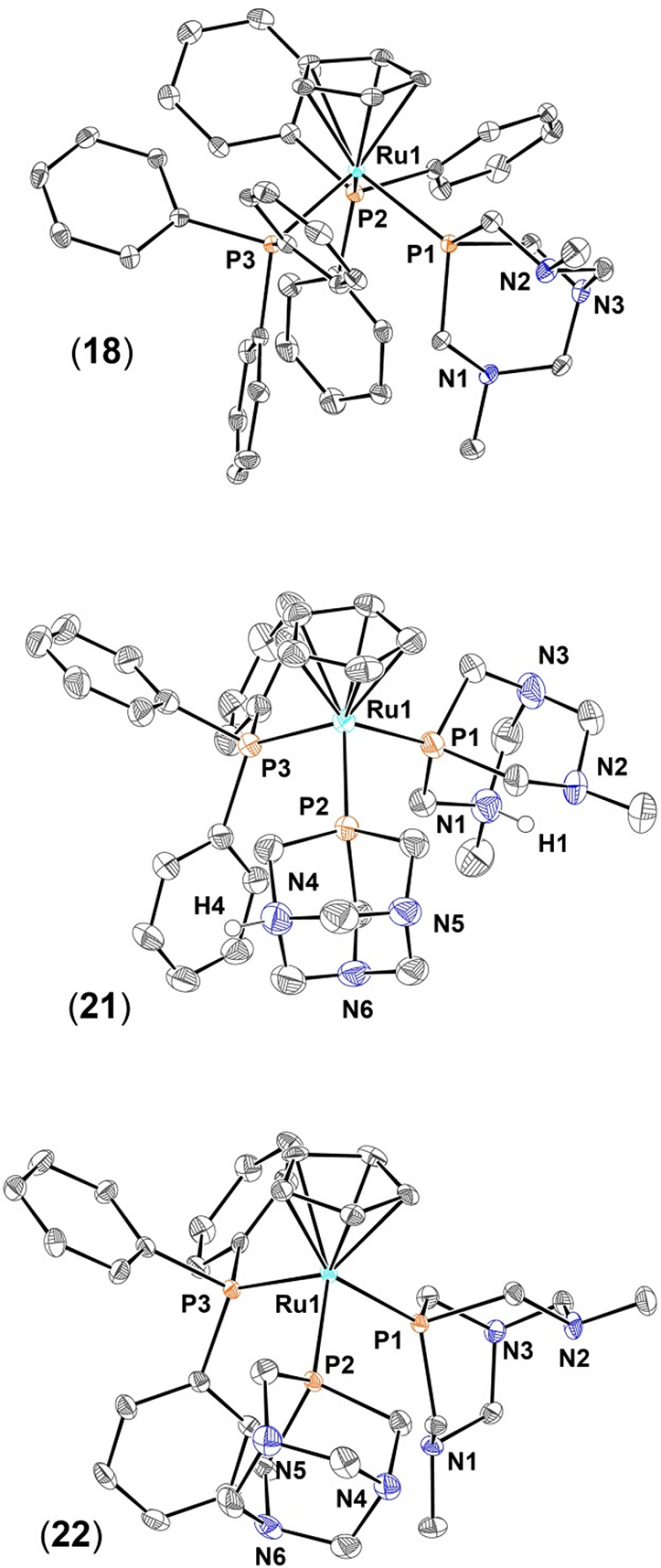
Thermal ellipsoid representations of the cationic portion of the crystal structures of **18**, **21**, and **22**. Their relevant bond lengths, plane angles, and torsion and angles are listed in Table 2, which also includes the data of **17**(48) for the sake of comparison (structure not shown). Anions, solvent molecules, and hydrogen atoms connected to carbons have been omitted for the sake of clarity.

Complex [RuCp(PPh_3_)_2_(dmoPTA-κ*P*)](CF_3_SO_3_)·CHCl_3_ (**18**) was previously prepared and published by us,^49^ but single crystals suitable for X-ray diffraction were obtained only recently by diffusion of Et_2_O vapor into a solution of the complex in chloroform, giving chloroform-solvated (**18**·CHCl_3_). Single crystals of complexes [RuCp(HdmoPTA)(PPh_3_)(HPTA)](CF_3_SO_3_)_3_·3H_2_O (**21**·H_2_O) and [RuCp(dmoPTA)(PPh_3_)(PTA)](CF_3_SO_3_)·MeOH (**22**·MeOH) were obtained by slow evaporation of a solution of the corresponding complex in water and methanol, respectively. Selected bond distances and angles for the three complexes are listed in Table 2, and complete lists of bond lengths and angles are provided in Tables S9–S11 and S14–S16. The asymmetric unit of complex **18**·CHCl_3_ contains a [RuCp(PPh_3_)_2_(dmoPTA-κ*P*)]^+^ molecule, one CF_3_SO_3_^–^, and one CHCl_3_. The coordination sphere of the ruthenium atom displays a piano-stool geometry and is constituted by a η^5^-Cp, two PPh_3_ ligands, and one dmoPTA ligand (Figure 5). Bond distances between the metal and the Cp centroid and phosphine P atoms are similar to those found for similar complexes (Table 2).^46,86−88^ The dmoPTA ligand is distorted (C1–P1–C2 = 102.15°; C1–P1–C3 = 95.86°; C2–P1–C3 = 94.84°), and the distance between the CH_3_*N* atoms [N1–N2 = 3.609(4) Å] is significantly larger than the distance for the equivalent protonated complex (2.80 Å). Comparison of the crystal structure of **18**·CHCl_3_ with its protonated form also shows that protonation of dmoPTA causes an extension of the three Ru–P bonds [Δ_*d*_(Ru1–P1) = +0.0023 Å, Δ_*d*_(Ru1–P2) = +0.0119 Å, and Δ_*d*_(Ru1–P3) = +0.0326 Å].^48^

**Table 2:** Selected Bond Lengths and Angles for **18**, **21**, and **22**^a^

		bond length (Å)			
atoms		**17**^b^	**18**	**21**	**22**
Ru1	P1	2.321(1)	2.3187(9)	2.2821(14)	2.3066(8)
Ru1	P2	2.366(1)	2.3541(8)	2.2838(13)	2.3009(8)
Ru1	P3	2.389(1)	2.3564(9)	2.3433(12)	2.3116(8)
N1	N2	2.800(6)	3.609(4)	2.670(8)	3.565(4)

			bond angle (deg)			
atoms			**17**^b^	**18**	**21**	**22**
P1	Ru1	P2	96.58(4)	95.44(3)	95.65(5)	95.95(3)
P1	Ru1	P3	97.53(4)	94.27(3)	97.09(5)	94.12(3)
P2	Ru1	P3	103.30(4)	105.94(3)	97.14(4)	98.64(3)

a For the sake of comparison, the values obtained for **17** were also added.

b From ref (48).

**Figure 6:**
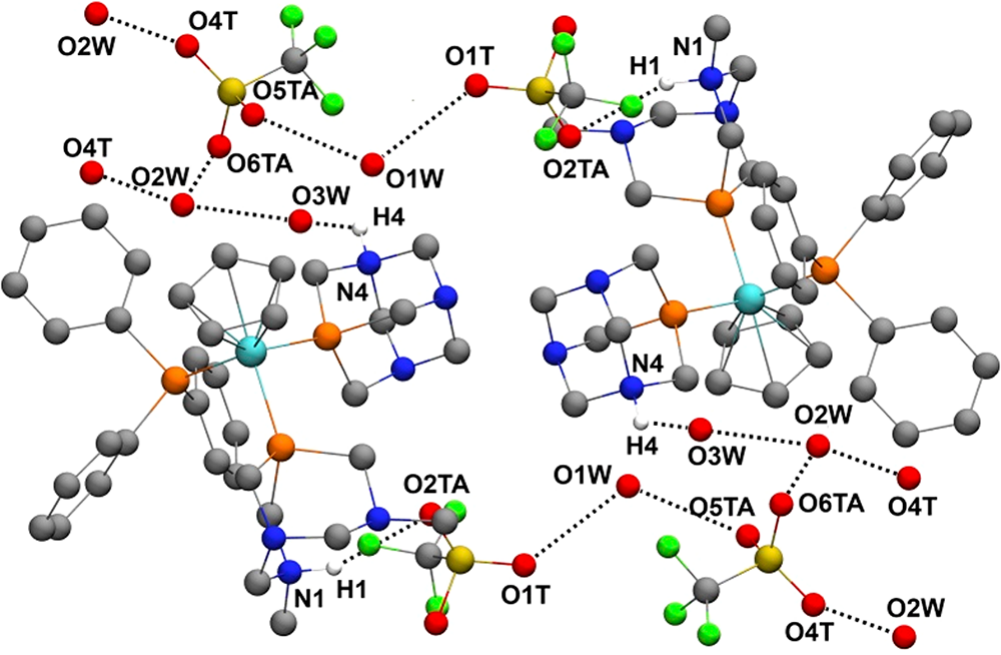
Hydrogen bonds network between adjacent molecules in the crystal structure of **21**.

Complexes **21** and **22** are constituted by the same complex moiety: a η^5^-Cp bonded to the Ru, which completes its coordination geometry by one κ*P*-PTA, one PPh_3_, and one κ*P*-dmoPTA. Nevertheless, in **21**, PTA and dmoPTA are monoprotonated, its composition being [RuCp(PPh_3_)(HPTA-κ*P*)(HdmoPTA-κ*P*)]^3+^, while in **22**, these ligands are deprotonated, its composition being [RuCp(PPh_3_)(PTA-κ*P*)(dmoPTA-κ*P*)]^+^ (Figure 5). In both **21** and **22**, the counterions are CF_3_SO_3_^–^, which are disordered in the lattice. The bond lengths and angles in complexes **21** and **22** are similar and fall in the ranges found for related complexes.^46−49,86,89^ Nevertheless, it is important to point out that the Ru–P_dmoPTA_ bond in **21** [Ru–P1 = 2.2821(14) Å] is significantly shorter than that in **22** [Ru–P1 = 2.3066(8) Å] and **18** [Ru–P1 = 2.3187(9) Å]. The same tendency is also observed for the Ru–PTA bond in **21** [Ru–P2 = 2.2838(13) Å], which is shorter than that in **22** [Ru–P2 = 2.3009(8) Å] and similar to the Ru–P_dmoPTA_ bond for this complex. Also, the Ru–PPh_3_ bond length is somewhat different in the three complexes [**18**, 2.3541(8) and 2.3564(9) Å; **21**, 2.3433(12) Å; **22**, 2.3116(8) Å]. The longest Ru–P bond observed in **18** is reasonable, due to the steric effect exerted by the PPh_3_ ligands, which also provoke a large distortion of the coordination geometry, as reflected by the angles between ligands [**18**, P1–Ru1–P2 = 95.44(3)°; P2–Ru1–P3 = 105.94(3)°; P1–Ru1–P3 = 94.27(3)°]. Nevertheless, compared to what found in dmoPTA–ZnCl_2_ complex **19**, the Ru–PPh_3_ bond length is only somewhat shorter in the protonated complex **21** and significantly shorter in deprotonated complex **22**. The cone angles^90−92^ of the HdmoPTA and dmoPTA ligands calculated from the crystal structure are only slightly different, ∼103° for **21** and ∼106° for **22**, leading to a similar angle between ligands [**21**, P1–Ru1–P3 = 97.09(5)°, P1–Ru1–P2 = 95.65(5)°, and P2–Ru1–P3 = 97.14(5)°; **22**, P1–Ru–P3 = 95.95(3)°, P2–Ru1–P1 = 94.12(3)°, and P2–Ru1–P3 = 98.64(3)°]. The distance between the CH_3_*N*_*dmoPTA*_ atoms is 2.670(8) Å for **21**, where the ligand is protonated, whereas for the deprotonated ligand in **22**, it is 3.565(4) Å, which is like that found in **18**. The protonation of PTA in **21** is localized on the N4 atom, which is part of a network of hydrogen bonds with water molecules and triflate anions (Figure 6).

**Figure 7:**
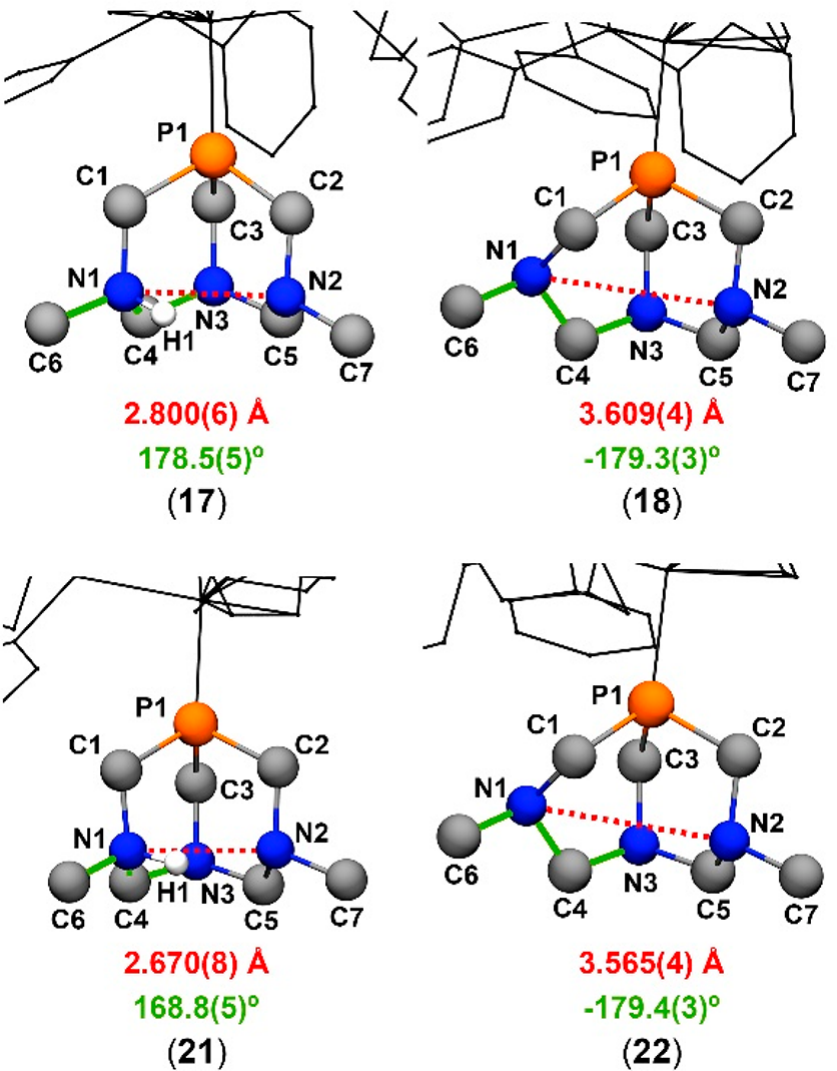
Highlights of ligands **7** and **7**H^+^ in **17**, **18**, **21**, and **22**. In red are shown distances between N1 and N2. In green are shown torsion angles among C6, N1, C4, and N3. For the sake of clarity, only hydrogen atoms bonded to nitrogen atoms are represented.

It is important to additionally consider the effect of the deprotonation of the HdmoPTA ligand in complexes **18** and **22**. When the CH_3_N_dmoPTA_ atoms are not H-bridged, the bottom rim of the triazaphosphaadamantane-like cage opens through an inversion about one of the methylated nitrogen atoms, which moves away from their lone pairs from each other. Given that in **18** and **22** the molecules are not connected by a network of hydrogen bonds, the greater N1–N2 length in **18** and **22** may suggest that their distance is susceptible to the steric hindrance of the surrounding ligands, which in **18** imposes a larger separation between the methyl groups (Figure 7). Intending to predict the N′–N″ chelation behavior of the dmoPTA ligand, we find these data shed light on the possibility of chelating also metals with a van der Waals radius larger than those of Zn, Ni, and Co, which are the only examples of dmoPTA-κ*N′,N″*-coordinated metals obtained to date.^89,93^

## Results and Discussion

### ^15^N Chemical Shifts of **1–22** via ^1^H–^15^N NMR Long-Range Correlations

#### δ^15^N of the Free Ligands

**Figure 8:**
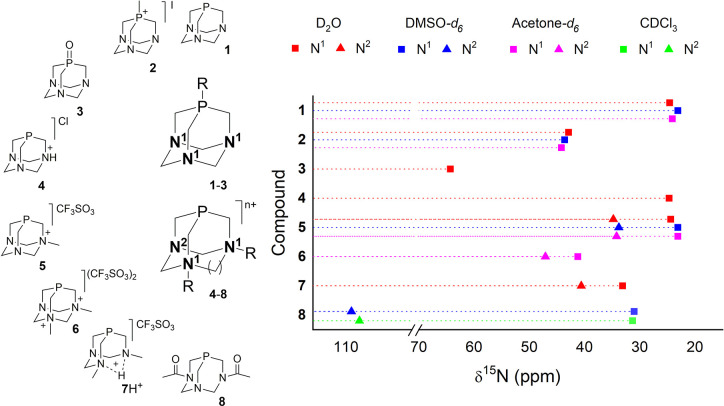
δ^15^N values for compounds **1–8**.

The adamantane-like phosphines and derivatives studied by ^1^H–^15^N long-range NMR correlations are displayed in Figure 1. Considering that selected metal-free triazaphosphines **1–3** display *C*_3*v*_ symmetry, while for ligands **4–8** is *C_S_*, in the first group only the cross peak due to three magnetically equivalent nitrogen atoms is expected, while for the second, two different signals should be found; the respective nitrogen atoms are numbered according to Figure 8. The obtained δ^15^N values for compounds **1–8** are summarized in Figure 8 and Table S1. As expected, only a ^15^N signal was observed for **1** in D_2_O that arises at 24.6 ppm, which is the expected region for a tertiary amine.^94^ When the phosphorus atom is functionalized, the ^15^N resonance of the PTA suffers inductive deshielding, as seen for **2** and **3**. Upon methylation of the phosphorus atom, compound **2** is obtained, which displays a singlet at 42.9 ppm, while oxidation of the phosphorus, which gives **3**, shifts the signal to 64.3 ppm.

Simple protonation of **1** affords compound **4** that displays a singlet due to fast proton exchange. This peak is shifted by only 0.1 ppm in D_2_O with respect to **1**, which is the usual behavior for sp^3^ nitrogens.^95^ Nevertheless, this fact indicates the large difference in the chemical shift between the phosphorus in ^31^P NMR, which is approximately 1 order of magnitude larger.^96^ Methylation of one of the nitrogen atoms of **1** affords the ligand mPTA (**5**), which displays the δ^15^N signal corresponding to quaternary nitrogen N^1^ that moved by only 0.2 ppm to a higher field with respect to **1**. On the contrary, the tertiary nitrogens N^2^ suffer a dramatic inductive effect, arising at 34.8 ppm in D_2_O. Therefore, the nonsubstituted PTA nitrogen atoms in **5** are shifted by almost +10 ppm with respect to **1** but −9 ppm with respect to P-methylated regioisomer **2**. These results show that the functionalization of the PTA at the phosphorus atom leads to a Δδ^15^N much more pronounced than that at the nitrogen. Further N methylation of **5** gives rise to N,N′-dimethylated derivative **6**, usually known as dmPTA, whose CH_3_N^1^- and N^2^-δ^15^N are found at 41.2 and 47.1 ppm, respectively, in acetone-*d*_6_, showing how these atoms are markedly deshielded by the second methylation. The Δδ^15^N^1^ and Δδ^15^N^2^ between **6** and **5** are larger (Δδ^15^N^1^ = +18.1 ppm, and Δδ^15^N^2^ = +12.9 ppm) than those between **5** and **1**, due to the inductive effect produced by the quaternary nitrogen atoms, which is doubled in **6** and reciprocally exercised by both N^1^. It is interesting to point out that the mono- and dimethylation of **1** produce a similar effect on the δ^31^P chemical shift.

Derivatives obtained by functionalization of PTA at the two nitrogen atoms suffer, under the appropriate conditions, the lysis of the CH_2_ group bridging the functionalized nitrogens.^97^ The simplest example may be compound **6**, which loses the methylene between the CH_3_*N* atoms, giving the neutral compound **7**, where the δ^15^N^1^ and δ^15^N^2^ in D_2_O are found at a higher field concerning **6** and also **1** (δ^15^N^1^ = 33.1 ppm; δ^15^N^2^ 40.6 ppm). The difference of ∼10 ppm between the signals of **1** and **7** could be caused by the opening of the adamantane-like cage. A similar shielding effect on the nonfunctionalized atom N^2^ is observed also for ligand **8**, usually known as DAPTA. For **8**, the δ^15^N^2^ arises at 31 ppm in DMSO-*d*_6_, while the acylated nitrogen atoms are found at 107.9–109.1 ppm, in the expected region for amides.^98^

It is important to evidence that the solvent polarity also has a slight influence on the chemical shift of the N atoms of the studied compounds. In terms of **2**, the ^15^N signal is linearly shielded with a decrease in solvent polarity, while compound **1** displays a different behavior: it suffers shielding when the more polar DMSO-*d*_6_ rather than acetone-*d*_6_ is used, but in D_2_O, the signal is deshielded by 0.5 ppm concerning acetone-*d*_6_. This behavior, which is shown also by **5**, can be tentatively addressed considering the possible involvement of the phosphorus atom in hydrogen bonding, which could induce the deshielding of the N_PTA_ atoms in a manner similar to but less intense than that caused by the methylation of the P_PTA_ atom.

### δ^15^N of Metal Complexes Containing Ligands **1**, **4**, and **5**

**Figure 9:**
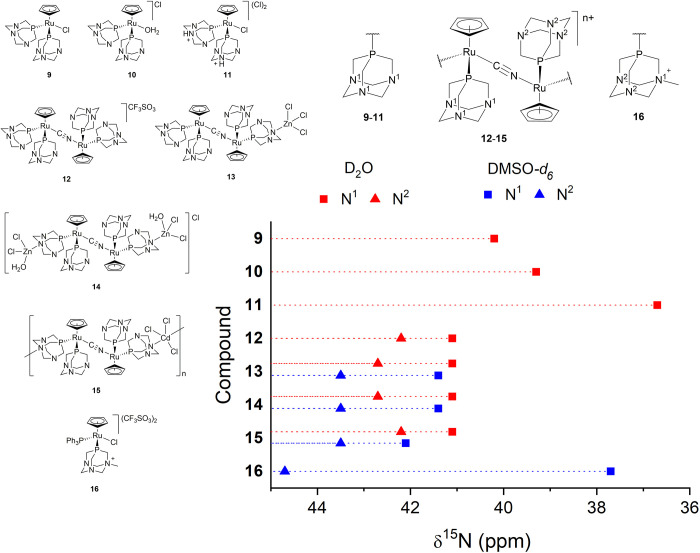
δ^15^N values for compounds **9–16**.

The ^1^H–^15^N HMBC of complex [RuClCp(PTA)_2_] (**9**), which contains two equivalent PTA ligands, is characterized by a correlation at 40.2 ppm in D_2_O that is relative to its six equivalent nitrogen atoms, together with another set of cross-peaks at 39.3 ppm. This additional signal can be assigned to complex [RuCp(PTA)_2_(D_2_O)]^+^ (**10**) that is in equilibrium with **9** in water.^99^ The δ^15^N^1^ of **11** was determined to be shifted by 3.5 ppm to **9** (Figure 9). As observed in **4**, only one ^15^N resonance is observed, due to fast proton exchange.

A new step in complexity is represented by diruthenium complex **12**, which was synthesized by the reaction of **9** with a half-equivalent of KCN.^79^ The ^15^N resonances for **12** arise in the same chemical shift range as **9**. Additionally, two singlets are observed at 40.8 and 41.8 ppm corresponding with N^1^ and N^2^ atoms, respectively (see Figure 9), which are due to the asymmetry of the cyanide bridge making the {RuCp(PTA)_2_}^+^ moieties inequivalent. N^1^ corresponds to the nitrogen atoms of the PTA bonded to the Ru-CN fragment, and N^2^ to those of the PTA bonded to the Ru-NC.

The coordination of one {ZnCl_3_}^−^ moiety or two {ZnCl_2_(H_2_O)} moieties to the nitrogen atoms of **12** leads to trimetallic complex **13** and tetrametallic complex **14**, respectively. The absence of multiplicity in their ^31^P{^1^H} NMR resonances suggests that the N–Zn bond is cleaved upon dissolution. This assumption can be also supported by their identical δ^15^N values (41.1 and 42.7 ppm in D_2_O and 41.4 and 43.5 ppm in DMSO-*d*_6_), very similar to those of **12**.

Under adequate reaction conditions, polymeric complexes such as **15** can be obtained from **12**, in which two PTA-N atoms are coordinated to two different {CdCl_3_}^−^ units. The ^1^H–^15^N HMBC of this polymer provides identical correlations with respect to **12–14**, supporting previous evidence that indicates that upon dissolution in water the Cd–N bonds are cleaved.^50^

Finally, complex **16**, which contains the methylated ligand mPTA (**5**), shows the resonances relative to methylated atom N^1^ and nonmethylated N^2^ in DMSO-*d*_6_, arising at 37.7 and 44.7 ppm, respectively. The differences in the chemical shift between the coordinated and free ligand (Δδ^15^N^1^ = +14.6 ppm, and Δδ^15^N^2^ = +10.9 ppm) are in the range found for the complexes containing **1**. Also, it is interesting to point out that N^1^ resonates at a frequency similar to that of the protonated species **11**.

In general, the ^15^N resonances for complexes **9–16** (Table S2) and compound **2** appear in a very narrow chemical shift range, suggesting that P methylation of the PTA and the κ*P*-coordination to the ruthenium produce a similar deshielding effect.

### δ^15^N of Metal Complexes Containing Ligand dmoPTA (**7**)

**Figure 10:**
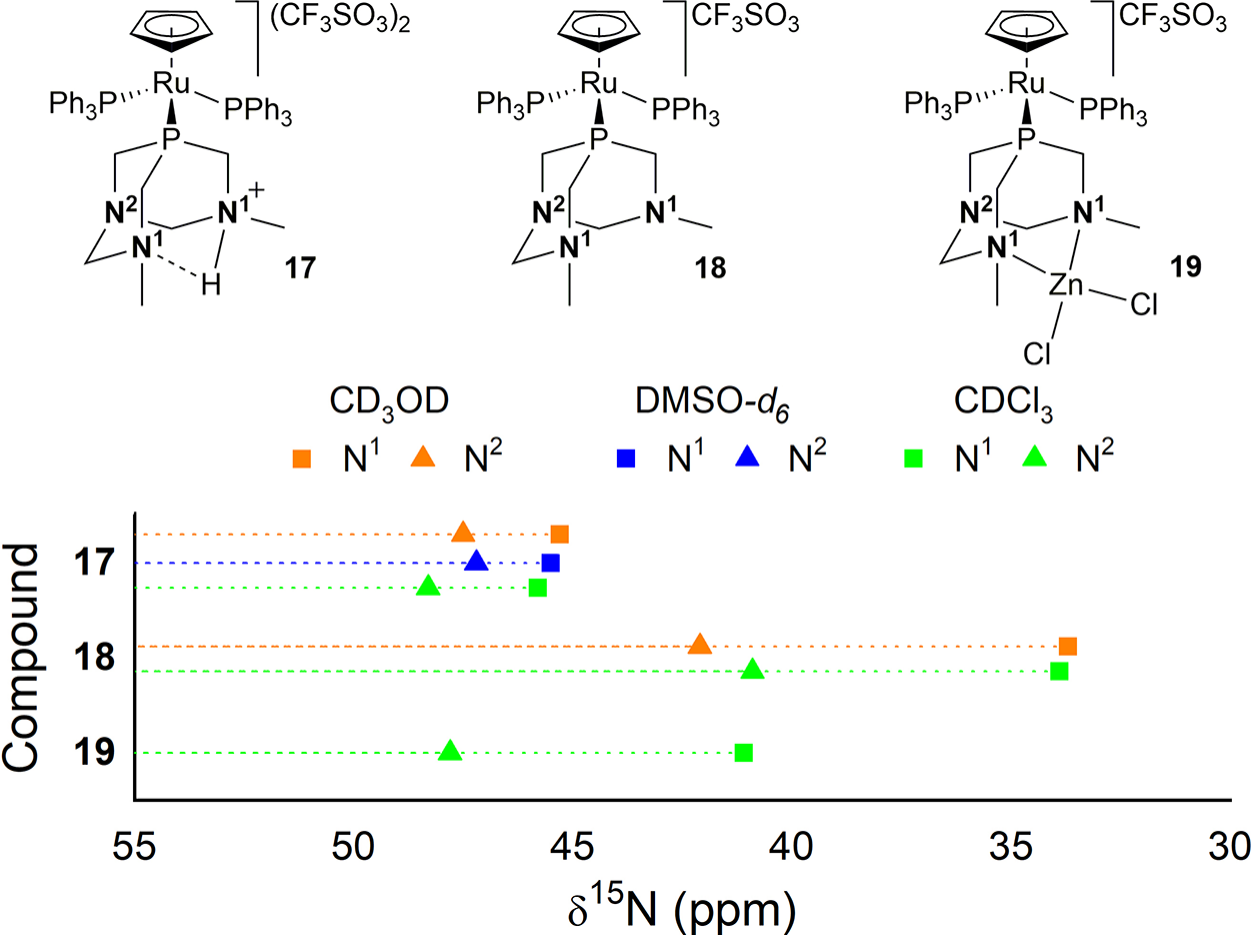
δ^15^N values for compounds **17–19**.

Generally, to avoid possible side reactions caused by the chelating nitrogen atoms, the complexes containing dmoPTA (**7**) are obtained employing **6** as a proligand. Under reaction conditions, once **6** is κ*P*-coordinated, it usually undergoes solvolysis of the CH_2_ bridging the ammonium groups and converts into phosphine **7** in its protonated form, **7**H^+^, which occurs when [RuCp(HdmoPTA)(PPh_3_)_2_](CF_3_SO_3_)_2_ (**17**) is synthesized. The ^1^H–^15^N HMBC spectrum of **17** displays two correlations at 45.3 and 47.5 ppm (CD_3_OD), which are slightly susceptible to solvent changes (Figure 10). The presence of just two sets of correlations in the ^1^H–^15^N HMBC spectrum supports the idea that the proton is shared between the two methylated nitrogen atoms N^1^. The deprotonation of **17** affords **18** and displaces the N^1^ chemical shift to 33.7 ppm in CD_3_OD (Table S3). Also, N^2^ is slightly shielded and resonates at 42.1 ppm. Surprisingly, after coordination to the Ru center, the δ^15^N values of **7** do not vary as much as observed for ligands **1** and **5**.

Ligand **7** can coordinate a variety of metallic centers through its methylated nitrogen atoms, affording bimetallic complexes whose antiproliferative activity is usually much higher than those of the monometallic parent compounds and cisplatin.^19,47−49,100^ This is the case for complex [RuCp(PPh_3_)_2_-μ-dmoPTA-1κ*P*:2κ^2^*N*,*N*′-ZnCl_2_](CF_3_SO_3_) (**19**), which is 5 times more potent than **18** and 425 times more potent than cisplatin on WiDr colon cancer cells. The chelation of the {ZnCl_2_} moiety closes the bottom rim of the dmoPTA ligand and deshields both N^1^ and N^2^, which in CDCl_3_ appear at 41.1 and 47.8 ppm, respectively, near the observed signals for **6** in acetone-*d*_6_. The trend shown by the δ^15^N values of compounds **17–19** is revealed to be very significant to assess the coordination of a second metallic unit to CH_3_N_dmoPTA_. The Δδ^31^P of the singlets found for **17** and **19** is only 1.2 ppm, while the δ^15^N of their methylated nitrogen differs by 4.21 ppm, making it easier to distinguish whether dmoPTA is protonated or coordinated to a metal.

**Figure 11:**
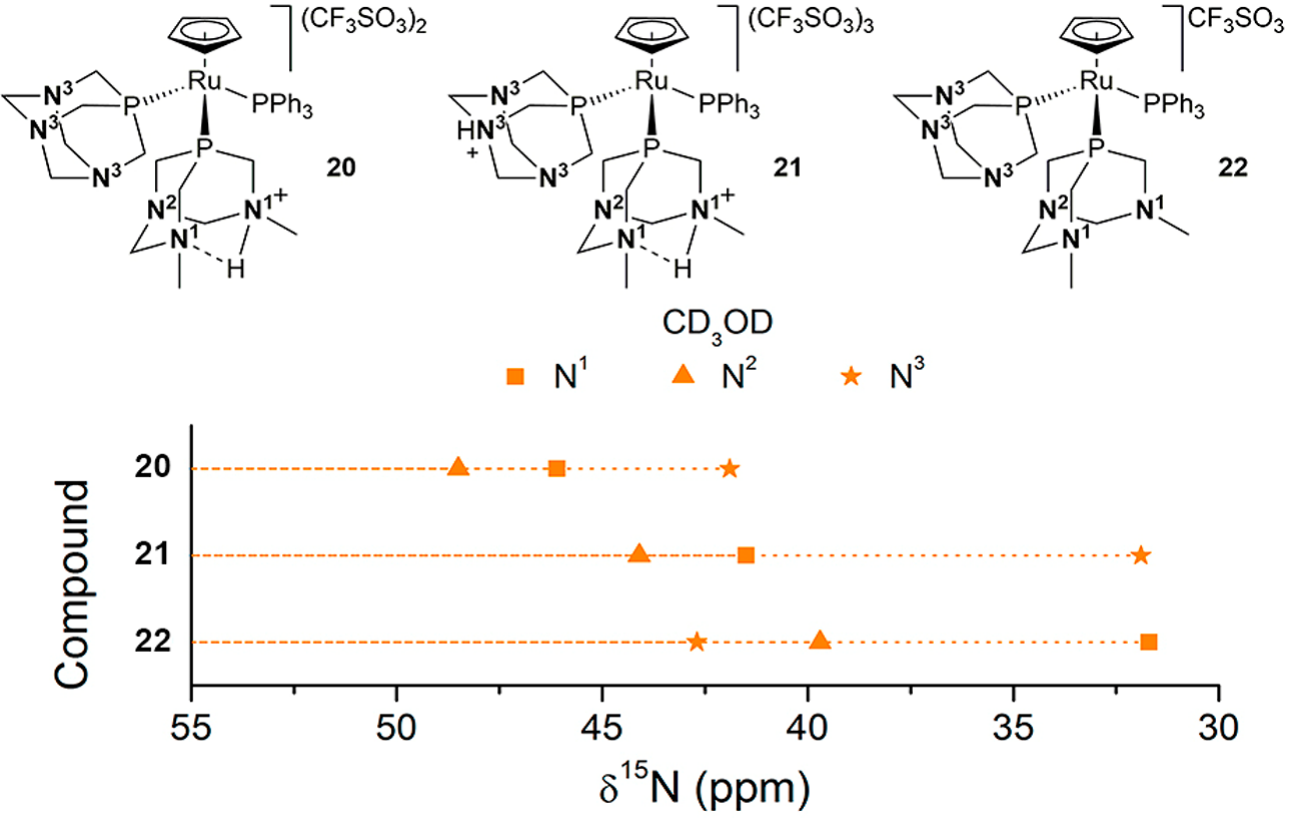
^15^N chemical shifts for **20–22**.

Complexes **20–22** (Figure 11) contain the ligand PPh_3_ and the neutral or protonated PTA and dmoPTA, providing an ideal platform for studying the effect of selective protonation on the δ^15^N of metal complexes containing these aminophosphines. The synthesis of these complexes starts from [RuCpCl(PPh_3_)(PTA)] by abstraction of the chloride with AgCF_3_SO_3_ and subsequent reaction with dmPTA (**6**). The resulting complex [RuCp(HdmoPTA)(PPh_3_)(PTA)](CF_3_SO_3_)_2_ (**20**) displays three sets of ^15^N atoms that are enumerated in Figure 11. Their chemical shifts show how the presence of the PTA shields the nitrogen atoms of HdmoPTA, producing in CD_3_OD differences in chemical shifts to **17** of Δδ^15^N^1^ = 0.8 ppm and Δδ^15^N^2^ = −1 ppm (Table S4). The nitrogen atom (N^3^) of the PTA appears at 41.9 ppm (Figure 11), which is close to those obtained for complexes **9**, **10**, and **12** in D_2_O. Complex **20** is susceptible to additional protonation on the PTA, but its HdmoPTA can be deprotonated into dmoPTA. The complex containing the protonated PTA ligand (**21**) was obtained by the addition of 1 equiv of CF_3_SO_3_H to a solution of **20** in CD_3_OD. The ^1^H–^15^N HMBC spectrum of the resulting complex shows how the protonation shifts the ^31^P multiplet corresponding to PTA to −27.09 ppm (Δδ^31^P = +12.3 ppm) and shields the N^3^ resonance by a magnitude similar to ∼31.9 ppm (Δδ^15^N^3^ = −10.0 ppm) with regard to **20**. Also, the two ^15^N signals corresponding to HdmoPTA appear at higher fields than in **20**: that for δ^15^N^1^ at 41.5 ppm and that for δ^15^N^2^ at 44.1 ppm. Complete deprotonation of **20** employing ^*t*^BuOK affords complex **22** that shows a slight deshielding of N^3^ to 42.7 ppm, while dmoPTA-N^1^ and N^2^ appear at 31.7 and 39.7 ppm, respectively, being shielded with respect to the corresponding signals in both **20** and **21**, which follow the observed trend for **17** and **18**, respectively.

## Conclusions

To shed light on the behavior of PTA and dmoPTA ligands upon κ*N*-coordination and N protonation, ruthenium half-sandwich complexes **13**, **14**, **21**, and **22** were synthesized and characterized by multinuclear NMR, IR, and single-crystal X-ray diffraction. Complexes **13** and **14** are nice examples of the complexes containing the PTA ligand as the linker between metals, providing heterometallic complexes. Both complexes, trimetallic **13** and tetrametallic **14**, possess terminal tetrahedral zinc centers κ*N*-coordinated to one or two PTA ligands, being new examples of κ*P,N*multidentation of the PTA in the solid state. It was shown that, like other previously published complexes containing PTA-κ*P,N* ligands, the Zn–N bond is not stable in solution. Complexes **21** and **22** are monometallic species containing PPh_3_, PTA, and dmoPTA in different protonation states. Their characterization by single-crystal X-ray diffraction confirmed that, upon deprotonation, dmoPTA undergoes a deep conformational change that leads to the separation of the methylated amino groups. The ^15^N chemical shifts of PTA and a representative variety of its derivatives as well as complexes (compounds **1–22**) were studied by ^1^H–^15^N HMBC NMR in various solvents. This study can be of general help to chemists working with ligands **1–8** and was performed to obtain more information about the behavior of the coordination sites of PTA and derivatives in solution. The studies supported the instability of PTA-κ*N* multimetallic complexes in solution because of the cleavage of the PTA-κ*N*-M bond, such as observed for **13–15**, and revealed it to be important and complementary to ^31^P{^1^H} NMR in assessing the dmoPTA-κ*N,N′* coordination in complexes, such as shown for **17–22**.

## Supporting Information Available

Experimental procedures, NMR data, and X-ray crystallographic data of **13**, **14**, **18**, **21**, and **22** (PDF)

## Accession Codes

CCDC 2126899 and 2126904–2126907 contain the supplementary crystallographic data for this paper. These data can be obtained free of charge via www.ccdc.cam.ac.uk/data_request/cif, or by emailing data_request@ccdc.cam.ac.uk, or by contacting The Cambridge Crystallographic Data Centre, 12 Union Road, Cambridge CB2 1EZ, UK; fax: +44 1223 336033.
