# Supplementary material for: Insights into the κ-P,N Coordination of 1,3,5-Triaza-7-phosphaadamantane and Derivatives: κ-P,N-Heterometallic Complexes and a 15N Nuclear Magnetic Resonance Survey
Source: Inorg Chem. 2022 Apr 4;61(15):5779–91. doi: 10.1021/acs.inorgchem.1c03831 (PMC9019812; doi:10.1021/acs.inorgchem.1c03831)
Supplement: Supplementary file 1 — ic1c03831_si_001.pdf [file ic1c03831_si_001.pdf]

Supporting Information

Insights on the κ-*P,N* coordination of 1,3,5-triaza-7-phosphaadamantane and derivatives: κ-*P,N*-heterometallic complexes and a ^{15}N NMR survey.

*Andrés Alguacil, Franco Scalambra, Antonio Romerosa**

Área de Química Inorgánica-CIESOL, Universidad de Almería, Almería, Spain. Emails: aaa518@ual.es, scalambra@ual.es, romerosa@ual.es

Contents

Materials and Methods……………………………………………………………..S1

Synthesis of {[(PTA)$_2$CpRu–μ-CN–1κ*C*:2κ^2*N*-RuCp(PTA)$_2$-ZnCl$_3$]}·2DMSO (**13**)..S3

Synthesis of {[ZnCl$_2$(H$_2$O)]-[(PTA-1κ*P*:2κ^2*N*)(PTA)CpRu–μ-CN–1κ*C*:2κ^2*N*-RuCp(PTA)(PTA-1κ*P*:2κ^2*N*)-[ZnCl$_2$(H$_2$O)]}Cl (**14**)…………………………………S5

Synthesis of [RuCp(HdmoPTA)(PPh$_3$)(PTA)](CF$_3$SO$_3$)$_2$ (**20**)………………………S7

Synthesis of [RuCp(HdmoPTA)(HPTA)(PPh$_3$)](CF$_3$SO$_3$)$_3$ (**21**)……………………..S12

Synthesis of [RuCp(dmoPTA)(PPh$_3$)(PTA)](CF$_3$SO$_3$) (**22**)…………………………S16

2D ^{1}H-^{15}N HMBC NMR spectra of **1-22**……………………………………………..S21

^{15}N assignments via ^{1}H-^{15}N NMR long range correlations…………………………...S37

Single crystal X-ray diffraction tables…………………………………………..……S38

References………………………………………………………………………….S50

Materials and Methods

All chemicals were of reagent grade and, unless otherwise stated, were used as received from commercial suppliers. Likewise, all reactions were carried out in a N_2 atmosphere by using standard Schlenk techniques. Solvents were deoxygenated prior to use and non-deuterated organic solvents have been dried through standard methods. Ligands PTA (**1**),[1] mPTA(I) (**2**),[2] OPTA (**3**),[3] HPTA(Cl) (**4**),[4] mPTA(CF_3SO_3) (**5**),[1] dmPTA(CF_3SO_3)$_2$ (**6**),[5] dmoPTA (**7**),[5] DAPTA (**8**)[6] (Figure S1) and complexes [RuClCp(PTA)$_2$] (**9**),[7] [RuCp(H_2O-κO)(PTA)$_2$](Cl) (**10**),[8] [RuClCp(HPTA)$_2$](Cl)$_2$ (**11**),[9] [RuCp(PTA)$_2$-μ-CN-1κC:2κ^{2}N-RuCp(PTA)$_2$](CF_3SO_3) (**12**),[10] {[{(PTA)$_2$CpRu-μ-CN–RuCp(PTA)$_2$}-μ-CdCl$_3$]}$_n$ (**15**),[11] [RuClCp(mPTA)(PPh$_3$)](CF_3SO_3) (**16**),[12] [RuCp(HdmoPTA)(PPh$_3$)$_2$](CF_3SO_3)$_2$ (**17**),[13] [RuCp(dmoPTA)(PPh$_3$)$_2$](CF_3SO_3) (**18**),[14]

$[RuCp(PPh_3)_2$-μ-dmoPTA-1κ*P*:2κ2*N*,*N'*-$ZnCl_2](CF_3SO_3)$ (**19**),[14] (Figure S2) and $[RuClCp(PPh_3)(PTA)]$[15] have been synthesized as described in literature.

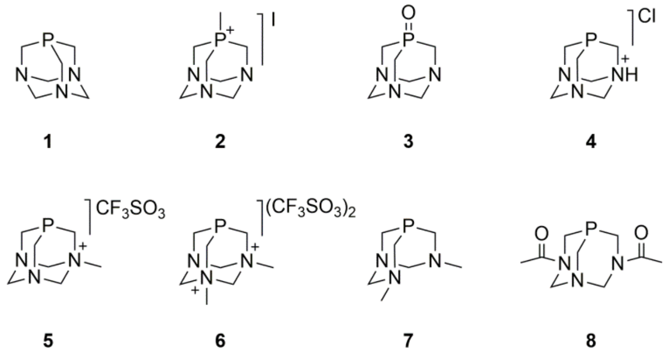

Figure S1. Ligands studied in this work.

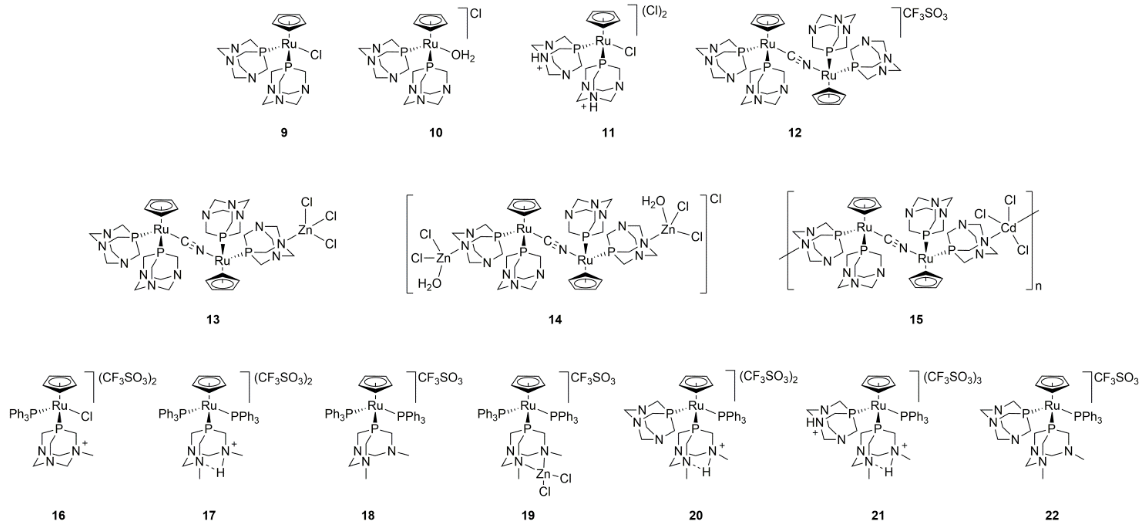

Figure S2. Complexes studied in this work.

The samples used for the NMR characterization, were prepared under inert atmosphere by dissolving in a 5 mm NMR tube 10-20 mg of compound in 0.6 mL of the desired deuterated solvent, which was previously degassed by bubbling Ar. NMR spectra were recorded with a Bruker Avance III HD 500 MHz NMR instrument operating at 500.13 MHz (^1H), 125.76 MHz (^{13}C), 202.46 MHz (^{31}P) equipped with a BBFO probe or with a

Bruker Avance III HD 600 MHz NMR instrument operating at 600.13 MHz (^{1}H), 150.92 MHz (^{13}C), 242.94MHz (^{31}P), 60.81MHz (^{15}N), equipped with a QCI-P CryoProbe™. The signals of ^{1}H and ^{13}C experiments were calibrated against internal solvent resonance, while for ^{31}P and ^{15}N external H_3PO_4 and CH_3NO_2 were used respectively. Unless otherwise stated, ^{1}H,^{15}N-HMBC experiments were carried out using the Bruker native *hmbcgpndqf* pulse sequence, setting the coupling constant at 3 Hz and collecting 2048x128 data points in spectral widths of 10 ppm and 400 ppm in F2 and F1 dimensions respectively. Each evolution increment was recorded with 8-32 scans adjusting the relaxation delay at 1.4 s. Multiplicities are indicated as follows: s (singlet), d (doublet), t (triplet), q (quartet), dd (double doublet), m (multiplet), or br (broadened). NMR peak assignments are based on the analyses of 1D ^{1}H and 2D ^{1}H-^{15}N HMBC spectra. The infrared spectra have been carried out on a Bruker Vertex 70 FT-IR spectrometer and the intensity of the bands has been indicated as: s (strong), m (medium), w (weak). Elemental analysis has been recorded on a CHNS Elementar Vario Micro Elemental Analyzer.

Single crystal X-ray diffraction was performed with a Bruker APEX-II CCD diffractometer at 100K using MoK_α (**13**, **14**) and CuK_α (**18**, **21** and **22**) radiation. Data was integrated (SAINT, Bruker) an scaled (SADABS, Bruker) and finally the structures

were solved with SHELXT[16] by using intrinsic phasing and refined with SHELXL[17] by least squares. Solution and refinement procedures were accomplished by Olex2 software.[18] Crystallographic and structural data are given in tables S5-S16. The crystal structures have been deposited at CSD with CCDC number 2126904 (**13**), 2126905 (**14**), 2126899 (**18**), 2126907 (**21**) and 2126906 (**22**).

Synthesis of {[(PTA)$_2$CpRu–μ-CN–1κ*C*:2κ^2*N*-RuCp(PTA)$_2$-ZnCl$_3$]}·2DMSO (13)

A solution of $ZnCl_2$ (11.98 mg, 0.088 mmol) in 1 mL of water were added dropwise to a solution of [RuCp(PTA)$_2$–μ-CN–1κ*C*:2κ^2*N*-RuCp(PTA)$_2$](CF_3SO_3) (**12**) (100 mg, 0.088 mmol) in 2 mL of water. The mixture was stirred for 30 minutes and a pale brown precipitate formed. This precipitate was filtered and washed with acetone (3 x 5 mL) and Et_2O (3 x 5 mL), dried under vacuum and dissolved in 0.6 mL of DMSO at 80°C. Upon cooling to room temperature, light yellow crystals of **13** formed. They were filtered and washed with acetone (3 x 5 mL) and Et_2O (3 x 5 mL). Yield: 82.3 mg (71 %). $S_{25°C,H_2O}$ = 35.7 mg/cm^3. Anal. for $C_{39}H_{70}Cl_3N_{13}O_2P_4Ru_2S_2Zn$ (1314.94 g·mol^{-1}). Calculated: C 35.64; H 5.37; N 13.86; S 4.87. Found: C 35.38; H 5.19; N 13.75; S 4.68. FT-IR (ATR, cm^{-1}): 2890 (w), 2109 (w), 1758 (m), 1645 (m), 1486 (m), 1413 (m), 1287 (m), 1233 (m),

1086 (s), 994 (s), 805 (m). ^{1}H NMR (500.13 MHz, 25°C, D_2O): 3.95 (m, 24H, N*CH_2*P_{PTA}), 4.51 (m, 24H, N*CH_2*N_{PTA}), 4.83 (s, 5H, Cp), 4.98 (s, 5H, Cp). ^{31}P{^{1}H} NMR (202.46MHz, 25°C, D_2O): δ(ppm) -19.82 (s, PTA), -22.44 (s, PTA).

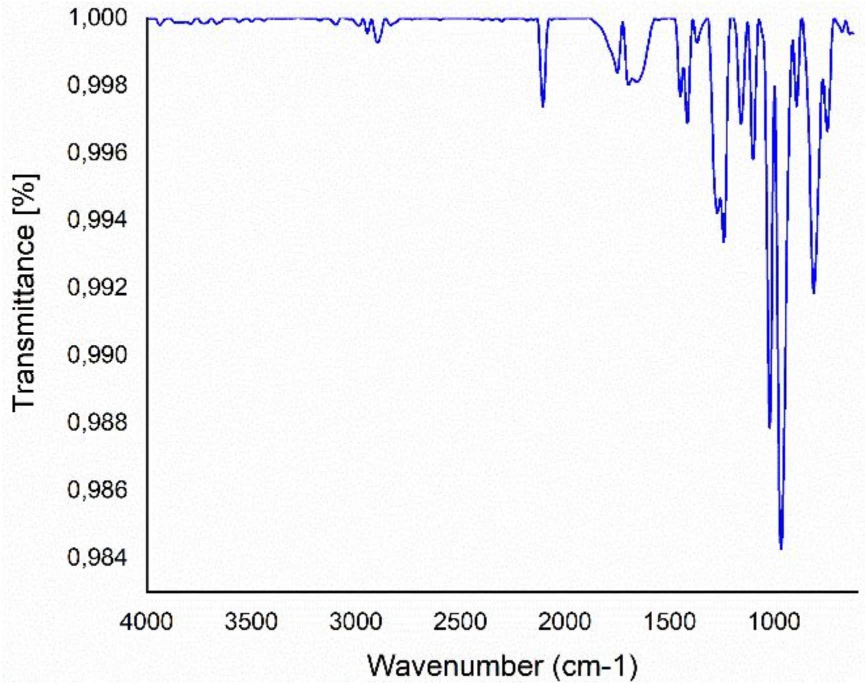

Figure S3. IR (ATR) of **13**.

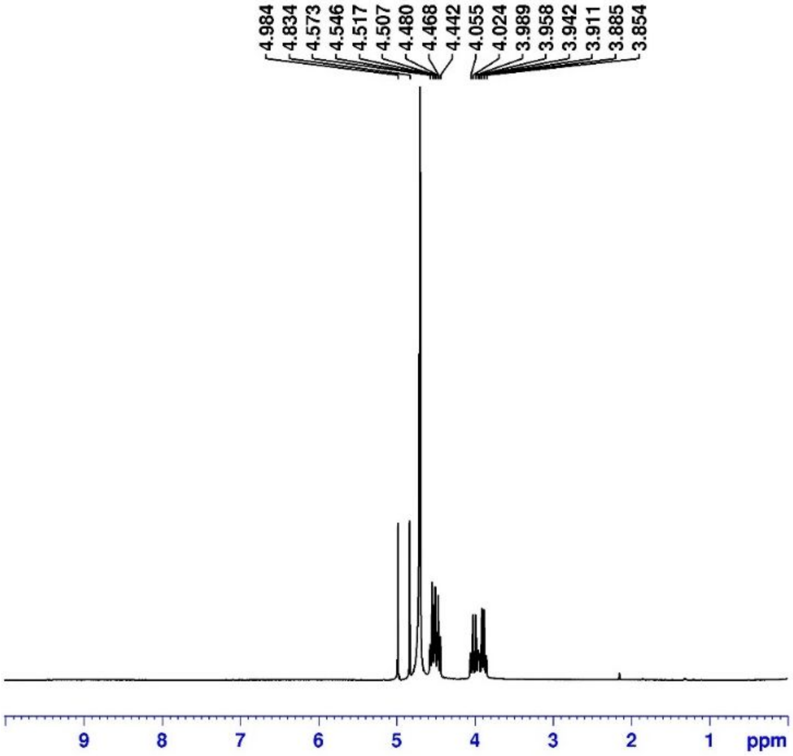

Figure S4. ^{1}H NMR (500.13 MHz, 25ºC, D_2O) of **13**.

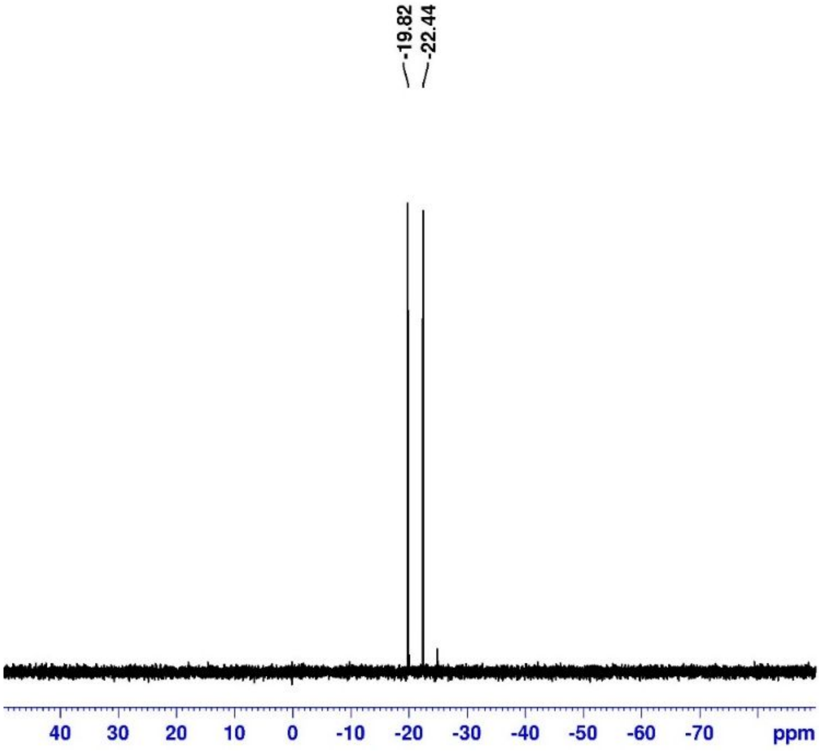

Figure S5. $^{31}P\{^{1}H\}$ NMR (202.46 MHz, 25ºC, D_2O) of **13**.

Synthesis of {[ZnCl$_2$(H$_2$O)]-[(PTA-1κ*P*:2κ^2*N*)(PTA)CpRu–μ-CN–1κ*C*:2κ^2*N*-RuCp(PTA)(PTA-1κ*P*:2κ^2*N*)-[ZnCl$_2$(H$_2$O)]}Cl (14)

Compound **14** was obtained following the same procedure used for the synthesis of **13**, but the brown precipitate obtained was dissolved in 5 mL of H_2O. Upon evaporation, light yellow crystals of **14** formed. They were filtered, washed with acetone (3 x 5 mL) and Et_2O (3 x 5 mL) and dried under vacuum. Yield: 74.8 mg (64 %). $S_{25°C,DMSO}$ = 38.3 mg/cm^3. Anal. for $C_{35}H_{62}Cl_5N_{13}O_2P_4Ru_2Zn_2$ (1330.98 g·mol^{-1}). Calculated: C 31.65; H 4.71; N 13.72. Found: C 31.49; H 4.55; N 13.75. FT-IR (ATR, cm^{-1}): 3479 (m), 2131 (w), 1623 (s), 1437 (w), 1408 (m), 1258 (m), 1235 (m), 1116 (m), 1016 (s), 989 (s), 803 (s). ^{1}H NMR (500.13 MHz, 25°C, DMSO-d$_6$): 3.96 (m, 24H, N*CH$_2$*P$_{PTA}$), 4.50 (m, 24H, N*CH$_2$*N$_{PTA}$), 4.85 (s, 5H, Cp), 5.01 (s, 5H, Cp). ^{31}P{^{1}H} NMR (202.46MHz, 25°C, DMSO-d$_6$): δ(ppm) -19.43 (s, PTA), -22.10 (s, PTA).

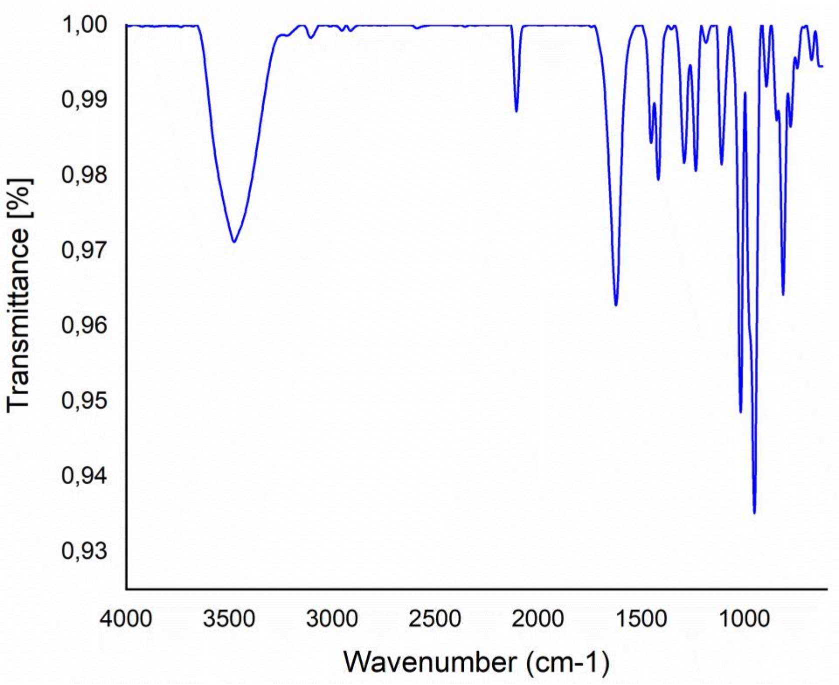

Figure S6. IR (ATR) of **14**.

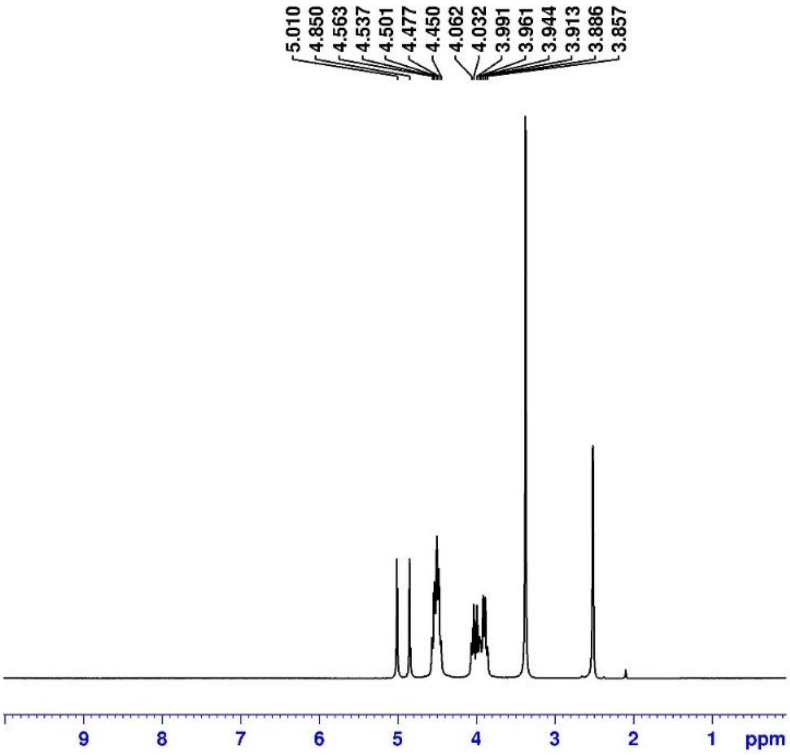

Figure S7. ^{1}H NMR (500.13 MHz, 25ºC, DMSO-d_6) of **14**.

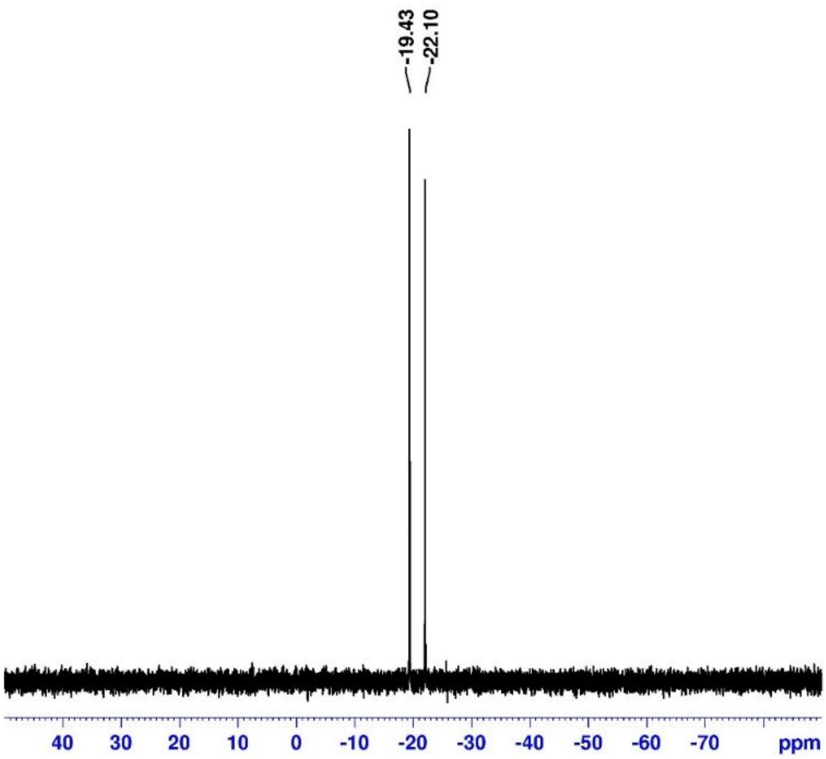

Figure S8. $^{31}P\{^{1}H\}$ NMR (202.46 MHz, 25ºC, DMSO-d_6) of **14**.

Synthesis of [RuCp(HdmoPTA)(PPh$_3$)(PTA)](CF_3SO_3)$_2$ (20)

A dissolution of $AgCF_3SO_3$ (213.3 mg, 0.83 mmol) in 30 mL of water were added dropwise into a dissolution of [RuClCp(PPh$_3$)(PTA)] (500 mg, 0.86 mmol) in 90 mL of dry MeOH. The mixture was stirred for 30 minutes protected from light. The formed white precipitate of AgCl was filtered through celite under inert atmosphere. Into the resulting filtrate the ligand dmPTA (575.94 mg, 1.11 mmol) was dissolved and the dissolution heated at 50°C for 1h, cooled to room temperature and the solvent evaporated under reduced pressure. Light brown product was filtered and washed with H_2O (3 x 20 mL), Et_2O (3 x 20 mL) and dried under air. Yield: 737.62 mg (81%). $S_{25°C,CH_3OH}$ = 38.3

mg/cm^3. Anal. for $C_{38}H_{49}F_6N_6O_6P_3RuS_2$ (1057,95 g·mol^{-1}). Calculated: C 43.15; H 4.67; N 7.94; S 6.06. Found: C 43.36; H 4.49; N 7.75; S 6.19. FT-IR (KBr, cm^{-1}): 3119 (w), 1427 (m), 1258 (s), 1020 (m). ^{1}H NMR (600.13 MHz, 25°C, CD_3OD): δ(ppm) 2.47 (d, $^2J_{HH}$ = 4.7 Hz, 6H, $CH_3N_{HdmoPTA}$), 3.05 + 3.75 + 3.84 (m + m + m, 4H, $CH_3NCH_2P_{HdmoPTA}$), 3.41 + 3.63 (m + m, 2H, $NCH_2P_{HdmoPTA}$), 3.88 + 4.04 + 4.35 (d + d + m, $^2J_{HH}$ = 11.9 Hz, $^2J_{HH}$ = 12.4 Hz, 4H, $NCH_2N_{HdmoPTA}$), 3.96 (m, 6H, NCH_2P_{PTA}), 4.50 (m, 6H, NCH_2N_{PTA}), 5.24 (s, 5H, Cp), 7.21-7.75 (m, 15H, aromatics). ^{13}C{^{1}H} NMR (150.92 MHz, 25°C, CD_3OD): δ(ppm) 41.97 (d, $^3J_{PC}$ = 4.7 Hz, $CH_3N_{HdmoPTA}$), 50.17 (d, $^1J_{PC}$ = 20.7 Hz, $NCH_2P_{HdmoPTA}$), 56.38 (d, $^1J_{PC}$ = 14.6 Hz, NCH_2P_{PTA}), 57.91 + 58.27 (d + d, $^1J_{PC}$ = 16.5 Hz, $^1J_{PC}$ = 18.2 Hz, $CH_3NCH_2P_{HdmoPTA}$), 71.44 (d, $^3J_{PC}$ = 6.9 Hz, NCH_2N_{PTA}), 74.79 + 74.86 (d + d, $^3J_{PC}$ = 3.1 Hz, $^3J_{PC}$ = 3.2 Hz, $NCH_2N_{HdmoPTA}$), 84.23 (s, Cp), 117.24-136.01 (aromatics). ^{31}P{^{1}H}NMR (242.94MHz, 25°C, CD_3OD): δ(ppm) -39.40 (m, PTA), -3.99 (dd, $^2J_{PP}$ = 35.7 Hz, HdmoPTA), 46.33 (dd, $^2J_{PP}$ = 34.4 Hz, PPh_3).

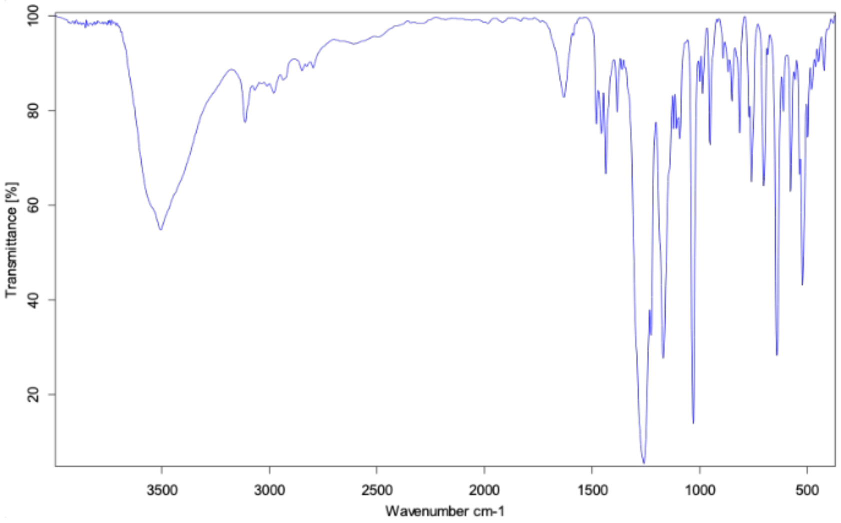

Figure S9. IR (KBr) of **20**.

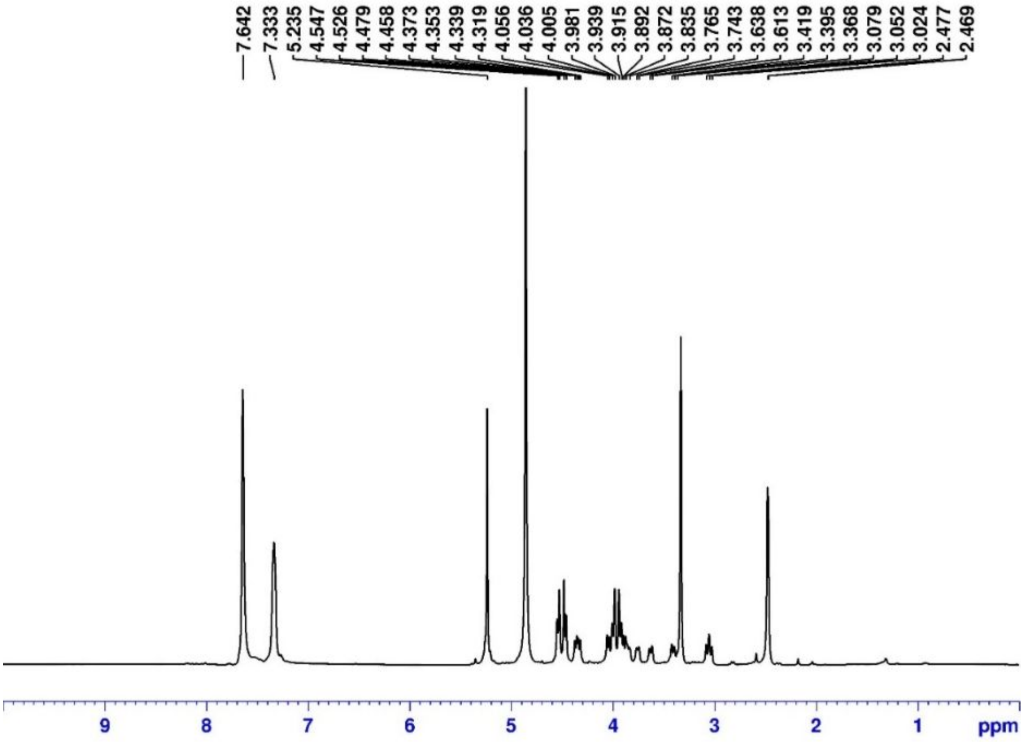

Figure S10. ^{1}H NMR (600.13 MHz, 25ºC, CD_3OD) of **20**.

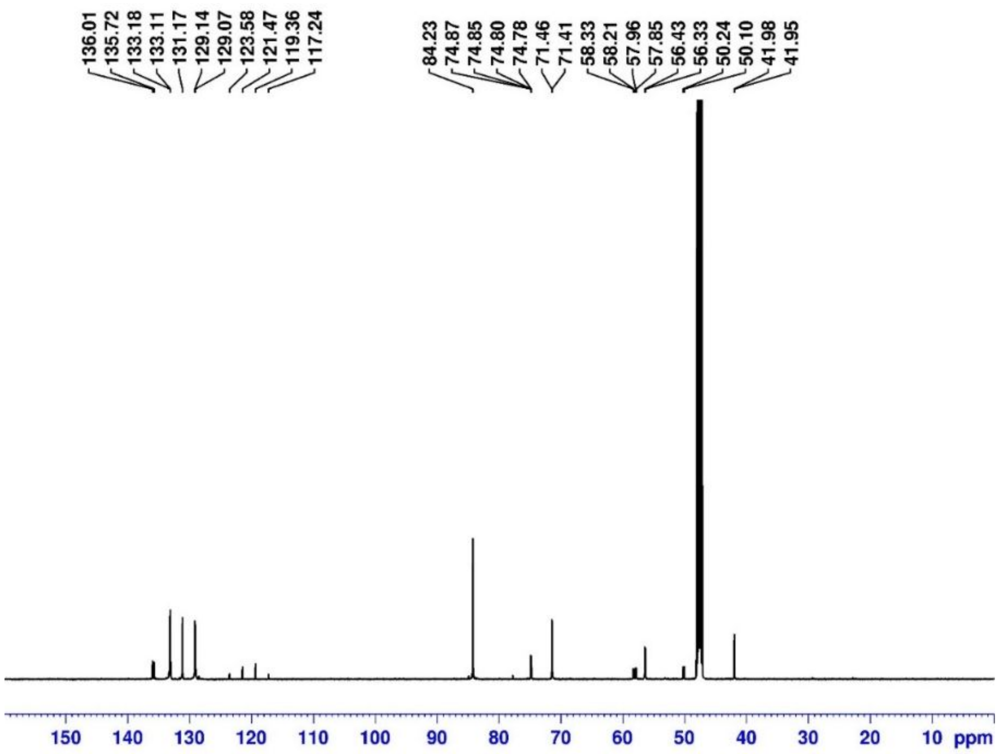

Figure S11. $^{13}C\{^{1}H\}$ NMR (150.92 MHz, 25ºC, CD_3OD) of **20**.

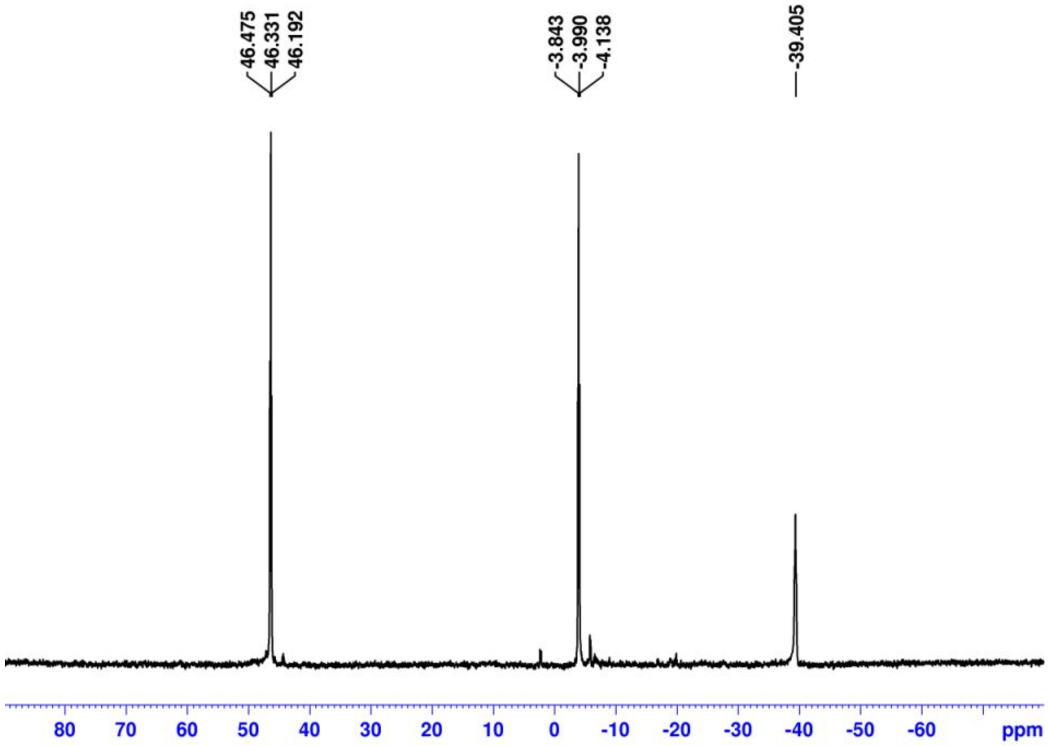

Figure S12. $^{31}P\{^{1}H\}$ NMR (242.94 MHz, 25ºC, CD_3OD) of **20**.

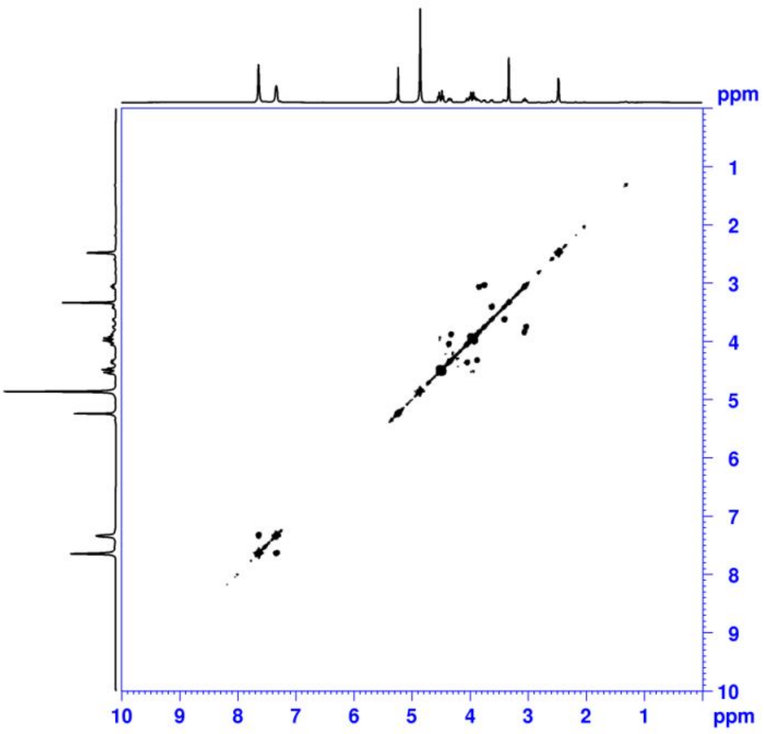

Figure S13. ^{1}H-^{1}H COSY NMR (600.13 MHz, 25ºC, CD_3OD) of **20**.

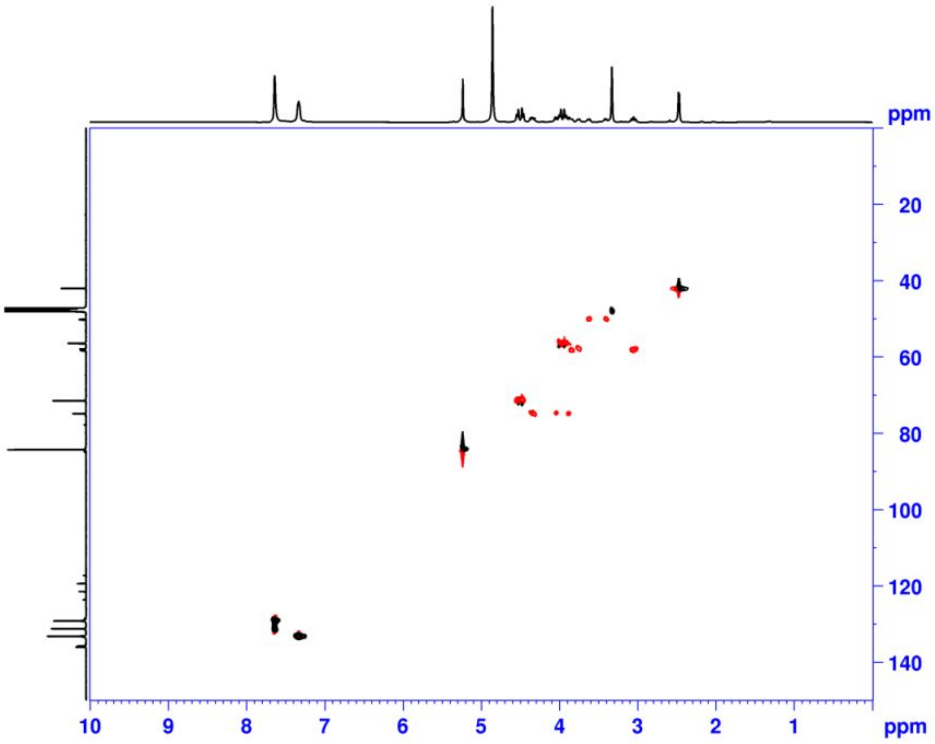

Figure S14. ^{1}H-^{13}C HSQC NMR (600.13 MHz, 150.92 MHz, 25ºC, CD_3OD) of **20**.

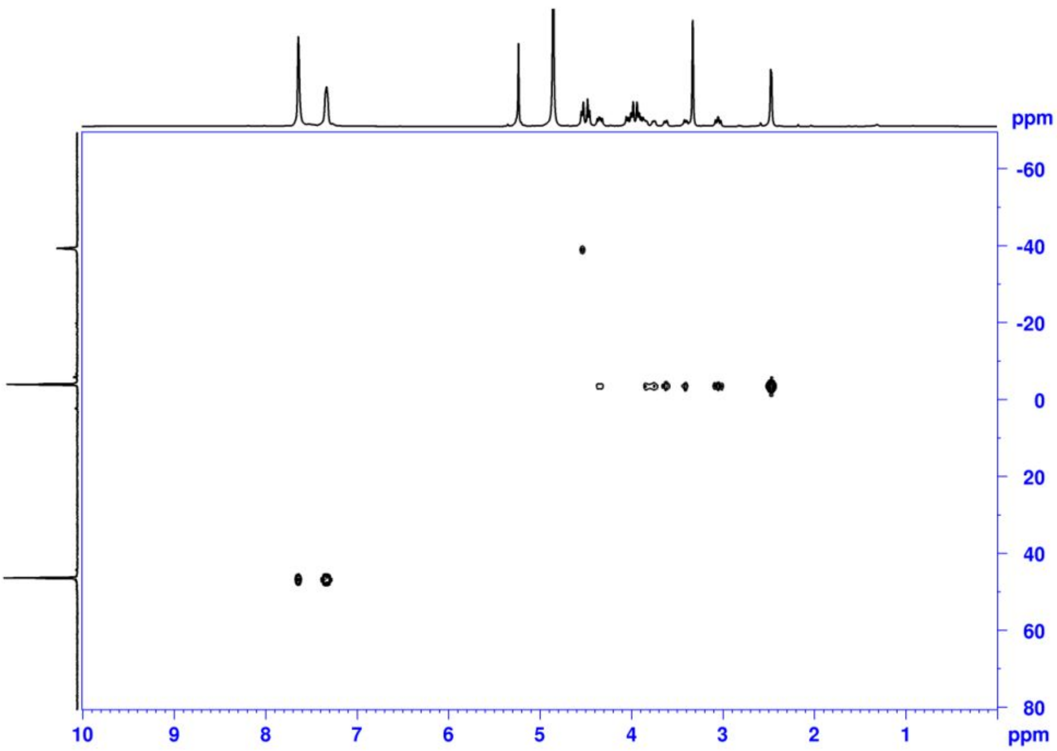

Figure S15. ^{1}H-^{31}P HMQC NMR (600.13 MHz, 242.94 MHz, 25ºC, CD_3OD) of **20**.

Synthesis of [RuCp(HdmoPTA)(HPTA)(PPh_3)](CF_3SO_3)$_3$ (21)

Into 90 mL of MeOH containing [RuCp(HdmoPTA)(PPh_3)(PTA)](CF_3SO_3)$_2$ (**20**) (100 mg, 0.095 mmol) was added dropwise a dissolution of 17 µL of CF_3SO_3H (0.19 mmol) in 5 mL of MeOH. The resulting dissolution was stirred during 30 min at room temperature and filtered. The filtrate was evaporated to 5 mL under reduced pressure and then 15 mL of Et_2O were adicionated. The yellow powder was filtered, washed with Et_2O (3 x 5 mL) and dried under vacuum. Yield: 76.82 mg (67%). $S_{25°C,CH_3OH}$ = 39.2 mg/cm^3. Anal. for $C_{39}H_{50}F_9N_6O_9P_3RuS_3$ (1208.02 g·mol^{-1}). Calculated: C 38.78; H 4.17; N 6.96;

S 7.96. Found: C 38.63; H 3.98; N 6.72; S 7.69. FT-IR (KBr, cm^{-1}): 3106 (w), 2927 (w), 1432 (m), 1263 (s), 1031 (m). ^{1}H NMR (500.13 MHz, 25°C, CD_3OD): δ(ppm) 2.48 (d, $^{2}J_{HH}$ = 13.8 Hz, 6H, $CH_3N_{HdmoPTA}$), 2.97 + 3.11 + 3.77 + 3.89 (m + m + m + m, 4H, $CH_3NCH_2P_{HdmoPTA}$), 3.46 + 3.67 (m + m, 2H, $NCH_2P_{HdmoPTA}$), 3.94 + 4.35 (d + d, $^{2}J_{HH}$ = 11.8 Hz, $^{2}J_{HH}$ = 10.2 Hz 4H, $NCH_2N_{HdmoPTA}$), 4.04 (m, 6H, NCH_2P_{HPTA}), 4.82 (m, 6H, NCH_2N_{HPTA}), 5.33 (s, 5H, Cp), 7.18-7.86 (m, 15H, aromatics). $^{13}C\{^{1}H\}$ NMR (150.92 MHz, 25°C, CD_3OD): δ(ppm) 41.9 (d, $^{3}J_{PC}$ = 4.7Hz, $CH_3N_{HdmoPTA}$), 50.05 (d, $^{1}J_{PC}$ = 21.3 Hz, $NCH_2P_{HdmoPTA}$), 53.83 (m, NCH_2P_{HPTA}), 57.54 + 58.14 (d + d, $^{1}J_{PC}$ = 17.6 Hz, $^{1}J_{PC}$ = 18.1 Hz, $CH_3NCH_2P_{HdmoPTA}$), 70.77 (bs, NCH_2N_{HPTA}), 74.77 (m, $NCH_2N_{HdmoPTA}$), 84.78 (s, Cp), 119.11-135.49 (aromatics). $^{31}P\{^{1}H\}$ NMR (242.94 MHz, 25°C, CD_3OD): δ(ppm) -27.09 (dd, $^{2}J_{PP}$ = 35.5 Hz, HPTA), -5.91 (dd, $^{2}J_{PP}$ = 35.1 Hz, HdmoPTA), 44.81 (dd, $^{2}J_{PP}$ = 34.6 Hz, PPh_3).

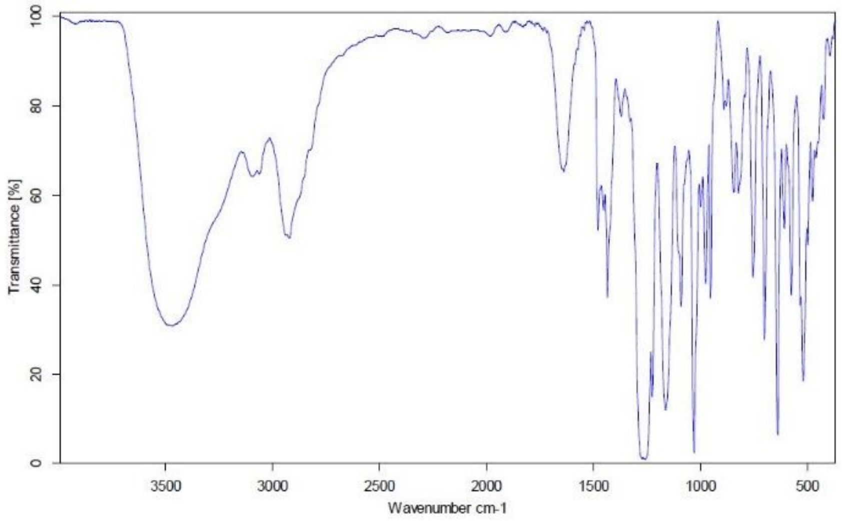

Figure S16. IR (KBr) of **21**.

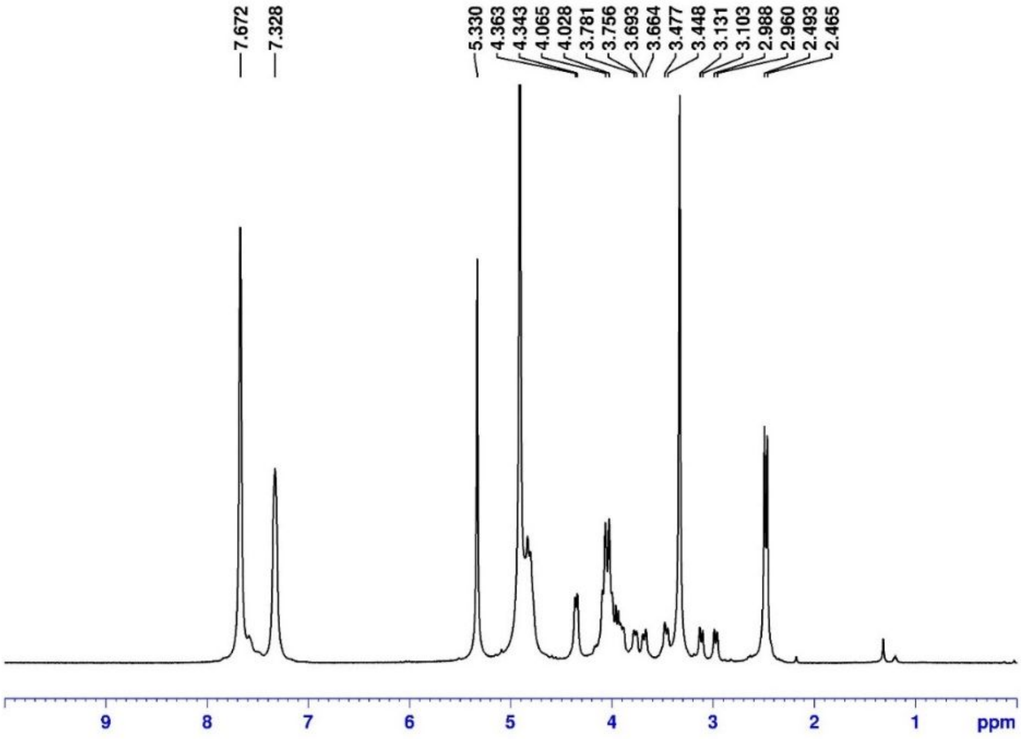

Figure S17. ^{1}H NMR (500.13 MHz, 25ºC, CD_3OD) of **21**.

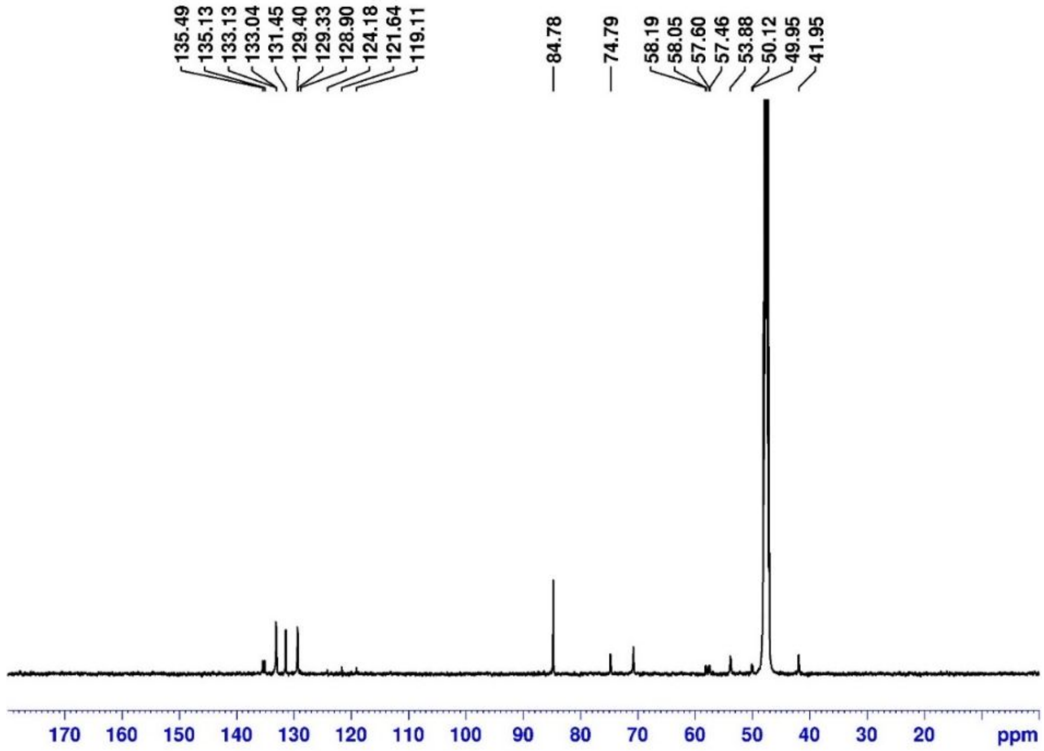

Figure S18. $^{13}C\{^{1}H\}$ NMR (125.77 MHz, 25ºC, CD_3OD) of **21**.

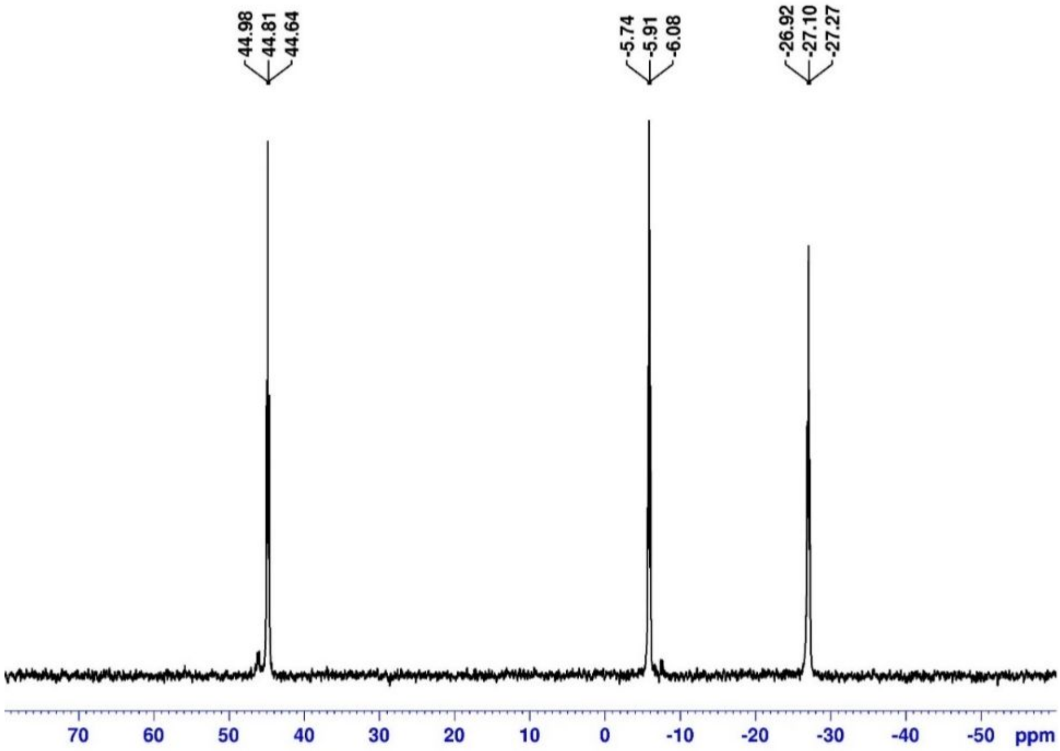

Figure S19. $^{31}P\{^{1}H\}$ NMR (202.46 MHz, 25ºC, CD_3OD) of **21**.

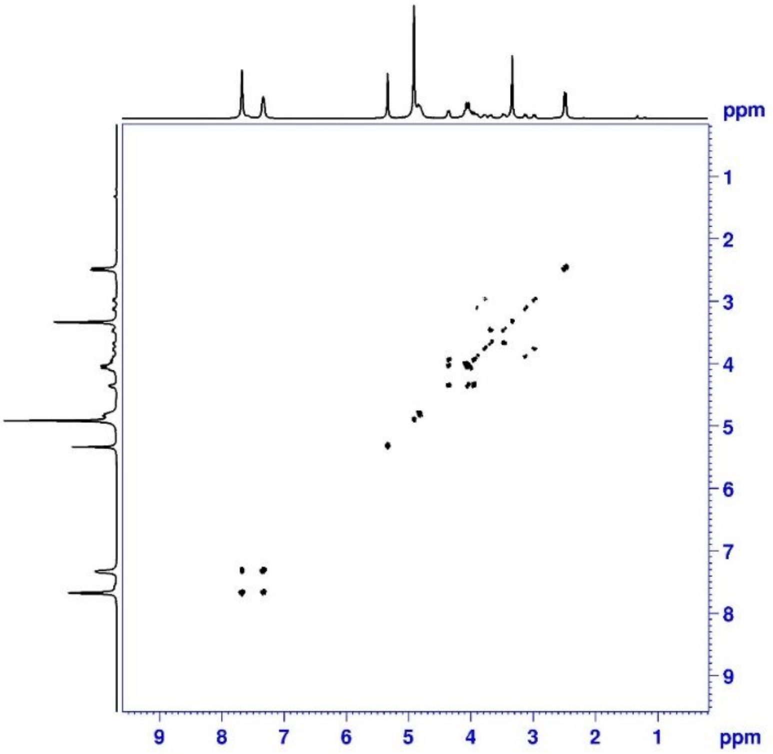

Figure S20. ^1H-^1H COSY NMR (500.13 MHz, CD_3OD, 25ºC) of **21**.

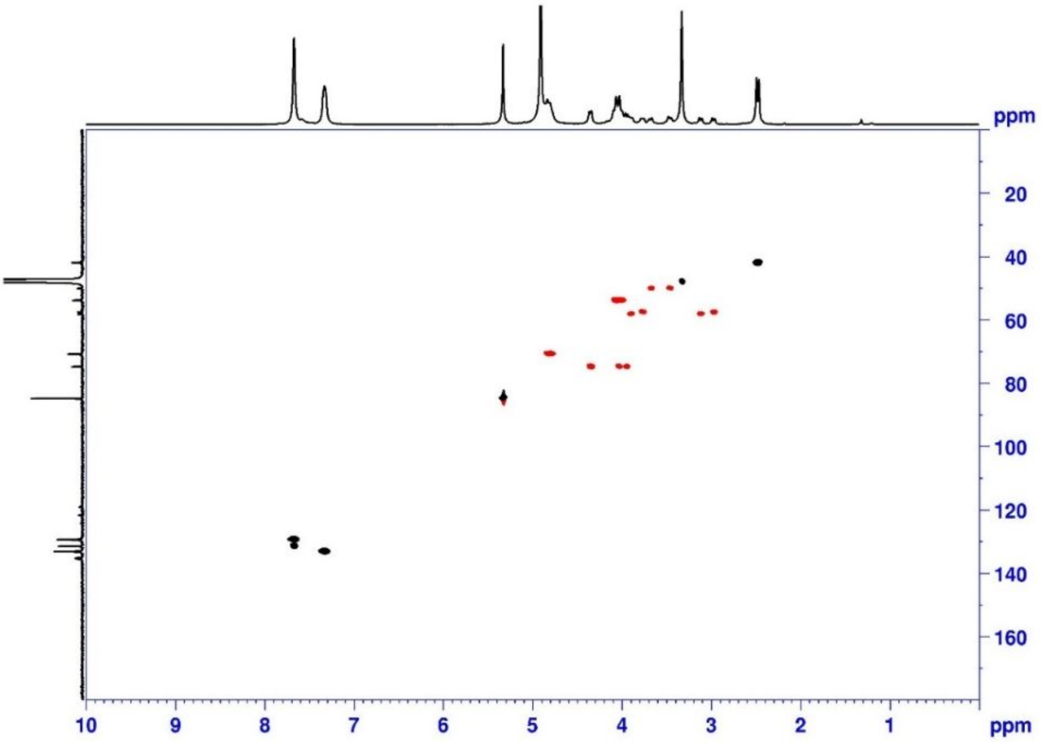

Figure S21. ^1H-^{13}C HSQC NMR (500.13 MHz, 125.77 MHz, 25ºC, CD_3OD) of **21**.

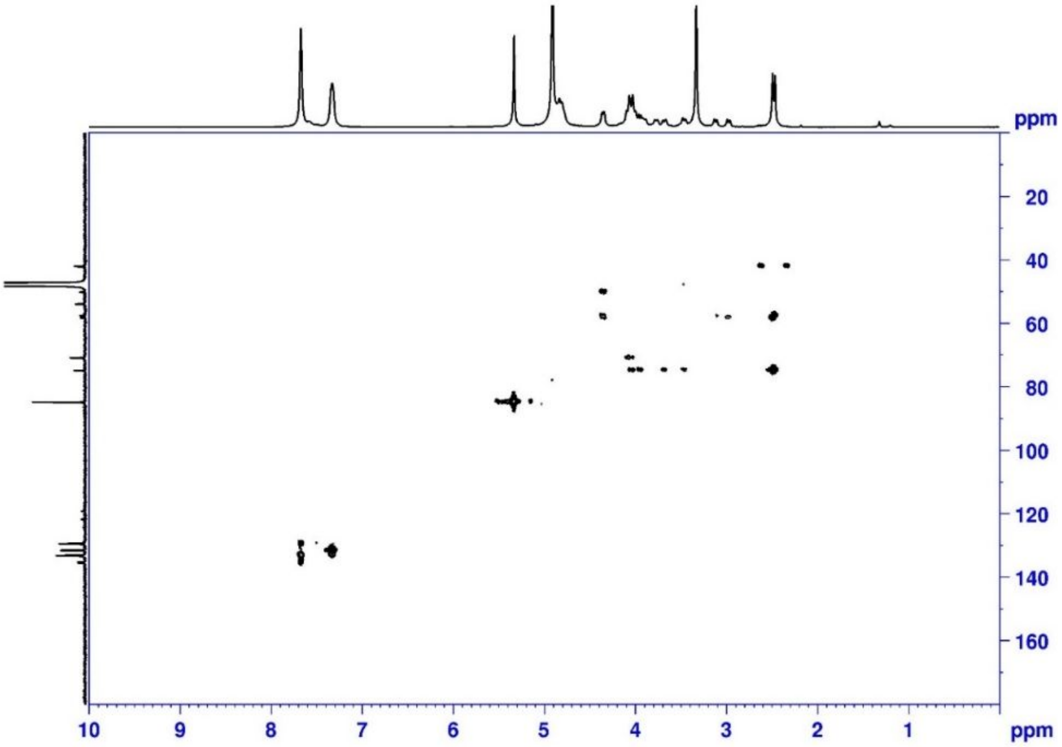

Figure S22. ^{1}H-^{13}C HMBC NMR (500.13 MHz, 125.77 MHz, 25ºC, CD_3OD) of **21**.

Synthesis of [RuCp(dmoPTA)(PPh$_3$)(PTA)](CF_3SO_3) (22)

The complex [RuCp(HdmoPTA)(PPh$_3$)(PTA)](CF_3SO_3)$_2$ (**22**) (100 mg, 0.095 mmol) was dissolved in 30 mL of dry MeOH and then tBuOK (18.62 mg, 0.166 mmol) were added under stirring. After 30 minutes the solution was evaporated to dryness under reduced pressure. The resulting powder was dissolved in 10 mL of $CHCl_3$, filtered and concentrated to 2 mL. Addition of 20 mL Et_2O precipitated a yellow powder that was filtered, washed with Et_2O (3 x 5 mL) and dried under air. Yield: 63.8 mg (74%). $S_{25°C,CH_3OH}$ = 38.9 mg/cm^3. Anal. for $C_{37}H_{48}F_3N_6O_3P_3RuS$ (907.87 g·mol^{-1}). Calculated: C 48.95; H 5.33; N 9.26; S 3.53. Found: C 48.83; H 5.12; N 8.97; S 3.26. FT-IR (KBr, cm^{-1}): 3084 (w), 2939 (w), 1435 (m), 1224 (s), 1090 (m). ^{1}H NMR (500.13 MHz, 25ºC, CD_3OD): δ(ppm) 2.18 (d, $^{2}J_{HH}$ = 2.6 Hz, 6H, CH_3N_{dmoPTA}), 2.54 + 2.65 + 3.10 + 3.19 (m

+ m + m + m, 4H, $CH_3NCH_2P_{dmoPTA}$), 2.72 + 3.06 (m+ m, 2H, NCH_2P_{dmoPTA}), 3.36 + 3.47 + 3.53 (d + d + m, $^2J_{HH}$ = 10.5 Hz, $^2J_{HH}$ = 10.7 Hz, 4H, NCH_2N_{dmoPTA}), 3.99 (m, 6H, NCH_2P_{PTA}), 4.45 (m, 6H, NCH_2N_{PTA}), 5.05 (s, 5H, Cp), 7.33-7.59 (m, 15H, m, aromatics). $^{13}C\{^1H\}$ NMR (125.77 MHz, 25ºC, CD_3OD): δ(ppm) 44.19 + 44.27 (d + d, $^3J_{PC}$ = 2.6 Hz, $^3J_{PC}$ = 8.9 Hz, CH_3N_{dmoPTA}), 47.79 (d, $^1J_{PC}$ = 21.4 Hz, NCH_2P_{dmoPTA}), 57.75 + 59.44 (d + d, $^1J_{PC}$ = 30.7 Hz, $^1J_{PC}$ = 31.2 Hz, $CH_3NCH_2P_{dmoPTA}$), 58.36 (d, $^1J_{PC}$ = 14.6 Hz, NCH_2P_{PTA}), 73.18 (d, $^3J_{PC}$ = 6.9 Hz, NCH_2N_{PTA}), 75.72 (m, NCH_2N_{dmoPTA}), 85.04 (s, Cp), 118.15-138.30 (aromatics). $^{31}P\{^1H\}$NMR (202.46 MHz, 25ºC, CD_3OD): δ(ppm) -37.54 (dd, $^2J_{PP}$ = 36.9 Hz, PTA), 5.48 (dd, $^2J_{pp}$ = 36.6 Hz, dmoPTA), 50.03 (dd, $^2J_{PP}$ = 34.6 Hz, PPh_3).

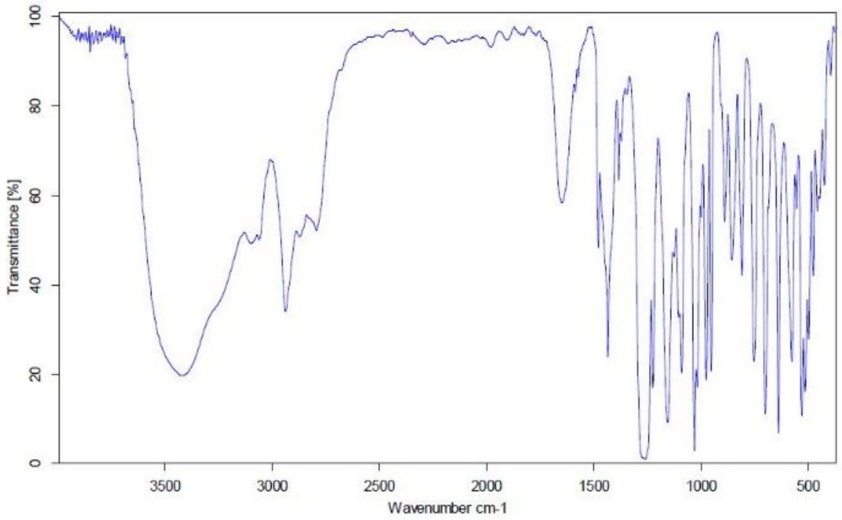

Figure S23. IR (KBr) of **22**.

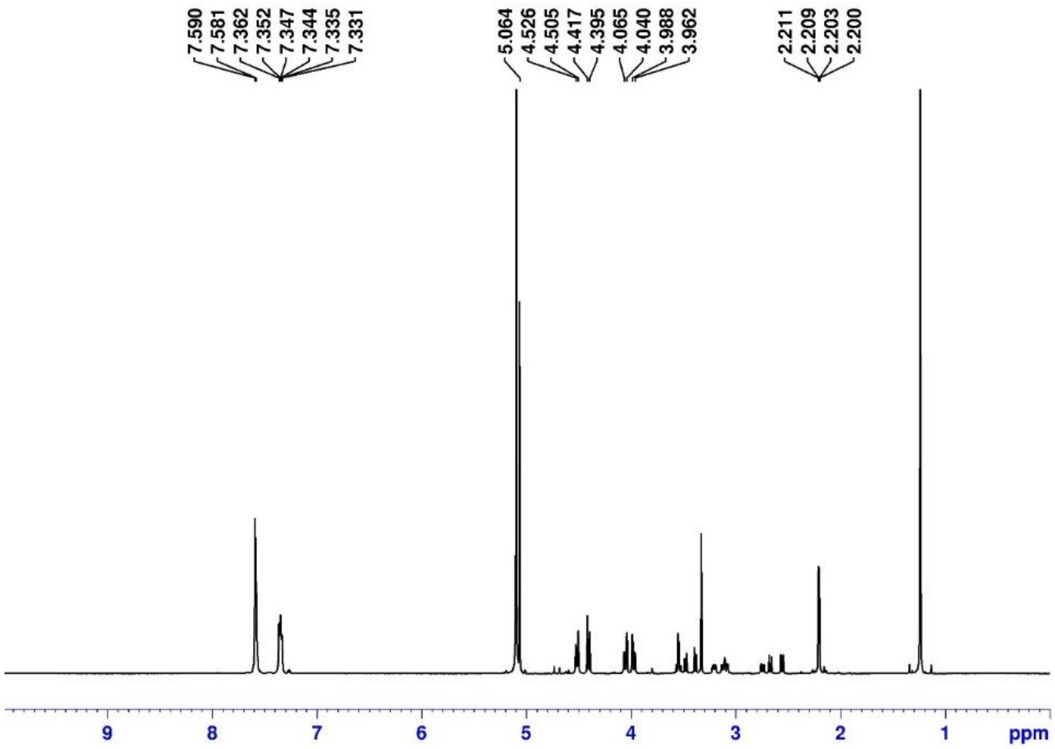

Figure S24. ^{1}H NMR (500.13 MHz, 25ºC, CD_3OD) of **22**.

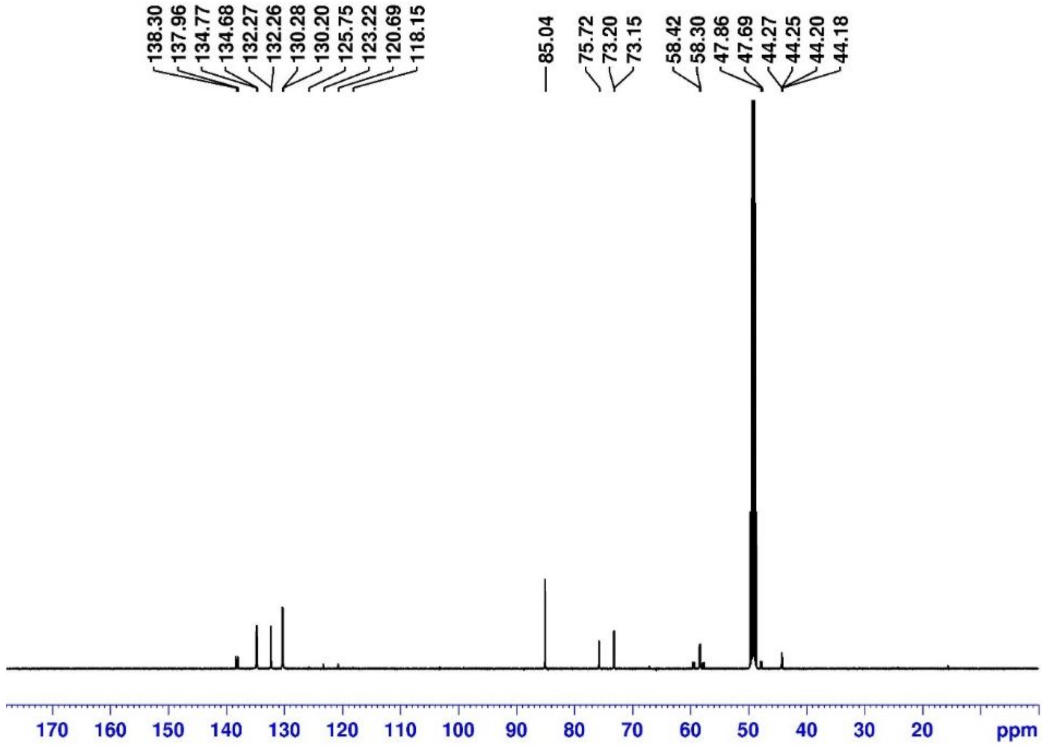

Figure S25. $^{13}C\{^{1}H\}$ NMR (125.77 MHz, 25ºC, CD_3OD) of **22**.

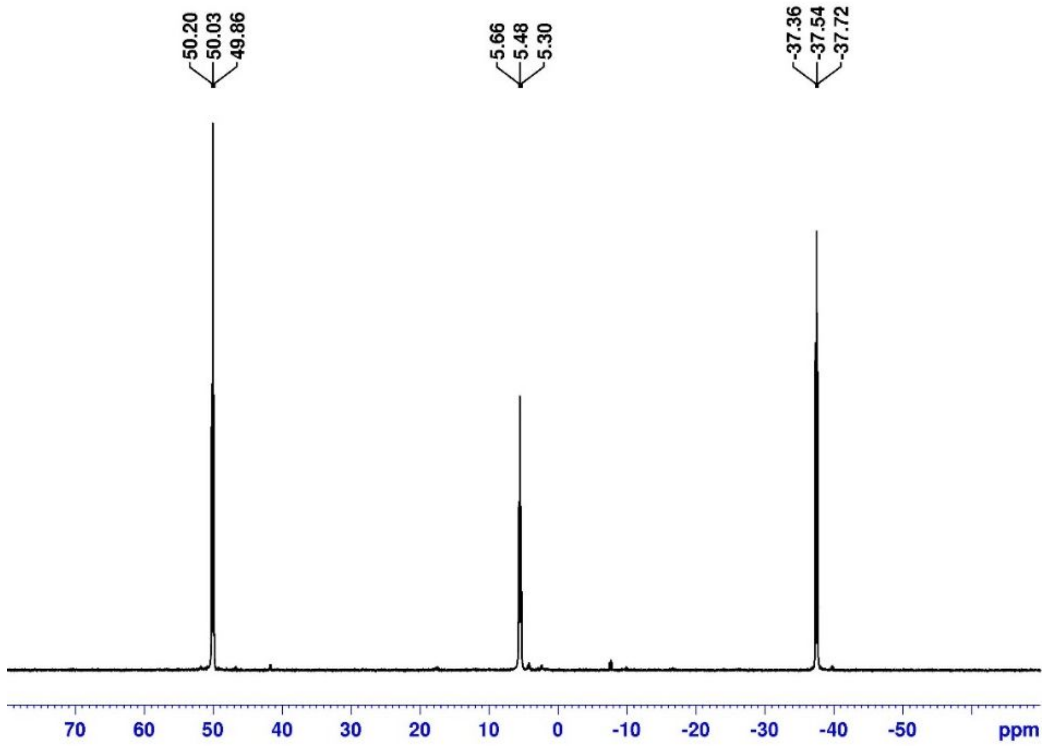

Figure S26. $^{31}P\{^{1}H\}$ NMR (202.46 MHz, 25ºC, CD_3OD) of **22**.

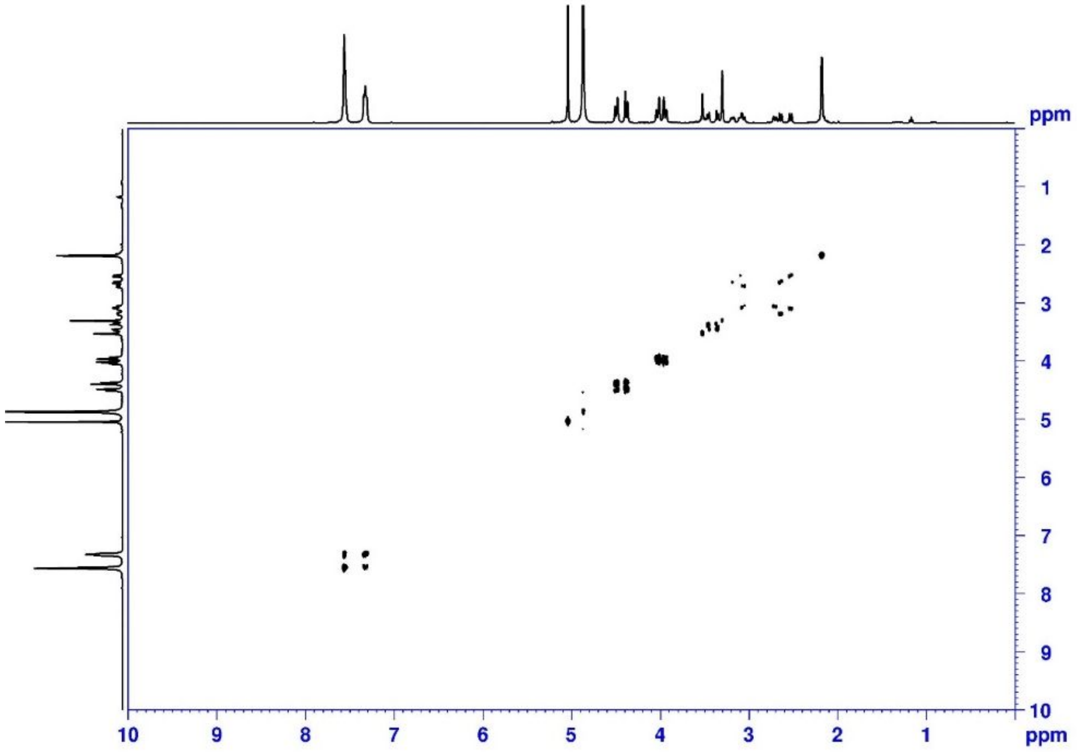

Figure S27. ^{1}H-^{1}H COSY NMR (500.13 MHz, 25ºC, CD_3OD) of **22**.

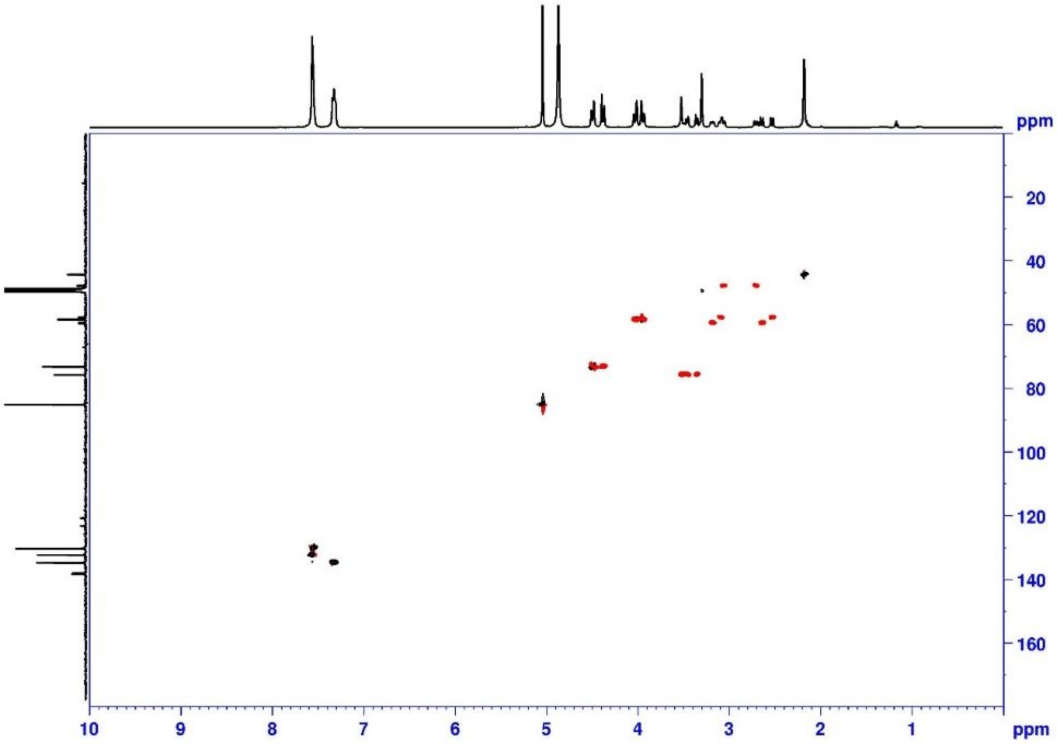

Figure S28. ^{1}H-^{13}C HSQC NMR (500.13 MHz, 125.77 MHz, 25ºC, CD_3OD) of **22**.

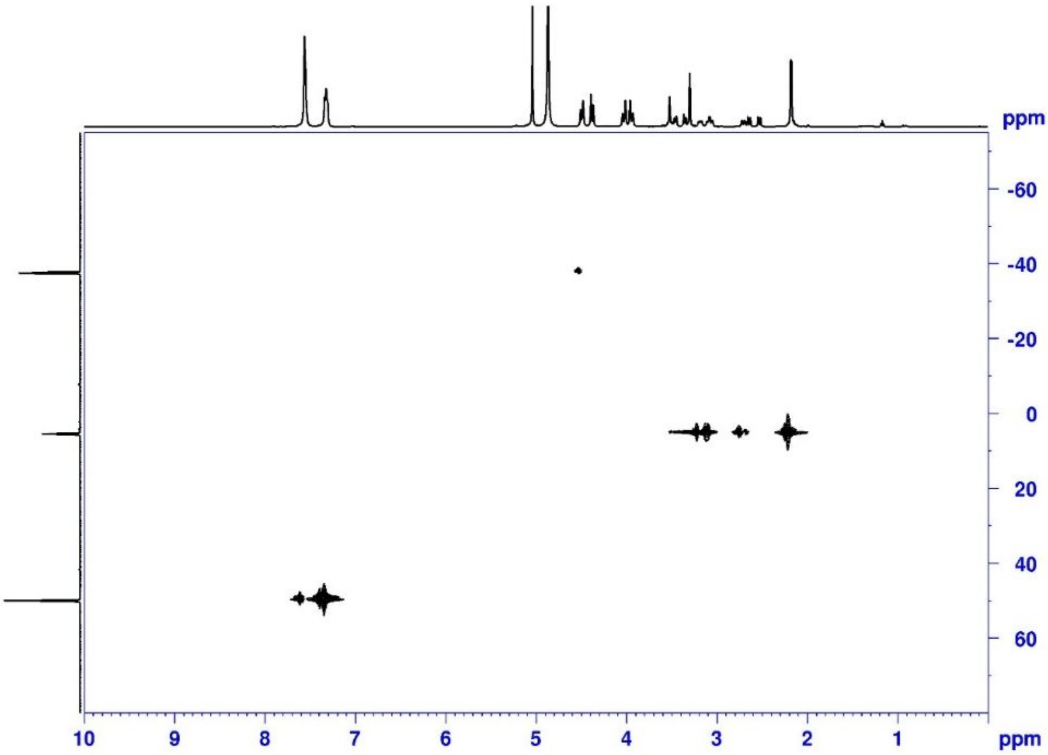

Figure S29. ^{1}H-^{31}P HMQC NMR (500.13 MHz, 202.46 MHz, 25ºC, CD_3OD) of **22**.

2D ^{1}H-^{15}N HMBC NMR spectra of 1-22

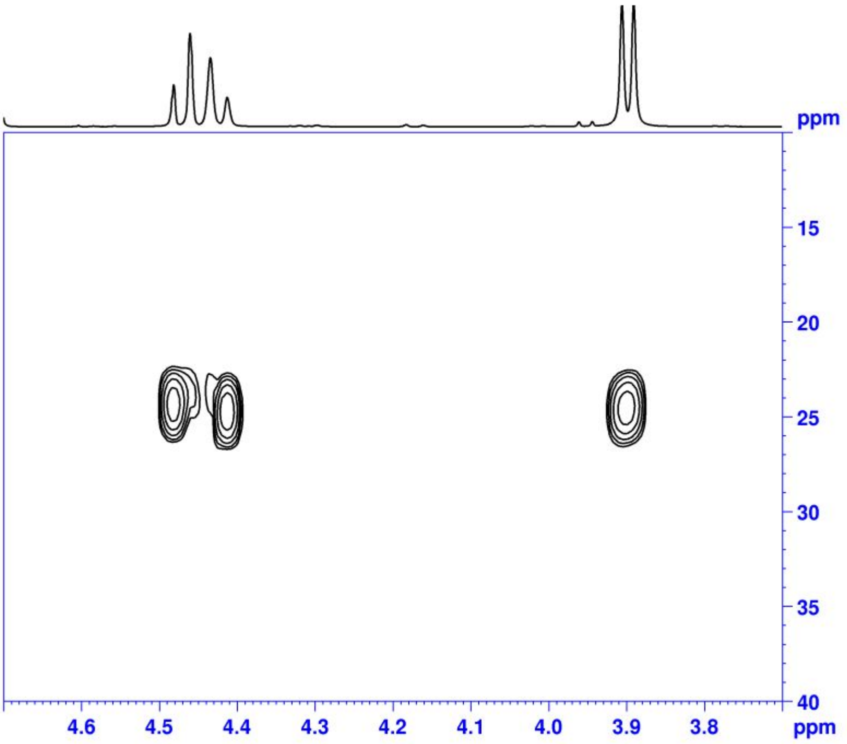

Figure S30. ^{1}H-^{15}N HMBC NMR (600.13 MHz, 60.81 MHz, 25ºC, D_2O) of **1**.

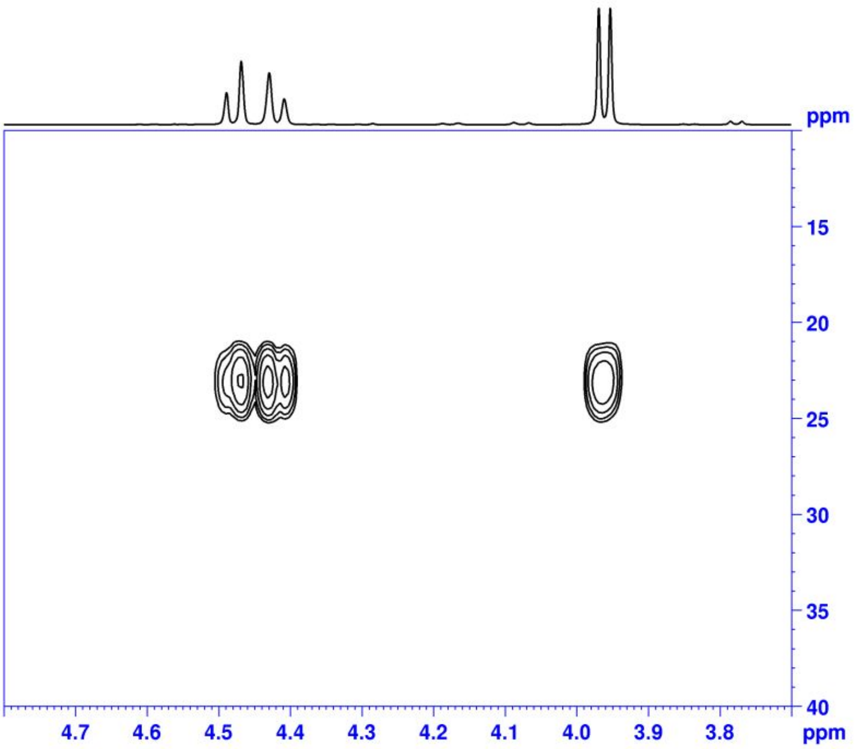

Figure S31. ^{1}H-^{15}N HMBC NMR (600.13 MHz, 60.81 MHz, 25ºC, DMSO-d_6) of **1**.

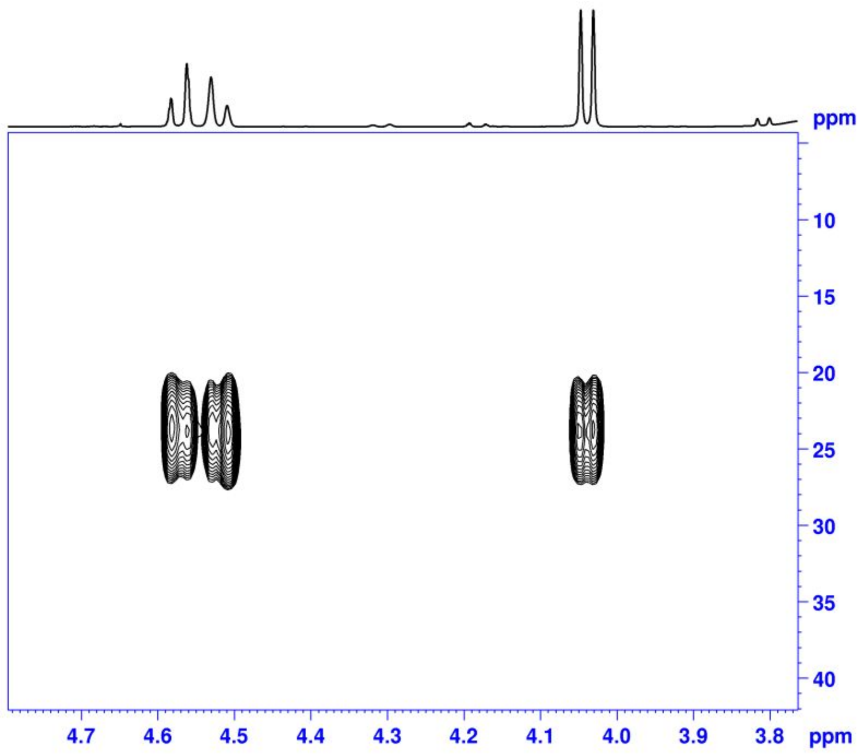

Figure S32. ^{1}H-^{15}N HMBC NMR (600.13 MHz, 60.81 MHz, 25ºC, acetone-d_6) of **1.**

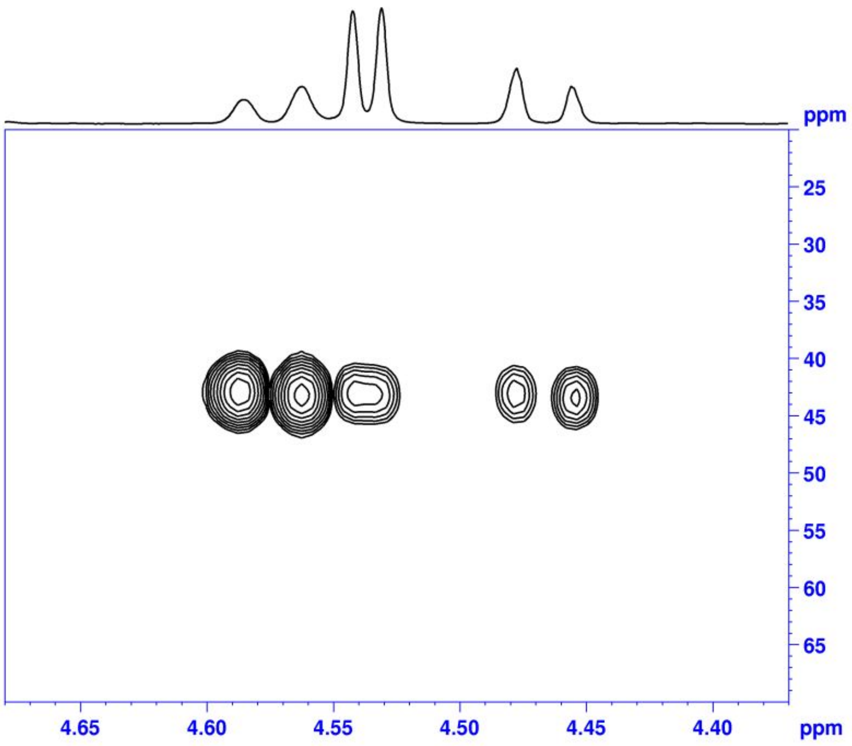

Figure S33. ^{1}H-^{15}N HMBC NMR (600.13 MHz, 60.81 MHz, 25ºC, D_2O) of **2**.

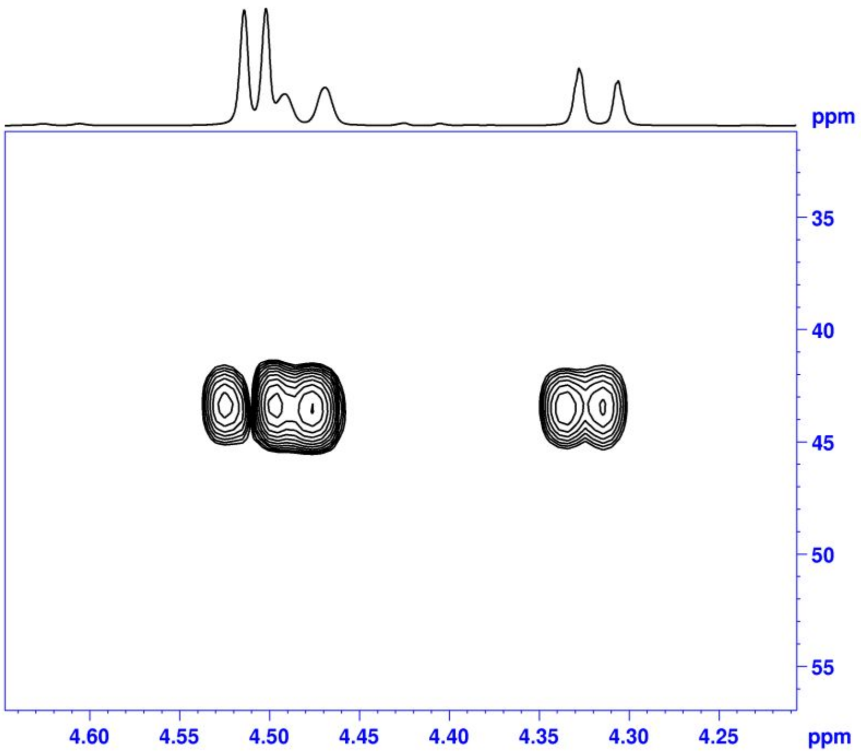

Figure S34. ^{1}H-^{15}N HMBC NMR (600.13 MHz, 60.81 MHz, 25ºC, DMSO-d_6) of **2**.

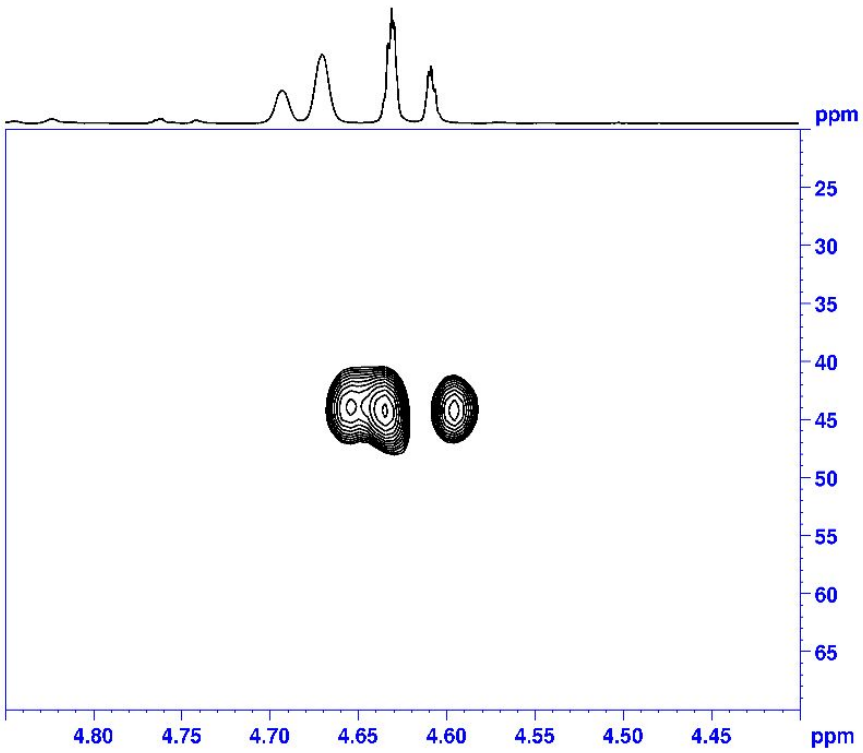

Figure S35. ^{1}H-^{15}N HMBC NMR (600.13 MHz, 60.81 MHz, 25ºC, acetone-d_6) of **2**.

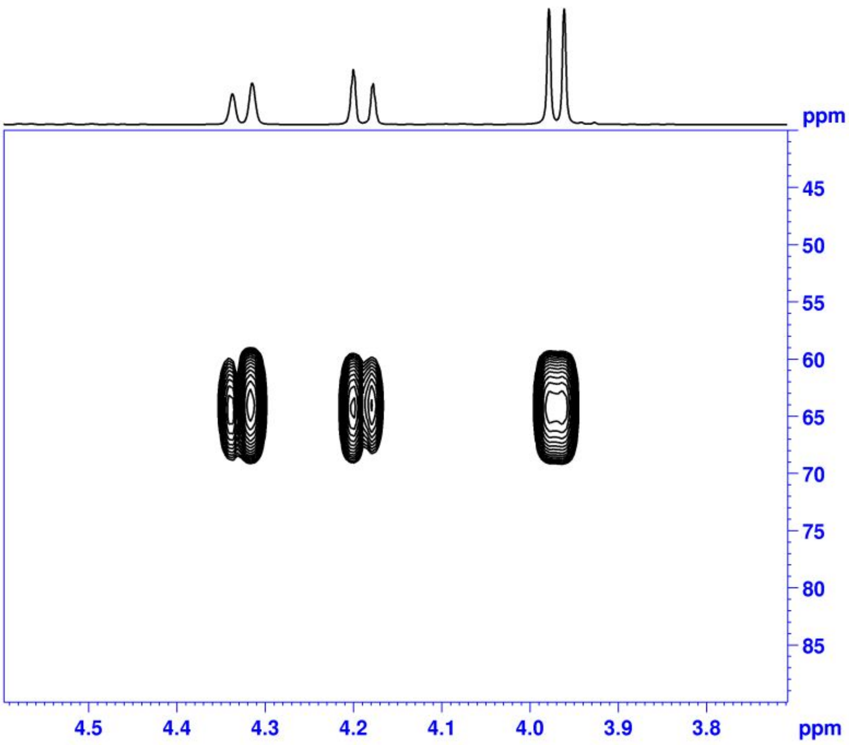

Figure S36. ^{1}H-^{15}N HMBC NMR (600.13 MHz, 60.81 MHz, 25ºC, D_2O) of **3**.

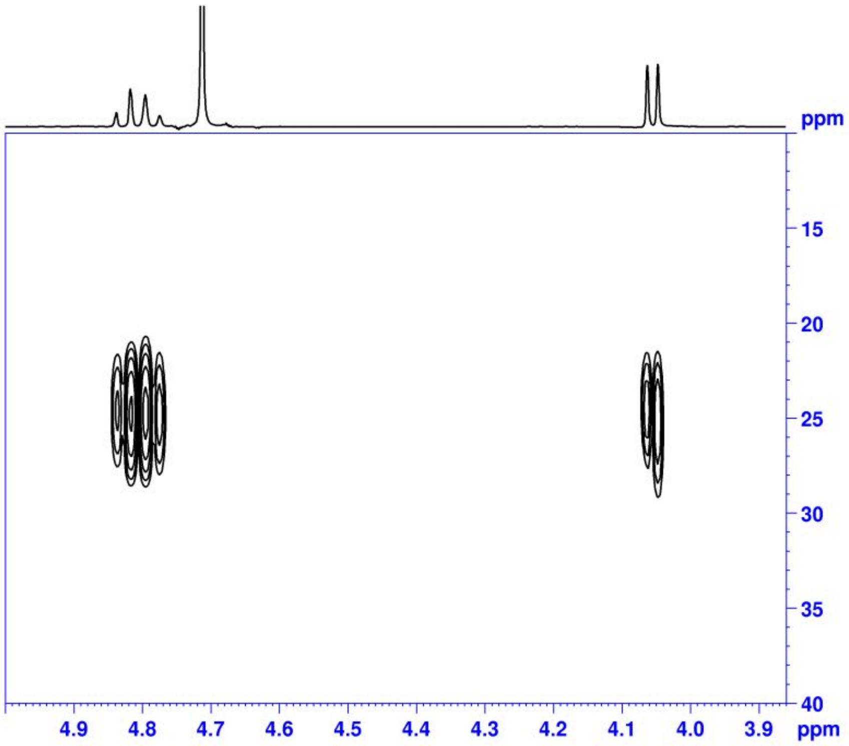

Figure S37. ^{1}H-^{15}N HMBC NMR (600.13 MHz, 60.81 MHz, 25ºC, D_2O) of **4**.

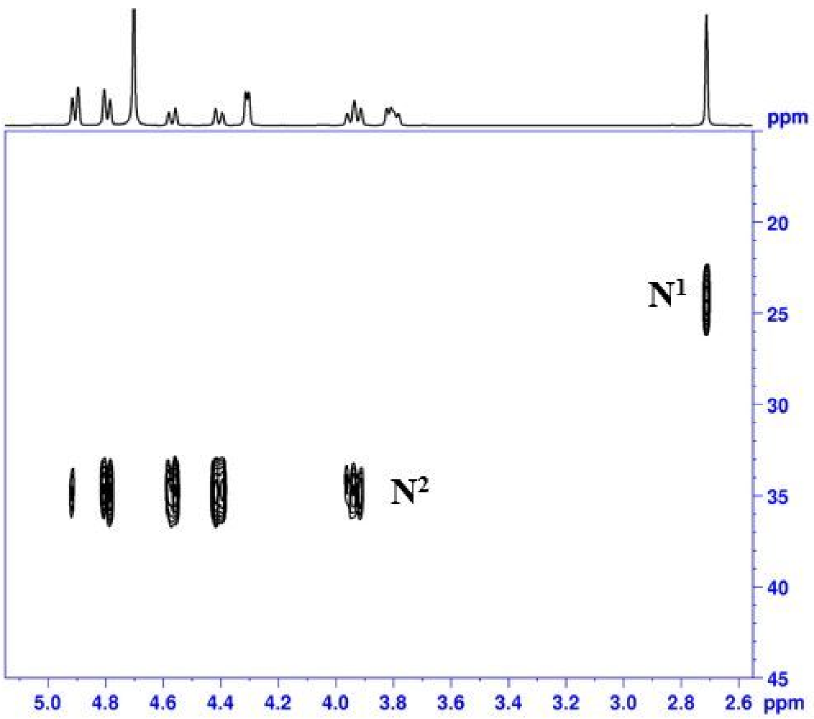

Figure S38. ^{1}H-^{15}N HMBC NMR (600.13 MHz, 60.81 MHz, 25ºC, D_2O) of **5**.

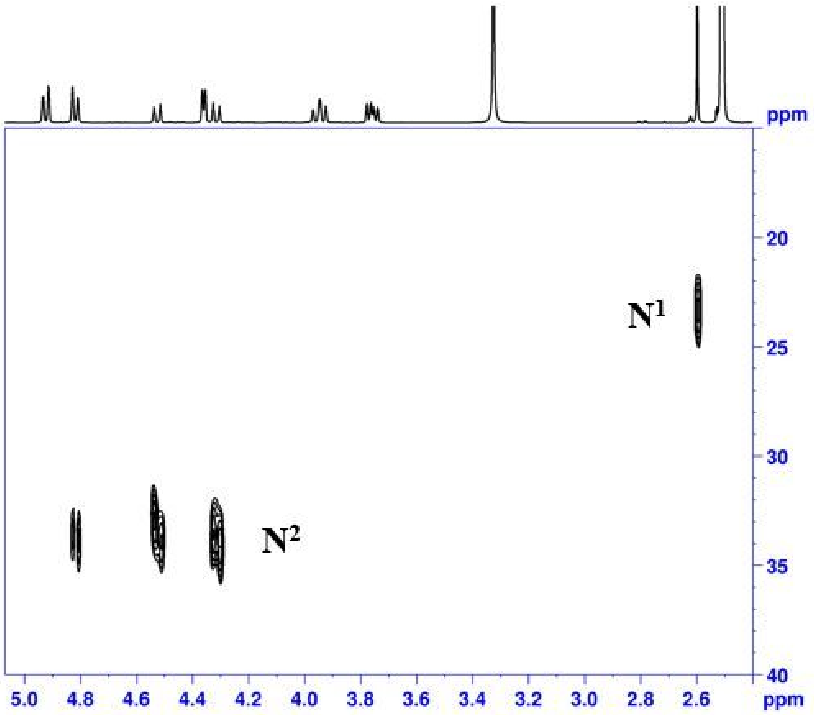

Figure S39. ^{1}H-^{15}N HMBC NMR (600.13 MHz, 60.81 MHz, 25ºC, DMSO-d_6) of **5**.

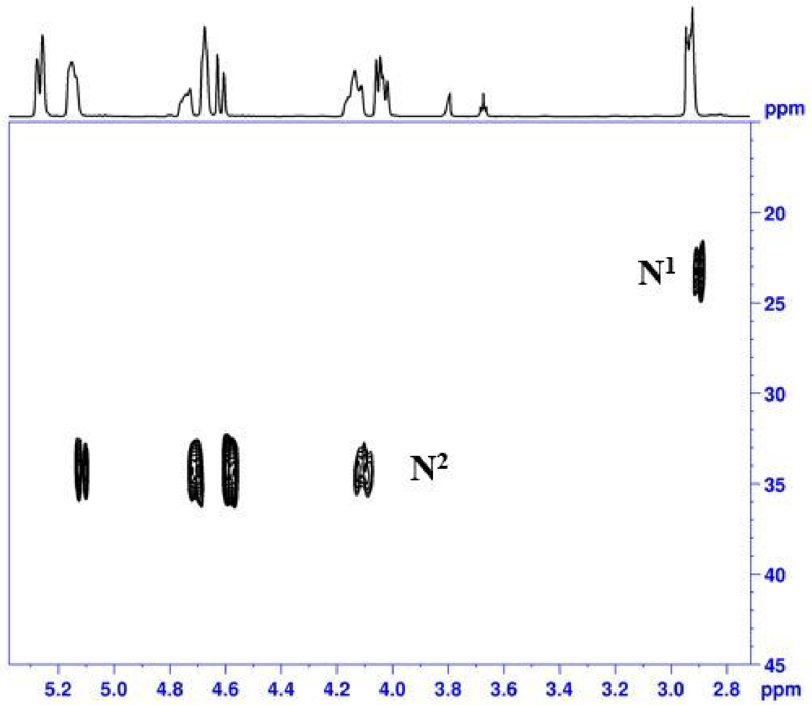

Figure S40. ^{1}H-^{15}N HMBC NMR (600.13 MHz, 60.81 MHz, 25ºC, acetone-d_6) of **5**.

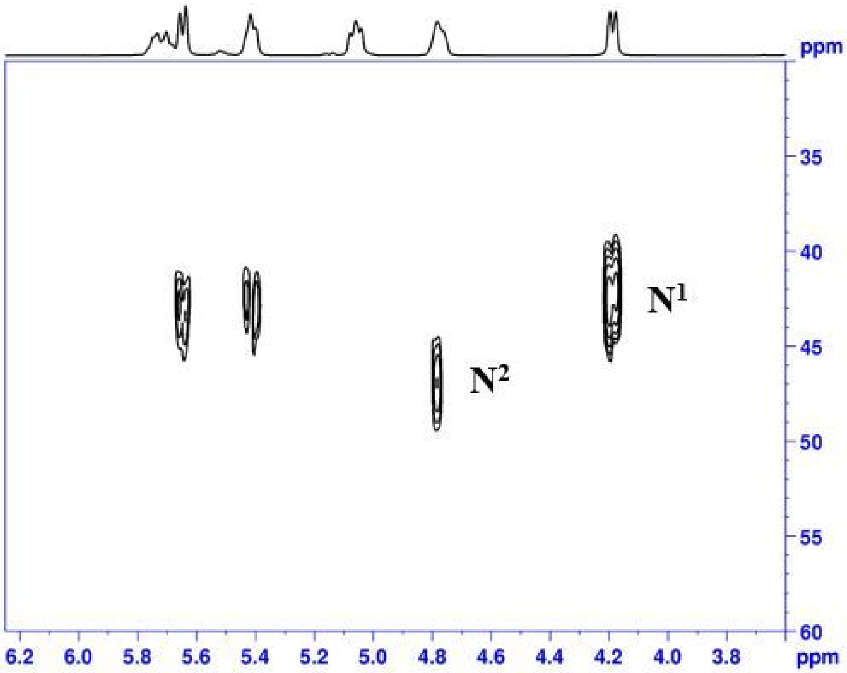

Figure S41. ^{1}H-^{15}N HMBC NMR (600.13 MHz, 60.81 MHz, 25ºC, acetone-d_6) of **6**.

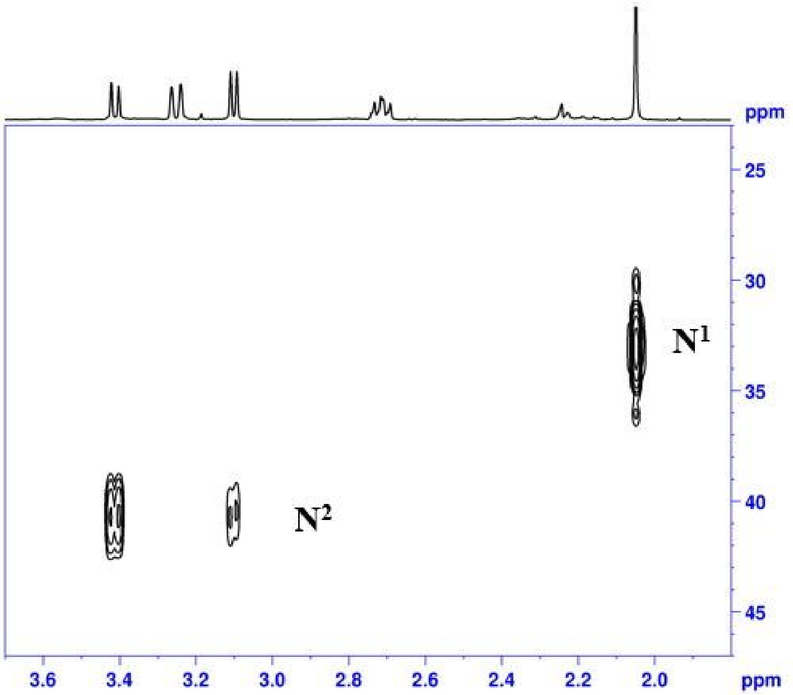

Figure S42. ^{1}H-^{15}N HMBC NMR (600.13 MHz, 60.81 MHz, 25ºC, D_2O) of **7**.

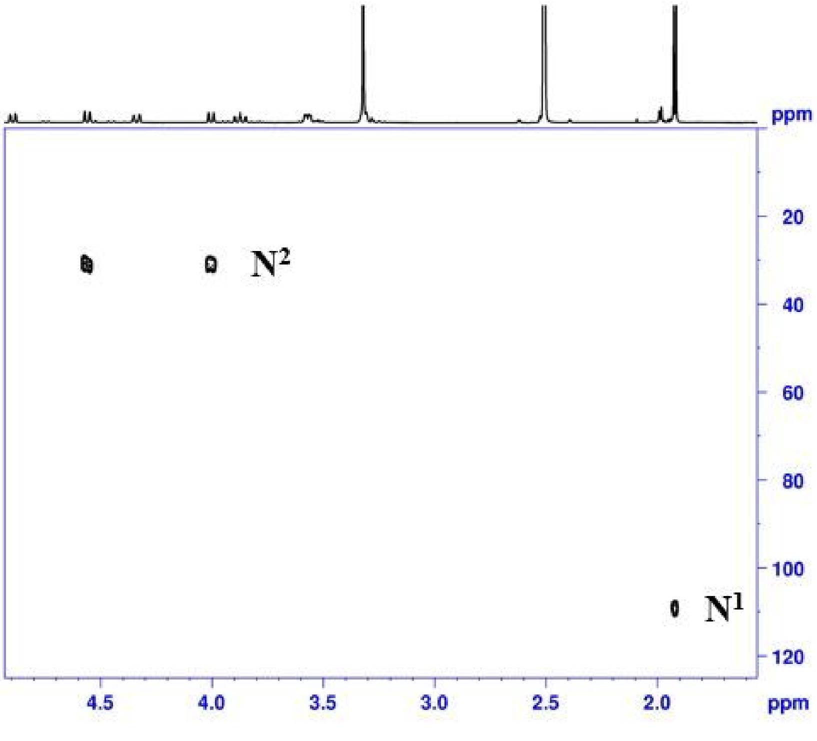

Figure S43. ^{1}H-^{15}N HMBC NMR (600.13 MHz, 60.81 MHz, 25ºC, DMSO-d_6) of **8**.

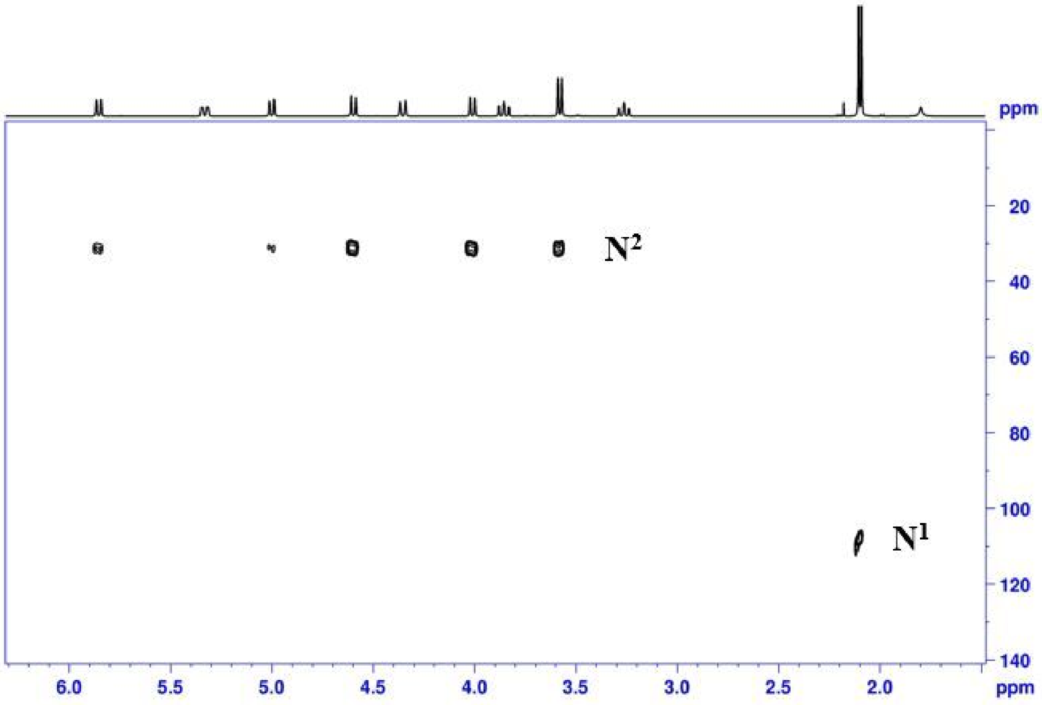

Figure S44. ^{1}H-^{15}N HMBC NMR (600.13 MHz, 60.81 MHz, 25ºC, $CDCl_3$) of **8**.

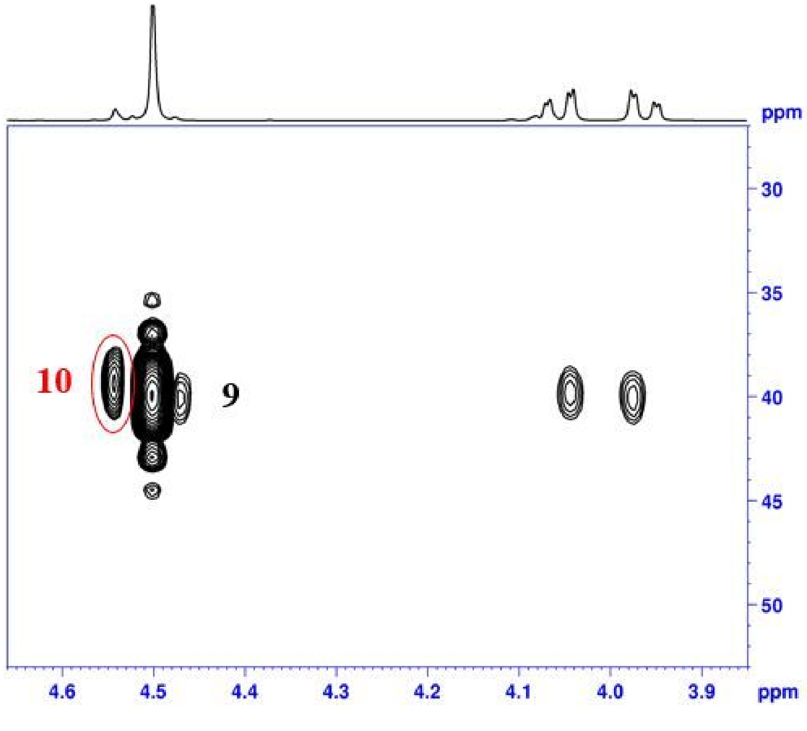

Figure S45. ^{1}H-^{15}N HMBC NMR (600.13 MHz, 60.81 MHz, 25ºC, D_2O) of **9** and **10**.

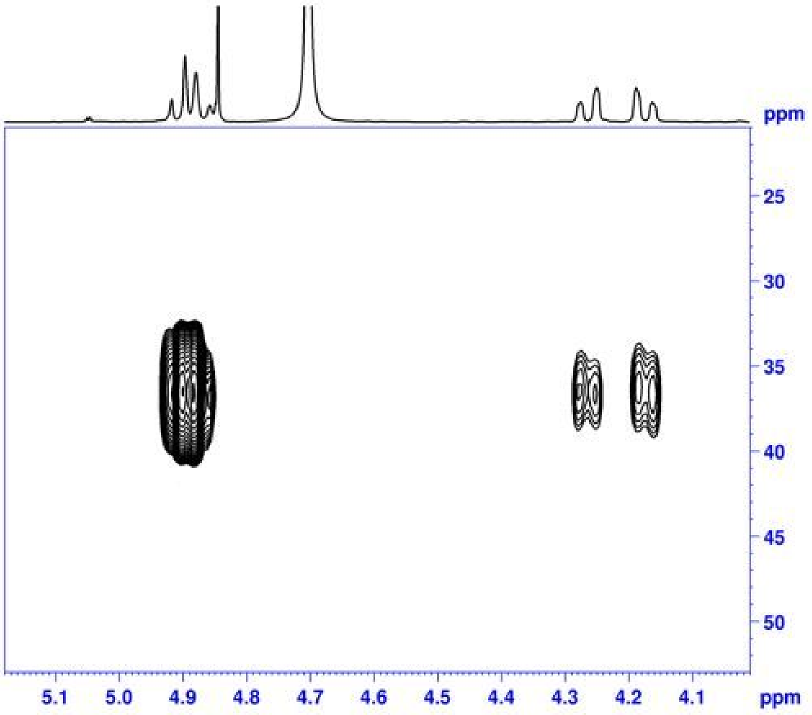

Figure S46. $^{1}H,^{15}N$ HMBC NMR (600.13 MHz, 60.81 MHz, 25ºC, D_2O) of **11**.

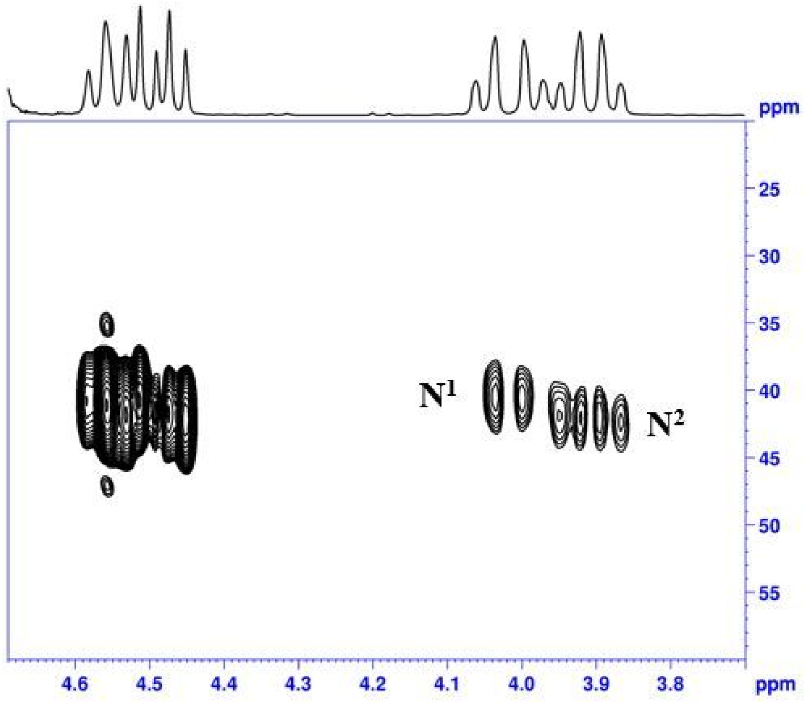

Figure S47. $^{1}H-^{15}N$ HMBC NMR (600.13 MHz, 60.81 MHz, 25ºC, D_2O) of **12**.

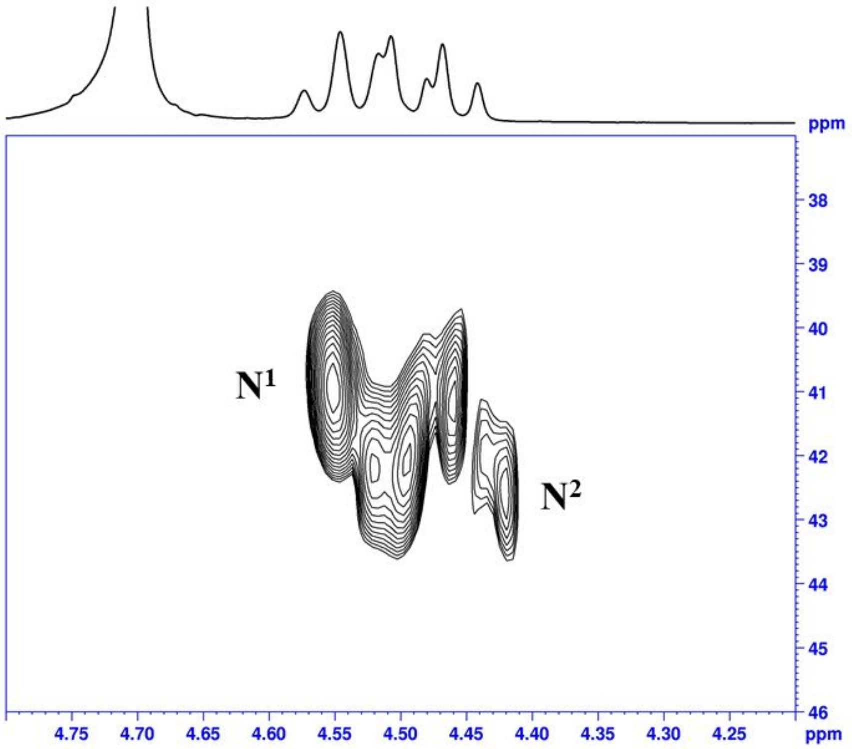

Figure S48. ^{1}H-^{15}N HMBC NMR (500.13 MHz, 50.68 MHz, 25ºC, D_2O) of **13.**

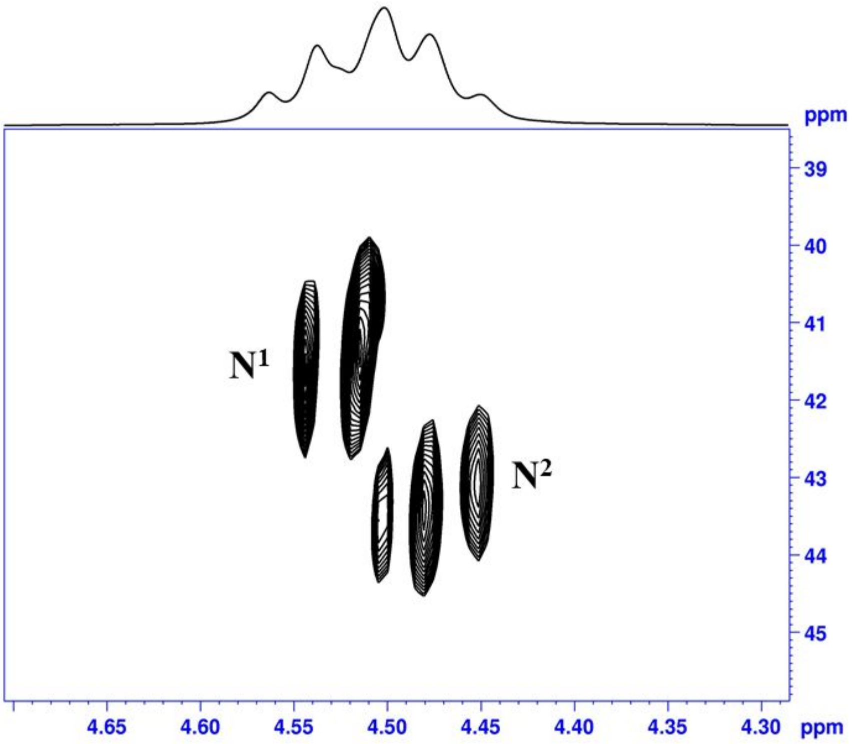

Figure S49. ^{1}H-^{15}N HMBC NMR (600.13 MHz, 50.68 MHz, 25ºC, DMSO-d_6) of **14.**

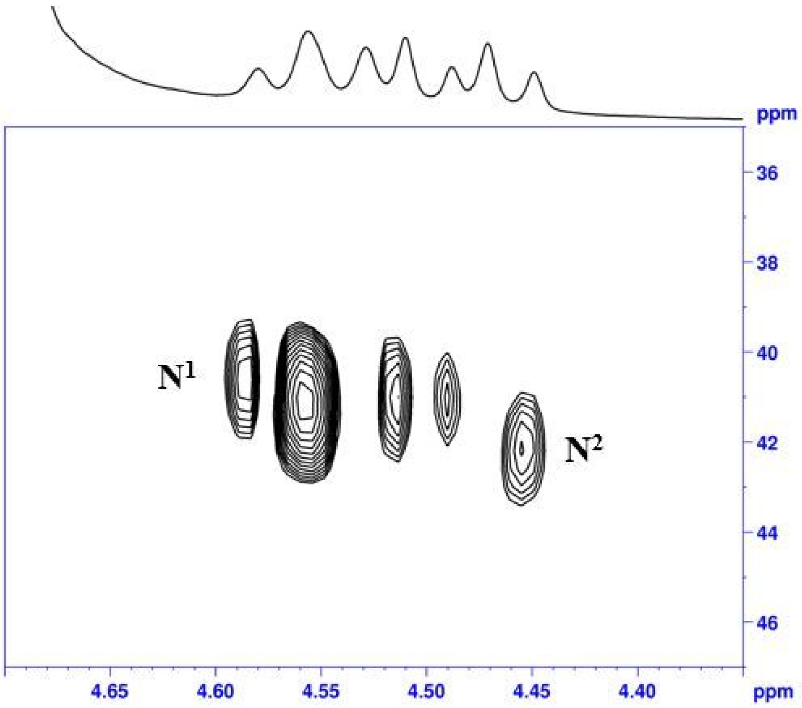

Figure S50. ^{1}H-^{15}N HMBC NMR (600.13 MHz, 60.81 MHz, 25ºC, D_2O) of **15**.

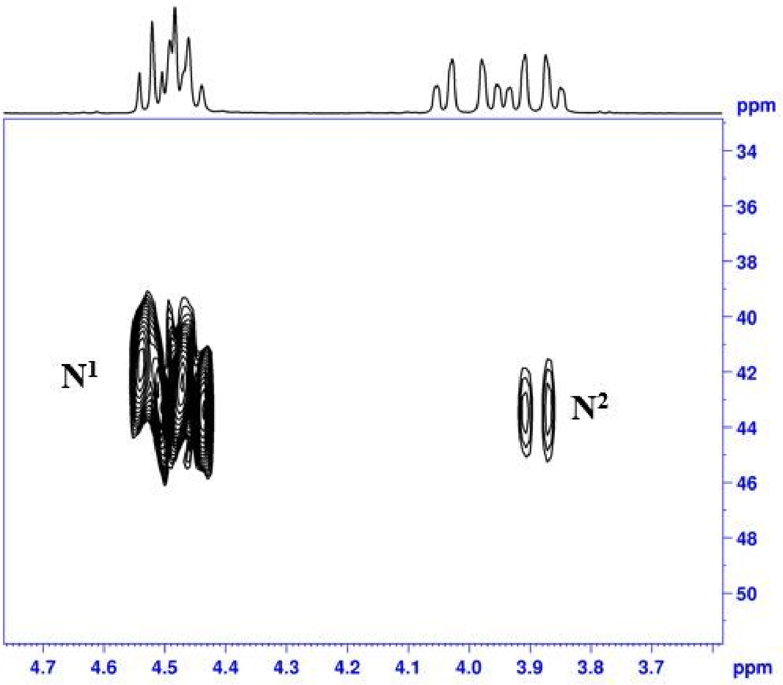

Figure S51. ^{1}H-^{15}N HMBC NMR (600.13 MHz, 60.81 MHz, 25ºC, DMSO-d_6) of **15**.

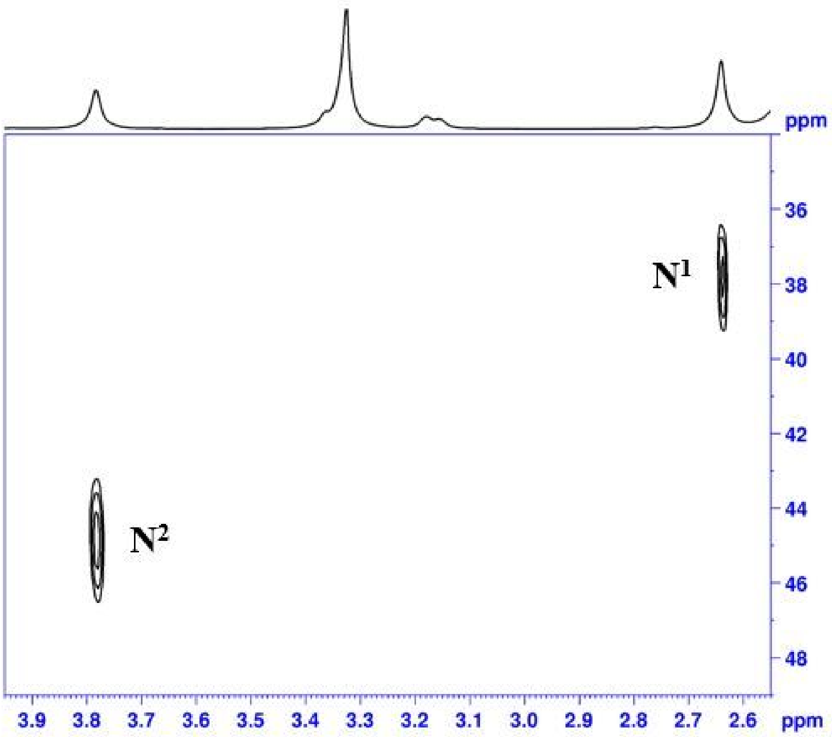

Figure S52. ^{1}H-^{15}N HMBC NMR (600.13 MHz, 60.81 MHz, 25ºC, DMSO-d_6) of **16**.

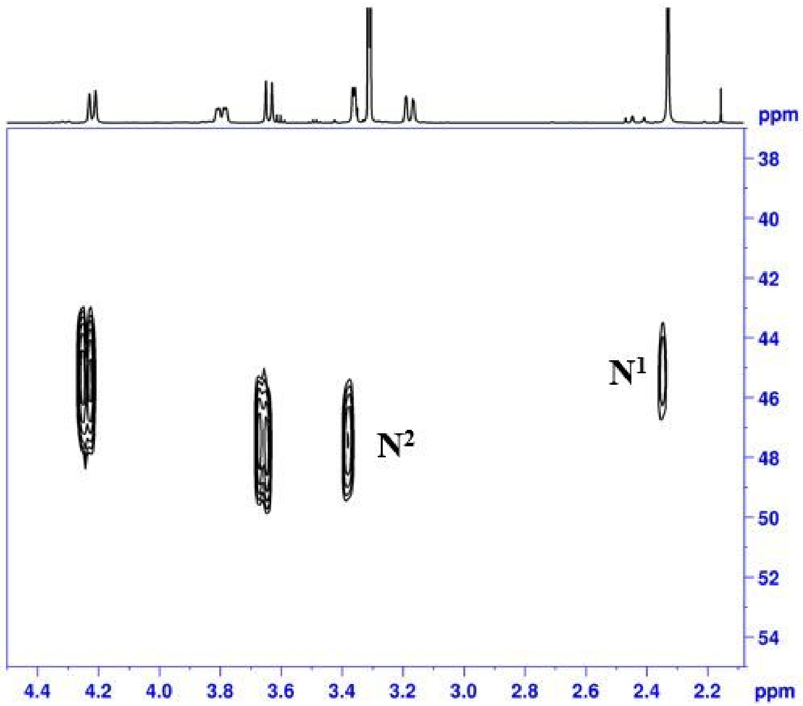

Figure S53. ^{1}H-^{15}N HMBC NMR (600.13 MHz, 60.81 MHz, 25ºC, CD_3OD) of **17**.

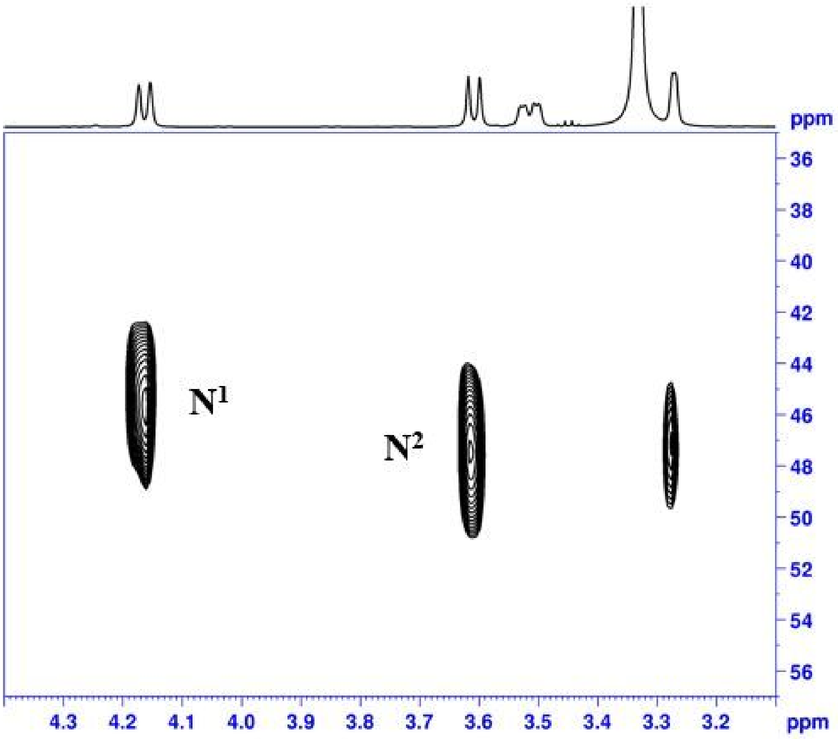

Figure S54. ^{1}H-^{15}N HMBC NMR (600.13 MHz, 60.81 MHz, 25ºC, DMSO-d_6) of **17**.

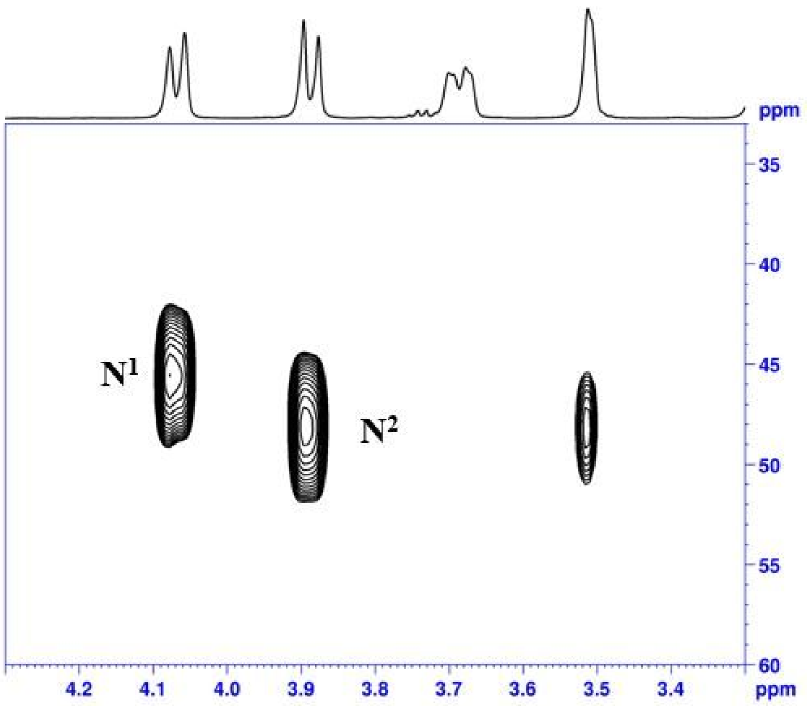

Figure S55. ^{1}H-^{15}N HMBC NMR (600.13 MHz, 60.81 MHz, 25ºC, $CDCl_3$) of **17**.

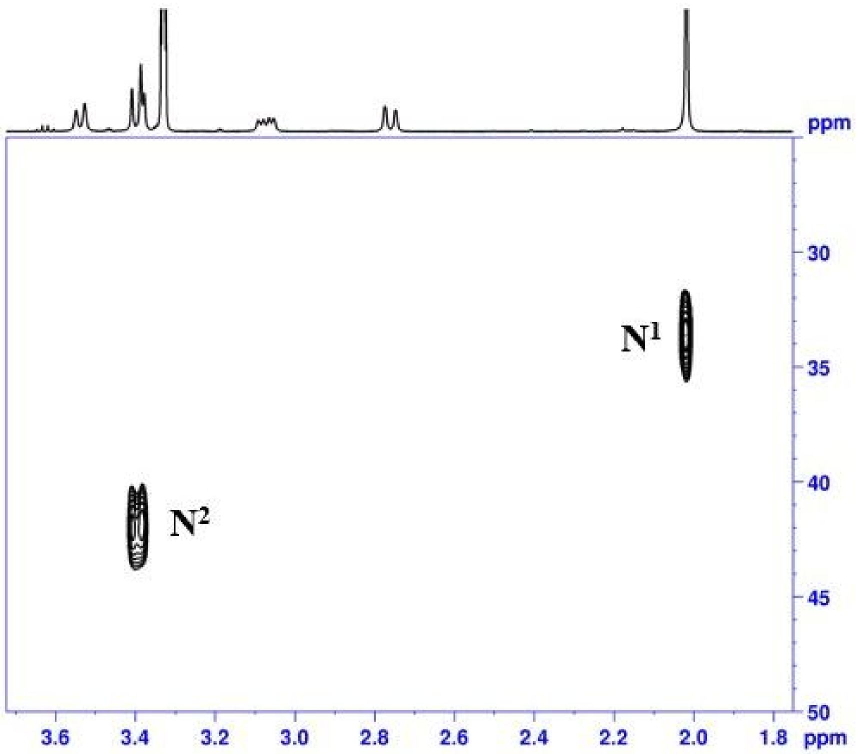

Figure S56. ^{1}H-^{15}N HMBC NMR (600.13 MHz, 60.81 MHz, 25ºC, CD_3OD) of **18**.

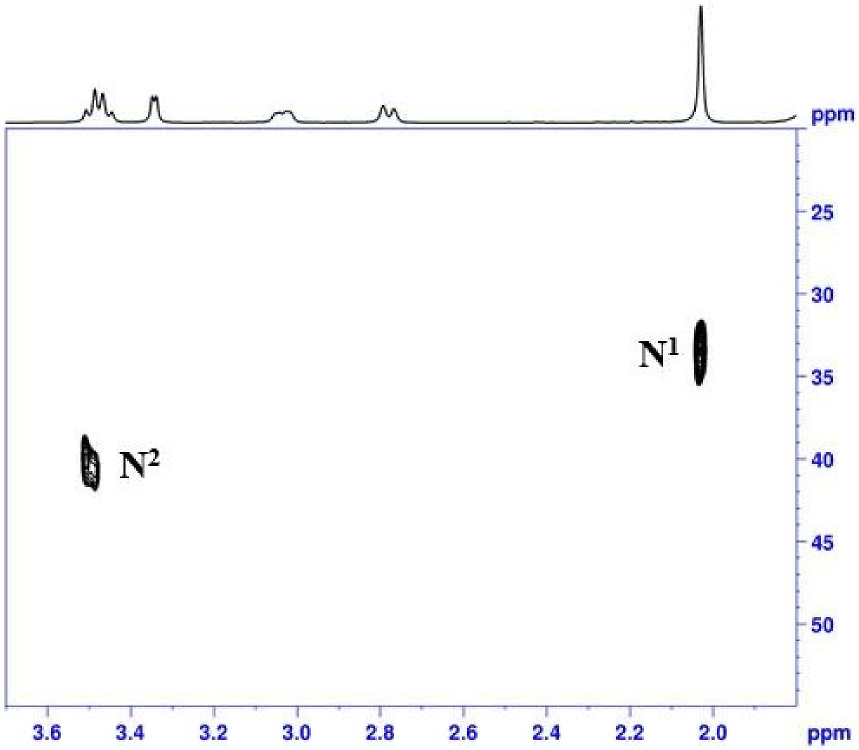

Figure S57. ^{1}H-^{15}N HMBC NMR (600.13 MHz, 60.81 MHz, 25ºC, $CDCl_3$) of **18**.

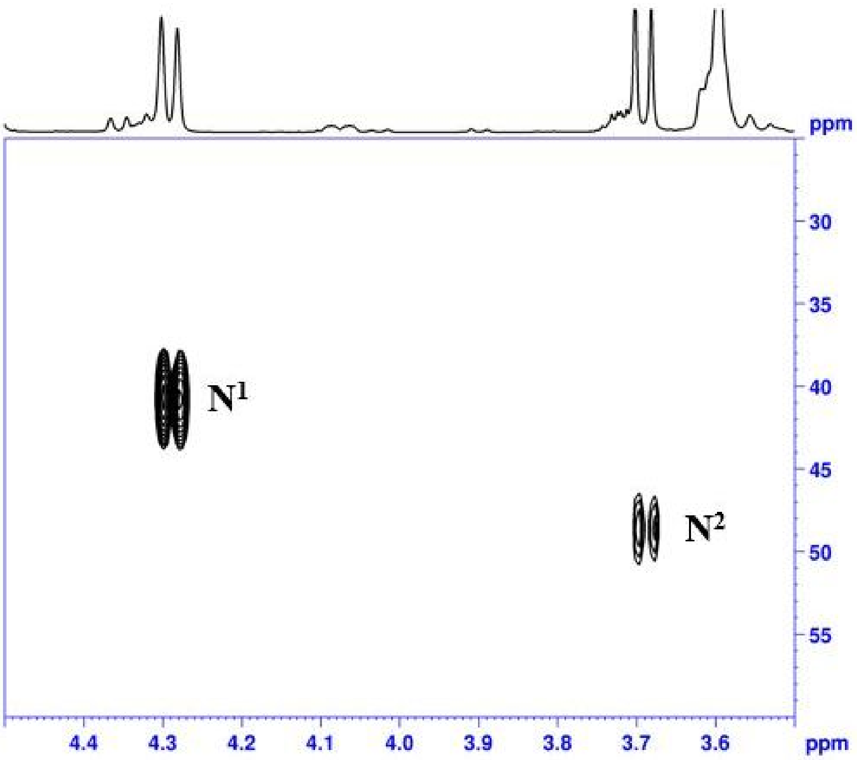

Figure S58. ^{1}H-^{15}N HMBC NMR (600.13 MHz, 60.81 MHz, 25ºC, $CDCl_3$) of **19**.

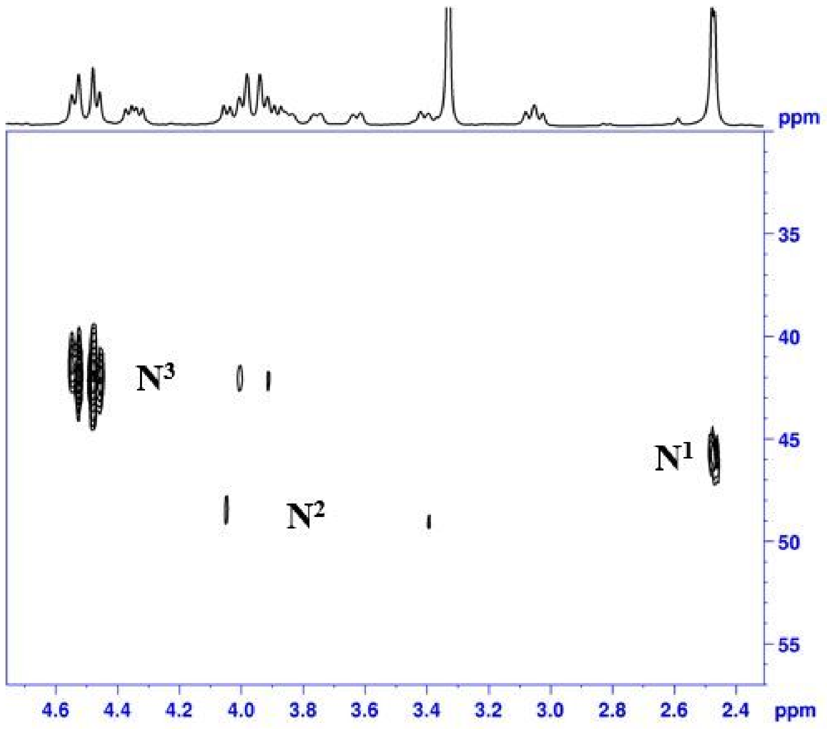

Figure S59. ^{1}H-^{15}N HMBC NMR (600.13 MHz, 60.81 MHz, 25ºC, CD_3OD) of **20**.

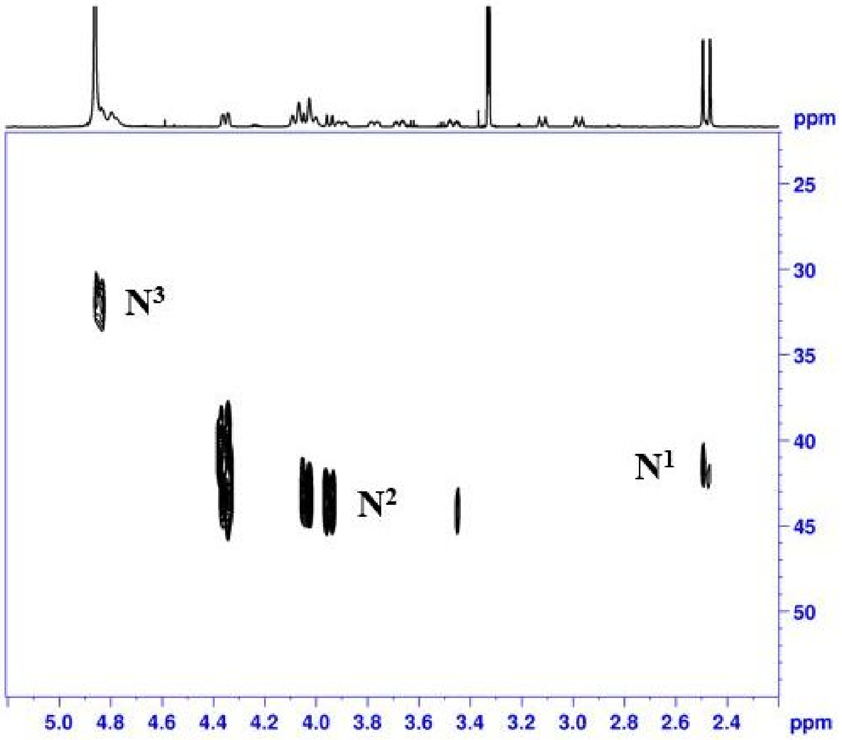

Figure S60. ^{1}H-^{15}N HMBC NMR (600.13 MHz, 60.81 MHz, 25ºC, CD_3OD) of **21**.

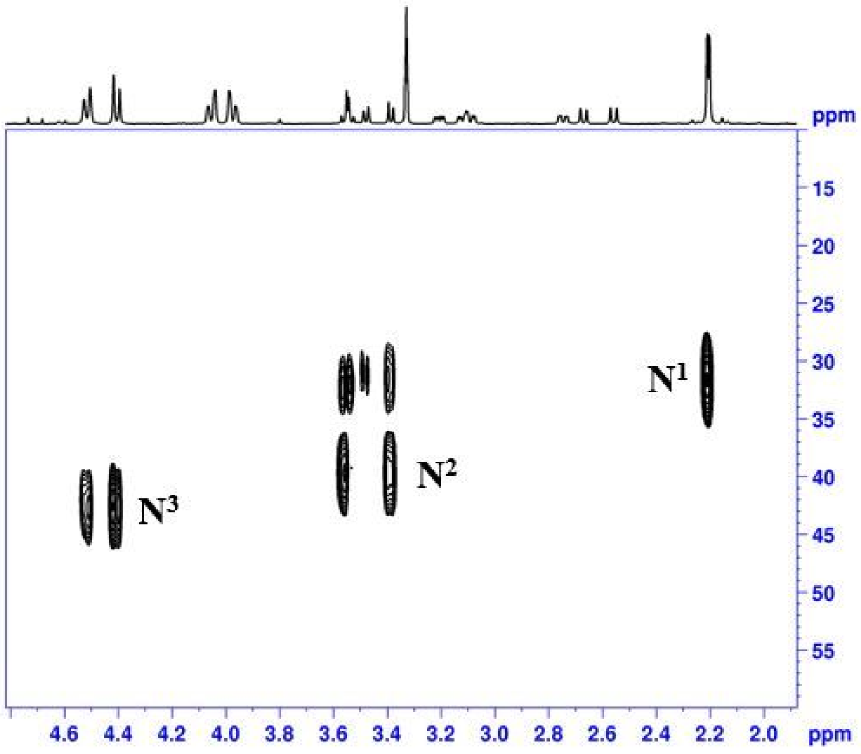

Figure S61. ^{1}H-^{15}N HMBC NMR (600.13 MHz, 60.81 MHz, 25ºC, CD_3OD) of **22**.

^{15}N assignments via ^{1}H-^{15}N NMR long range correlations

Table S1. ^{15}N chemical shifts for **1-8**.

Compound	Solvent	δ N (ppm)	
**1**	D_2O	24.6	
	DMSO-d_6	23.1	
	Acetone-d_6	24.1	
**2**	D_2O	42.9	
	DMSO-d_6	43.6	
	Acetone-d_6	44.2	
**3**	D_2O	64.3	
**4**	D_2O	24.7	
		δ N^1 (ppm)	δ N^2 (ppm)
**5**	D_2O	24.4	34.8
	DMSO-d_6	23.1	33.8
	Acetone-d_6	23.1	34.2
**6**	Acetone-d_6	41.2	47.1
**7**	D_2O	33.1	40.6
**8**	DMSO-d_6	109.1	31.0
	$CDCl_3$	107.9	31.3

Table S2. ^{15}N chemical shifts for **9-16**.

Complex	Solvent	δ N (ppm)	
**9**	D_2O	40.2	
**10**	D_2O	39.3	
**11**	D_2O	36.7	
		δ N^1 (ppm)	δ N^2 (ppm)
**12**	D_2O	41.1	42.2
**13**	D_2O	41.1	42.7
**14**	DMSO-d_6	41.4	43.5
**15**	D_2O	41.1	42.2
	DMSO-d_6	42.1	43.5
**16**	DMSO-d_6	37.7	44.7

Table S3. ^{15}N chemical shifts for **17-19**.

Complex	Solvent	δ N^1 (ppm)	δ N^2 (ppm)
**17**	CD_3OD	45.3	47.5
	DMSO-d_6	45.5	47.2
	$CDCl_3$	45.8	48.3
**18**	CD_3OD	33.7	42.1
	$CDCl_3$	33.9	40.9
**19**	$CDCl_3$	41.1	47.8

Table S4. ^{15}N chemical shifts for **20**, **21** and **22**.

Complex	Solvent	δ N^1 (ppm)	δ N^2 (ppm)	δ N^3 (ppm)
**20**	CD_3OD	46.1	48.5	41.9
**21**	CD_3OD	41.5	44.1	31.9
**22**	CD_3OD	31.7	39.7	42.7

Single crystal X-ray diffraction tables

Table S5. Crystal data and structure refinement for **13** and **14**.

	**13**	**14**
Empirical formula	$C_{39}H_{70}Cl_3N_{13}O_2P_4Ru_2S_2Zn$	$C_{35}H_{62}Cl_5N_{13}O_2P_4Ru_2Zn_2$
Formula weight	1314.94	1330.98
Temperature/K	100.0	273.15
Crystal system	monoclinic	triclinic
Space group	P21/n	P-1
a/Å	11.7429(4)	9.0384(12)
b/Å	20.0365(6)	11.3474(15)
c/Å	22.2458(8)	12.7233(17)
α/°	90	76.924(2)
β/°	97.3850(10)	75.018(2)
γ/°	90	88.294(2)
Volume/Å^3	5190.7(3)	1227.3(3)
Z	4	1
ρ_{calc}g/cm^3	1.683	1.801
μ/mm^{-1}	1.440	2.020
F(000)	2688.0	672.0
Crystal size/mm^3	0.2 × 0.1 × 0.01	0.2 × 0.15 × 0.1
Radiation	MoKα (λ = 0.71073)	MoKα (λ = 0.71073)
2Θ range for data collection/°	3.74 to 48.814	3.402 to 52.742
Index ranges	-13 ≤ h ≤ 13, -23 ≤ k ≤ 23, -25 ≤ l ≤ 25	-11 ≤ h ≤ 11, -9 ≤ k ≤ 14, -15 ≤ l ≤ 15
Reflections collected	68339	7500
Independent reflections	8537 [Rint = 0.0759, Rsigma = 0.0416]	4931 [Rint = 0.0237, Rsigma = 0.0494]
Data/restraints/parameters	8537/671/614	4931/0/290
Goodness-of-fit on F^2	1.237	1.031
Final R indexes [I>=2σ (I)]	R1 = 0.0756, wR2 = 0.1402	R1 = 0.0395, wR2 = 0.0906
Final R indexes [all data]	R1 = 0.0965, wR2 = 0.1532	R1 = 0.0480, wR2 = 0.0963
Largest diff. peak/hole /e Å^{-3}	1.39/-0.91	1.06/-0.57

Table S6. Crystal data and structure refinement for **18**, **21** and **22**.

	**18**	**21**	**22**
Empirical formula	$C_{50}H_{52}Cl_3F_3N_3O_3P_3RuS$	$C_{39}H_{56}F_9N_6O_{12}P_3RuS_3$	$C_{38}H_{52}F_3N_6O_4P_3RuS$
Formula weight	1132.33	1262.05	939.89
Temperature/K	100.0	100.0	100.0
Crystal system	monoclinic	triclinic	triclinic
Space group	$P2_1/c$	P-1	P-1
a/Å	10.9792(2)	12.2349(14)	11.2460(4)
b/Å	31.2112(7)	13.2752(15)	13.0913(4)
c/Å	14.3403(3)	16.7049(19)	15.3552(5)
α/°	90	86.554(6)	88.848(2)
β/°	94.0980(10)	84.823(6)	75.828(2)
γ/°	90	70.111(6)	69.046(2)
Volume/$Å^3$	4901.48(17)	2539.7(5)	2041.22(12)
Z	4	2	2
$\rho_{calc}g/cm^3$	1.534	1.650	1.529
μ/mm^{-1}	5.899	5.415	5.223
F(000)	2320.0	1292.0	972.0
Crystal size/mm^3	0.019 × 0.015 × 0.012	0.08 × 0.05 × 0.03	0.07 × 0.05 × 0.02
Radiation	CuKα (λ = 1.54178)	CuKα (λ = 1.54178)	CuKα (λ = 1.54178)
2Θ range for data collection/°	5.662 to 144.83	5.314 to 145.106	5.952 to 140.1
Index ranges	$-13 \le h \le 12$, $-38 \le k \le 38$, $-17 \le l \le 17$	$-15 \le h \le 15$, $-16 \le k \le 16$, $-20 \le l \le 20$	$-13 \le h \le 13$, $-15 \le k \le 15$, $-18 \le l \le 18$
Reflections collected	64995	52621	42309
Independent reflections	9681 [R_{int} = 0.0514, R_{sigma} = 0.0293]	9890 [R_{int} = 0.0346, R_{sigma} = 0.0222]	7667 [R_{int} = 0.0354, R_{sigma} = 0.0226]
Data/restraints/parameters	9681/0/606	9890/770/707	7667/12/531
Goodness-of-fit on F^2	1.146	1.045	1.076
Final R indexes [I>=2σ (I)]	R_1 = 0.0449 wR_2 = 0.0884	R_1 = 0.0681 wR_2 = 0.1503	R_1 = 0.0392 wR_2 = 0.0841
Final R indexes [all data]	R_1 = 0.0559 wR_2 = 0.0937	R_1 = 0.0784 wR_2 = 0.1592	R_1 = 0.0459 wR_2 = 0.0886
Largest diff. peak/hole / e $Å^{-3}$	1.00/-0.67	1.29/-1.21	1.09/-1.06

Table S7. Bond Lengths for **13**.

Atom	Atom	Length/Å	Atom	Atom	Length/Å
Ru1	P1	2.256(2)	N5	C8	1.478(13)
Ru1	P2	2.277(2)	N5	C11	1.481(14)
Ru1	C13	2.239(9)	N5	C12	1.467(17)
Ru1	C14	2.233(9)	N6	C7	1.479(12)
Ru1	C15	2.202(9)	N6	C10	1.464(12)
Ru1	C16	2.219(10)	N6	C12	1.426(15)
Ru1	C17	2.238(9)	N7	C25	1.458(11)
Ru1	NCNA	2.021(8)	N7	C28	1.473(11)
Ru1	CCNB	2.021(8)	N7	C29	1.480(11)
Ru2	P3	2.264(2)	N8	C24	1.476(11)
Ru2	P4	2.272(2)	N8	C27	1.493(11)
Ru2	C30	2.235(9)	N8	C28	1.440(11)
Ru2	C31	2.240(9)	N9	C26	1.472(10)
Ru2	C32	2.211(9)	N9	C27	1.457(11)
Ru2	C33	2.211(10)	N9	C29	1.453(11)
Ru2	C34	2.236(9)	N10	C18	1.486(12)
Ru2	CCNA	2.023(8)	N10	C21	1.471(13)
Ru2	NCNB	2.023(8)	N10	C23	1.475(12)
Zn1	Cl1	2.235(3)	N11	C19	1.479(12)
Zn1	Cl2	2.246(2)	N11	C21	1.460(15)
Zn1	Cl3	2.251(3)	N11	C22	1.474(13)
Zn1	N1	2.123(7)	N12	C20	1.467(11)
P1	C1	1.832(8)	N12	C22	1.473(11)
P1	C2	1.841(9)	N12	C23	1.457(12)
P1	C3	1.845(9)	C13	C14	1.407(13)

P2	C7	1.837(9)	C13	C17	1.397(13)
P2	C8	1.839(10)	C14	C15	1.446(14)
P2	C9	1.843(9)	C15	C16	1.414(14)
P3	C24	1.840(9)	C16	C17	1.434(14)
P3	C25	1.851(9)	C30	C31	1.408(13)
P3	C26	1.835(8)	C30	C34	1.428(14)
P4	C18	1.835(10)	C31	C32	1.439(13)
P4	C19	1.851(9)	C32	C33	1.404(13)
P4	C20	1.840(8)	C33	C34	1.432(13)
N1	C1	1.520(9)	NCNA	CCNA	1.156(11)
N1	C4	1.493(11)	NCNB	CCNB	1.156(11)
N1	C5	1.494(11)	S2DB	O2DB	1.695(16)
N2	C2	1.474(11)	S2DB	C3DB	1.755(17)
N2	C4	1.453(11)	S2DB	C4DB	1.747(16)
N2	C6	1.449(12)	S2DA	O2DA	1.55(3)
N3	C3	1.483(11)	S2DA	C3DA	1.761(16)
N3	C5	1.450(11)	S2DA	C4DA	1.762(16)
N3	C6	1.472(12)	S1D	O1D	1.492(9)
N4	C9	1.463(12)	S1D	C1D	1.740(11)
N4	C10	1.462(11)	S1D	C2D	1.776(12)
N4	C11	1.453(13)			

Table S8. Bond Lengths for **14**.

Atom	Atom	Length/Å	Atom	Atom	Length/Å
Ru1	P1	2.2515(10)	N5	C11	1.454(6)
Ru1	P2	2.2477(11)	N4	C7	1.471(5)
Ru1	C13	2.243(4)	N4	C10	1.459(6)
Ru1	C14	2.238(4)	N4	C12	1.463(6)
Ru1	C15	2.208(4)	N6	C9	1.482(5)
Ru1	C16	2.203(4)	N6	C11	1.480(6)
Ru1	C17	2.235(4)	N6	C12	1.484(6)
Ru1	NCN1	2.027(4)	N1	C1	1.497(5)
Ru1	CCN1	2.027(4)	N1	C4	1.501(5)
Zn1	Cl1	2.2455(12)	N1	C5	1.491(5)
Zn1	Cl2	2.2380(11)	N2	C2	1.480(5)
Zn1	N1	2.114(3)	N2	C5	1.457(5)
Zn1	O1	1.940(3)	N2	C6	1.478(5)
P1	C1	1.842(4)	N3	C3	1.479(5)
P1	C2	1.835(4)	N3	C4	1.448(5)
P1	C3	1.830(4)	N3	C6	1.461(6)
P2	C7	1.846(4)	C13	C14	1.415(6)
P2	C8	1.851(4)	C13	C17	1.409(6)
P2	C9	1.844(4)	C14	C15	1.425(6)
N5	C8	1.473(5)	C15	C16	1.394(6)
N5	C10	1.463(6)	C16	C17	1.431(6)

Table S9. Bond Lengths for **18**.

Atom	Atom	Length/Å	Atom	Atom	Length/Å
Ru1	P1	2.3187(9)	C20	C21	1.386(5)
Ru1	P2	2.3541(8)	C21	C22	1.390(5)
Ru1	P3	2.3564(9)	C22	C23	1.388(5)
Ru1	C8	2.229(3)	C23	C24	1.383(5)
Ru1	C9	2.239(3)	C25	C26	1.390(5)

Ru1	C10	2.259(3)	C25	C30	1.396(5)
Ru1	C11	2.257(4)	C26	C27	1.389(5)
Ru1	C12	2.241(3)	C27	C28	1.389(5)
P1	C3	1.857(3)	C28	C29	1.387(6)
P1	C1	1.862(4)	C29	C30	1.391(5)
P1	C2	1.857(3)	C31	C32	1.394(5)
P2	C13	1.828(4)	C31	C36	1.396(5)
P2	C19	1.856(3)	C32	C33	1.396(5)
P2	C25	1.840(3)	C33	C34	1.385(5)
P3	C31	1.840(3)	C34	C35	1.378(6)
P3	C37	1.851(3)	C35	C36	1.386(5)
P3	C43	1.846(3)	C37	C38	1.389(5)
N1	C1	1.464(4)	C37	C42	1.403(5)
N1	C4	1.455(4)	C38	C39	1.398(5)
N1	C6	1.464(5)	C39	C40	1.382(6)
N2	C2	1.460(4)	C40	C41	1.388(5)
N2	C5	1.471(5)	C41	C42	1.391(5)
N2	C7	1.470(4)	C43	C44	1.404(5)
N3	C3	1.454(4)	C43	C48	1.397(5)
N3	C4	1.469(5)	C44	C45	1.393(5)
N3	C5	1.452(4)	C45	C46	1.388(5)
C8	C9	1.426(5)	C46	C47	1.384(5)
C8	C12	1.424(5)	C47	C48	1.392(5)
C9	C10	1.420(5)	S1TF	O1TF	1.440(3)
C10	C11	1.426(5)	S1TF	O2TF	1.448(3)
C11	C12	1.427(5)	S1TF	O3TF	1.435(3)
C13	C14	1.396(5)	S1TF	C1TF	1.815(5)
C13	C18	1.403(5)	F1TF	C1TF	1.342(5)
C14	C15	1.387(5)	F2TF	C1TF	1.330(5)
C15	C16	1.390(5)	F3TF	C1TF	1.335(5)
C16	C17	1.386(5)	Cl1H	C1H	1.739(4)
C17	C18	1.390(5)	Cl2H	C1H	1.771(4)
C19	C20	1.392(5)	Cl3H	C1H	1.760(5)
C19	C24	1.399(5)			

Table S10. Bond Lengths for **21**.

Atom	Atom	Length/Å	Atom	Atom	Length/Å
Ru1	P1	2.2821(14)	C16	C17	1.368(9)
Ru1	P2	2.2838(13)	C17	C18	1.377(9)
Ru1	P3	2.3433(12)	C18	C19	1.396(8)
Ru1	C33	2.236(6)	C21	C22	1.395(8)
Ru1	C34	2.231(5)	C21	C26	1.398(8)
Ru1	C35	2.245(5)	C22	C23	1.376(9)
Ru1	C36	2.234(5)	C23	C24	1.390(9)
Ru1	C37	2.239(5)	C24	C25	1.387(7)
P1	C2	1.861(6)	C25	C26	1.407(7)
P1	C3	1.842(6)	C27	C28	1.398(7)
P1	C1	1.836(6)	C27	C32	1.393(7)
P2	C8	1.859(6)	C28	C29	1.393(8)
P2	C9	1.841(6)	C29	C30	1.373(9)
P2	C10	1.848(6)	C30	C31	1.385(8)
P3	C14	1.843(5)	C31	C32	1.389(8)
P3	C26	1.847(5)	C33	C34	1.410(8)
P3	C27	1.834(5)	C33	C37	1.423(9)
S2TA	O4TA	1.494(5)	C34	C35	1.412(8)
S2TB	O4TB	1.494(5)	C35	C36	1.443(7)
N1	C1	1.502(7)	C36	C37	1.417(9)
N1	C5	1.538(8)	S2TA	O5TA	1.501(5)

N1	C7	1.528(8)	S2TA	O6TA	1.490(5)
N2	C2	1.470(7)	S2TB	O5TB	1.491(5)
N2	C4	1.449(8)	S2TB	O6TB	1.490(5)
N2	C5	1.435(8)	S3TB	O7TA	1.437(7)
N3	C3	1.468(7)	S3TB	O8TA	1.432(7)
N3	C4	1.464(8)	S3TB	O9TA	1.421(7)
N3	C6	1.477(8)	S3TB	C3TA	1.790(9)
N4	C8	1.471(7)	F7TA	C3TA	1.319(10)
N4	C11	1.468(8)	F8TA	C3TA	1.311(10)
N4	C12	1.436(9)	F9TA	C3TA	1.365(11)
N5	C10	1.469(7)	S3TA	O7TB	1.466(13)
N5	C11	1.463(7)	S3TA	O8TB	1.352(13)
N5	C13	1.413(9)	S3TA	O9TB	1.482(12)
N6	C9	1.508(7)	S3TA	C3TB	1.806(14)
N6	C12	1.527(8)	F7TB	C3TB	1.324(16)
N6	C13	1.550(9)	F8TB	C3TB	1.308(16)
C2T	S2TA	1.838(11)	F9TB	C3TB	1.345(16)
C2T	F4TA	1.334(10)	S1T	O1T	1.423(5)
C2T	F5TA	1.256(9)	S1T	C1T	1.801(7)
C2T	F6TA	1.336(10)	S1T	O2TB	1.381(7)
C2T	S2TB	1.872(9)	S1T	O3TB	1.539(7)
C2T	F4TB	1.354(13)	S1T	O2TA	1.343(9)
C2T	F5TB	1.292(13)	S1T	O3TA	1.576(10)
C2T	F6TB	1.266(13)	F1T	C1T	1.325(8)
C14	C15	1.396(7)	F2T	C1T	1.327(8)
C14	C19	1.400(8)	F3T	C1T	1.332(8)
C15	C16	1.387(8)			

Table S11. Bond Lengths for **22**.

Atom	Atom	Length/Å	Atom	Atom	Length/Å
Ru1	P1	2.3066(8)	C15	C16	1.379(5)
Ru1	P2	2.3009(8)	C16	C17	1.389(5)
Ru1	P3	2.3116(8)	C17	C18	1.390(5)
Ru1	C32	2.251(3)	C18	C19	1.397(5)
Ru1	C33	2.247(3)	C20	C21	1.402(4)
Ru1	C34	2.231(3)	C20	C25	1.397(5)
Ru1	C35	2.239(3)	C21	C22	1.384(5)
Ru1	C36	2.251(3)	C22	C23	1.382(5)
P1	C1	1.862(3)	C23	C24	1.392(5)
P1	C2	1.839(3)	C24	C25	1.383(5)
P1	C3	1.846(3)	C26	C27	1.396(5)
P2	C8	1.855(3)	C26	C31	1.395(5)
P2	C9	1.861(4)	C27	C28	1.385(5)
P2	C10	1.859(3)	C28	C29	1.385(5)
P3	C14	1.833(3)	C29	C30	1.389(5)
P3	C20	1.844(3)	C30	C31	1.386(5)
P3	C26	1.837(3)	C32	C33	1.443(5)
N1	C1	1.456(4)	C32	C36	1.406(5)
N1	C4	1.465(4)	C33	C34	1.412(5)
N1	C6	1.468(4)	C34	C35	1.419(5)
N2	C2	1.472(4)	C35	C36	1.427(5)
N2	C5	1.464(4)	F1TA	C1TA	1.318(6)
N2	C7	1.466(4)	F2TA	C1TA	1.335(5)
N3	C3	1.467(4)	F3TA	C1TA	1.339(5)
N3	C4	1.468(4)	S1TA	O1TA	1.438(3)
N3	C5	1.455(4)	S1TA	O2TA	1.460(3)

N4	C9	1.479(4)	S1TA	O3TA	1.439(3)
N4	C11	1.468(5)	F2TA	C1TA	1.335(5)
N4	C12	1.474(5)	F3TA	C1TA	1.339(5)
N5	C10	1.480(4)	S1TB	O3TB	1.416(17)
N5	C11	1.468(5)	S1TB	O1TB	1.447(18)
N5	C13	1.470(5)	S1TB	O2TB	1.477(18)
N6	C8	1.478(4)	S1TB	C1TB	1.92(2)
N6	C12	1.461(5)	F2TB	C1TB	1.340(19)
N6	C13	1.479(5)	F3TB	C1TB	1.328(19)
C14	C15	1.403(5)			
C14	C19	1.389(5)			

Table S12. Bond Angles for **13**.

**Atom**	**Atom**	**Atom**	**Angle/°**	**Atom**	**Atom**	**Atom**	**Angle/°**
P1	Ru1	P2	95.60(8)	C4	N1	C5	108.2(6)
C13	Ru1	P1	133.2(2)	C5	N1	Zn1	111.7(5)
C13	Ru1	P2	131.2(2)	C5	N1	C1	110.3(7)
C14	Ru1	P1	99.9(3)	C4	N2	C2	112.7(7)
C14	Ru1	P2	156.1(3)	C6	N2	C2	110.8(7)
C14	Ru1	C13	36.7(3)	C6	N2	C4	108.1(7)
C14	Ru1	C17	62.1(4)	C5	N3	C3	111.1(7)
C15	Ru1	P1	95.4(3)	C5	N3	C6	108.0(7)
C15	Ru1	P2	122.6(3)	C6	N3	C3	110.9(7)
C15	Ru1	C13	61.8(4)	C10	N4	C9	111.9(8)
C15	Ru1	C14	38.1(3)	C11	N4	C9	112.6(8)
C15	Ru1	C16	37.3(4)	C11	N4	C10	108.0(8)
C15	Ru1	C17	62.6(4)	C8	N5	C11	109.8(10)
C16	Ru1	P1	124.4(3)	C12	N5	C8	112.5(9)
C16	Ru1	P2	93.5(3)	C12	N5	C11	107.7(8)
C16	Ru1	C13	61.4(4)	C10	N6	C7	111.0(8)
C16	Ru1	C14	62.7(4)	C12	N6	C7	111.5(9)
C16	Ru1	C17	37.5(4)	C12	N6	C10	108.0(8)
C17	Ru1	P1	157.9(3)	C25	N7	C28	109.6(7)
C17	Ru1	P2	98.0(3)	C25	N7	C29	110.3(6)
C17	Ru1	C13	36.4(3)	C28	N7	C29	107.2(7)
NCNA	Ru1	P1	88.8(2)	C24	N8	C27	109.9(6)
NCNA	Ru1	P2	86.5(2)	C28	N8	C24	111.6(7)
NCNA	Ru1	C13	93.9(3)	C28	N8	C27	106.6(7)
NCNA	Ru1	C14	111.8(3)	C27	N9	C26	111.3(6)
NCNA	Ru1	C15	149.8(3)	C29	N9	C26	112.0(7)
NCNA	Ru1	C16	146.5(3)	C29	N9	C27	108.0(7)
NCNA	Ru1	C17	109.3(3)	C21	N10	C18	111.3(8)
CCNB	Ru1	P1	88.8(2)	C21	N10	C23	107.7(8)
CCNB	Ru1	P2	86.5(2)	C23	N10	C18	110.5(7)
CCNB	Ru1	C13	93.9(3)	C21	N11	C19	110.0(8)
CCNB	Ru1	C14	111.8(3)	C21	N11	C22	109.1(8)
CCNB	Ru1	C15	149.8(3)	C22	N11	C19	111.4(8)
CCNB	Ru1	C16	146.5(3)	C20	N12	C22	110.7(7)
CCNB	Ru1	C17	109.3(3)	C23	N12	C20	111.6(7)
P3	Ru2	P4	97.60(8)	C23	N12	C22	108.4(8)
C30	Ru2	P3	132.5(3)	N1	C1	P1	112.3(5)
C30	Ru2	P4	129.9(3)	N2	C2	P1	113.3(6)
C30	Ru2	C31	36.7(3)	N3	C3	P1	113.0(6)
C30	Ru2	C34	37.3(3)	N2	C4	N1	113.2(7)
C31	Ru2	P3	156.5(3)	N3	C5	N1	114.3(7)
C31	Ru2	P4	97.2(2)	N2	C6	N3	115.2(7)
C32	Ru2	P3	122.8(2)	N6	C7	P2	113.3(7)
C32	Ru2	P4	94.5(2)	N5	C8	P2	112.6(7)
C32	Ru2	C30	61.8(3)	N4	C9	P2	112.1(7)

C32	Ru2	C31	37.7(3)	N4	C10	N6	113.7(7)
C32	Ru2	C33	37.0(3)	N4	C11	N5	113.5(8)
C32	Ru2	C34	62.7(3)	N6	C12	N5	115.0(9)
C33	Ru2	P3	94.3(2)	C14	C13	Ru1	71.4(5)
C33	Ru2	P4	124.5(2)	C17	C13	Ru1	71.8(6)
C33	Ru2	C30	61.6(4)	C17	C13	C14	110.8(9)
C33	Ru2	C31	62.3(3)	C13	C14	Ru1	71.9(6)
C33	Ru2	C34	37.6(3)	C13	C14	C15	106.1(8)
C34	Ru2	P3	98.4(2)	C15	C14	Ru1	69.8(5)
C34	Ru2	P4	156.9(2)	C14	C15	Ru1	72.1(5)
C34	Ru2	C31	62.7(3)	C16	C15	Ru1	72.0(6)
CCNA	Ru2	P3	86.7(2)	C16	C15	C14	108.1(9)
CCNA	Ru2	P4	89.0(2)	C15	C16	Ru1	70.7(5)
CCNA	Ru2	C30	93.0(3)	C15	C16	C17	108.0(9)
CCNA	Ru2	C31	111.6(3)	C17	C16	Ru1	71.9(5)
CCNA	Ru2	C32	149.4(3)	C13	C17	Ru1	71.9(5)
CCNA	Ru2	C33	145.9(3)	C13	C17	C16	107.0(9)
CCNA	Ru2	C34	108.5(3)	C16	C17	Ru1	70.5(5)
NCNB	Ru2	P3	86.7(2)	N10	C18	P4	112.7(7)
NCNB	Ru2	P4	89.0(2)	N11	C19	P4	112.5(6)
NCNB	Ru2	C30	93.0(3)	N12	C20	P4	112.9(6)
NCNB	Ru2	C31	111.6(3)	N11	C21	N10	114.6(8)
NCNB	Ru2	C32	149.4(3)	N12	C22	N11	113.8(8)
NCNB	Ru2	C33	145.9(3)	N12	C23	N10	114.9(8)
NCNB	Ru2	C34	108.5(3)	N8	C24	P3	113.1(6)
Cl1	Zn1	Cl2	115.51(10)	N7	C25	P3	114.8(6)
Cl1	Zn1	Cl3	117.91(10)	N9	C26	P3	112.9(6)
Cl2	Zn1	Cl3	112.85(10)	N9	C27	N8	115.2(7)
N1	Zn1	Cl1	99.34(19)	N8	C28	N7	117.0(7)
N1	Zn1	Cl2	104.60(19)	N9	C29	N7	114.4(7)
N1	Zn1	Cl3	103.8(2)	C31	C30	Ru2	71.9(5)
C1	P1	Ru1	117.5(3)	C31	C30	C34	110.3(9)
C1	P1	C2	97.8(4)	C34	C30	Ru2	71.4(5)
C1	P1	C3	98.5(4)	C30	C31	Ru2	71.5(5)
C2	P1	Ru1	124.1(3)	C30	C31	C32	106.6(8)
C2	P1	C3	96.8(4)	C32	C31	Ru2	70.0(5)
C3	P1	Ru1	117.1(3)	C31	C32	Ru2	72.2(5)
C7	P2	Ru1	112.7(3)	C33	C32	Ru2	71.5(5)
C7	P2	C8	97.5(5)	C33	C32	C31	108.1(8)
C7	P2	C9	96.8(4)	C32	C33	Ru2	71.5(5)
C8	P2	Ru1	121.0(3)	C32	C33	C34	109.4(8)
C8	P2	C9	98.0(5)	C34	C33	Ru2	72.1(5)
C9	P2	Ru1	125.2(3)	C30	C34	Ru2	71.3(5)
C24	P3	Ru2	120.9(3)	C30	C34	C33	105.6(8)
C24	P3	C25	97.1(4)	C33	C34	Ru2	70.3(5)
C25	P3	Ru2	114.3(3)	CCNA	NCNA	Ru1	172.9(7)
C26	P3	Ru2	124.3(3)	NCNA	CCNA	Ru2	173.3(7)
C26	P3	C24	98.3(4)	CCNB	NCNB	Ru2	173.3(7)
C26	P3	C25	96.2(4)	NCNB	CCNB	Ru1	172.9(7)
C18	P4	Ru2	121.7(3)	O2DB	S2DB	C3DB	100.1(14)
C18	P4	C19	97.4(5)	O2DB	S2DB	C4DB	97.1(15)
C18	P4	C20	97.8(4)	C4DB	S2DB	C3DB	100.5(16)
C19	P4	Ru2	114.0(3)	O2DA	S2DA	C3DA	102.6(15)
C20	P4	Ru2	122.8(3)	O2DA	S2DA	C4DA	111.5(16)
C20	P4	C19	98.1(4)	C3DA	S2DA	C4DA	93.7(16)
C1	N1	Zn1	108.4(5)	O1D	S1D	C1D	104.8(6)
C4	N1	Zn1	107.2(5)	O1D	S1D	C2D	104.4(6)
C4	N1	C1	110.9(6)	C1D	S1D	C2D	98.8(7)

Table S13. Bond Angles for **14**.

Atom	Atom	Atom	Angle/°	Atom	Atom	Atom	Angle/°
P2	Ru1	P1	96.89(4)	C9	P2	C7	97.8(2)
C13	Ru1	P1	126.35(12)	C9	P2	C8	98.6(2)
C13	Ru1	P2	136.71(12)	C10	N5	C8	112.0(3)
C14	Ru1	P1	157.21(11)	C11	N5	C8	111.3(3)
C14	Ru1	P2	101.19(11)	C11	N5	C10	108.8(4)
C14	Ru1	C13	36.82(15)	C10	N4	C7	110.8(3)
C15	Ru1	P1	129.39(12)	C10	N4	C12	108.3(3)
C15	Ru1	P2	90.90(11)	C12	N4	C7	110.7(3)
C15	Ru1	C13	61.75(15)	C9	N6	C12	110.1(3)
C15	Ru1	C14	37.37(15)	C11	N6	C9	110.3(3)
C15	Ru1	C17	61.97(16)	C11	N6	C12	108.3(3)
C16	Ru1	P1	97.49(11)	C1	N1	Zn1	109.1(2)
C16	Ru1	P2	115.92(12)	C1	N1	C4	110.2(2)
C16	Ru1	C13	61.94(15)	C4	N1	Zn1	109.1(2)
C16	Ru1	C14	62.09(15)	C5	N1	Zn1	110.2(2)
C16	Ru1	C15	36.84(15)	C5	N1	C1	110.8(3)
C16	Ru1	C17	37.63(15)	C5	N1	C4	107.6(3)
C17	Ru1	P1	96.08(11)	C5	N2	C2	110.8(3)
C17	Ru1	P2	152.12(12)	C5	N2	C6	109.3(3)
C17	Ru1	C13	36.68(15)	C6	N2	C2	111.0(3)
C17	Ru1	C14	61.77(15)	C4	N3	C3	111.6(3)
NCN1	Ru1	P1	85.50(11)	C4	N3	C6	109.0(3)
NCN1	Ru1	P2	88.69(11)	C6	N3	C3	111.3(3)
NCN1	Ru1	C13	95.87(15)	N1	C1	P1	113.3(3)
NCN1	Ru1	C14	108.52(15)	N2	C2	P1	113.4(3)
NCN1	Ru1	C15	144.81(15)	N3	C3	P1	113.2(3)
NCN1	Ru1	C16	154.45(15)	N3	C4	N1	114.0(3)
NCN1	Ru1	C17	116.89(15)	N2	C5	N1	113.8(3)
CCN1	Ru1	P1	85.50(11)	N3	C6	N2	113.6(3)
CCN1	Ru1	P2	88.69(11)	N4	C7	P2	113.5(3)
CCN1	Ru1	C13	95.87(15)	N5	C8	P2	111.5(3)
CCN1	Ru1	C14	108.52(15)	N6	C9	P2	112.8(3)
CCN1	Ru1	C15	144.81(15)	N4	C10	N5	114.3(3)
CCN1	Ru1	C16	154.45(15)	N5	C11	N6	114.4(3)
CCN1	Ru1	C17	116.89(15)	N4	C12	N6	114.9(3)
Cl2	Zn1	Cl1	115.91(4)	C14	C13	Ru1	71.4(2)
N1	Zn1	Cl1	104.93(9)	C17	C13	Ru1	71.4(2)
N1	Zn1	Cl2	102.40(10)	C17	C13	C14	108.8(4)
O1	Zn1	Cl1	110.90(10)	C13	C14	Ru1	71.8(2)
O1	Zn1	Cl2	114.99(10)	C13	C14	C15	107.1(4)
O1	Zn1	N1	106.32(13)	C15	C14	Ru1	70.2(2)
C1	P1	Ru1	123.44(13)	C14	C15	Ru1	72.5(2)
C2	P1	Ru1	115.35(13)	C16	C15	Ru1	71.4(2)
C2	P1	C1	97.14(18)	C16	C15	C14	108.7(4)
C3	P1	Ru1	120.50(14)	C15	C16	Ru1	71.8(2)
C3	P1	C1	97.06(18)	C15	C16	C17	108.1(4)
C3	P1	C2	98.11(19)	C17	C16	Ru1	72.4(2)
C7	P2	Ru1	114.92(13)	C13	C17	Ru1	72.0(2)
C7	P2	C8	96.60(19)	C13	C17	C16	107.3(4)
C8	P2	Ru1	124.83(14)	C16	C17	Ru1	70.0(2)
C9	P2	Ru1	118.80(13)				

Table S14. Bond Angles for **18**.

Atom	Atom	Atom	Angle/°	Atom	Atom	Atom	Angle/°
P1	Ru1	P2	95.44(3)	C10	C11	Ru1	71.7(2)
P1	Ru1	P3	94.27(3)	C10	C11	C12	108.0(3)
P2	Ru1	P3	105.94(3)	C12	C11	Ru1	70.90(19)
C8	Ru1	P1	97.18(9)	C8	C12	Ru1	70.97(18)
C8	Ru1	P2	150.85(10)	C8	C12	C11	108.2(3)
C8	Ru1	P3	99.20(9)	C11	C12	Ru1	72.10(19)
C8	Ru1	C9	37.21(12)	C14	C13	P2	120.9(3)
C8	Ru1	C10	62.02(13)	C14	C13	C18	118.3(3)
C8	Ru1	C11	61.98(13)	C18	C13	P2	120.0(3)
C8	Ru1	C12	37.14(12)	C15	C14	C13	120.5(3)
C9	Ru1	P1	132.52(9)	C14	C15	C16	120.6(3)
C9	Ru1	P2	130.58(10)	C17	C16	C15	119.7(3)
C9	Ru1	P3	84.62(9)	C16	C17	C18	119.9(3)
C9	Ru1	C10	36.79(13)	C17	C18	C13	121.0(3)
C9	Ru1	C11	61.46(13)	C20	C19	P2	122.2(3)
C9	Ru1	C12	61.69(12)	C20	C19	C24	118.1(3)
C10	Ru1	P1	151.45(9)	C24	C19	P2	119.7(3)
C10	Ru1	P2	95.85(9)	C21	C20	C19	121.4(3)
C10	Ru1	P3	107.63(9)	C20	C21	C22	120.0(3)
C11	Ru1	P1	117.51(9)	C23	C22	C21	119.1(3)
C11	Ru1	P2	88.89(9)	C24	C23	C22	120.7(3)
C11	Ru1	P3	143.75(9)	C23	C24	C19	120.7(3)
C11	Ru1	C10	36.82(13)	C26	C25	P2	117.1(3)
C12	Ru1	P1	89.83(9)	C26	C25	C30	118.6(3)
C12	Ru1	P2	117.05(9)	C30	C25	P2	123.8(3)
C12	Ru1	P3	136.21(9)	C27	C26	C25	121.2(3)
C12	Ru1	C10	61.71(12)	C26	C27	C28	119.5(4)
C12	Ru1	C11	36.99(12)	C29	C28	C27	120.1(3)
C3	P1	Ru1	122.91(12)	C28	C29	C30	120.0(3)
C3	P1	C1	95.87(15)	C29	C30	C25	120.5(4)
C1	P1	Ru1	123.97(11)	C32	C31	P3	125.5(3)
C2	P1	Ru1	111.79(11)	C32	C31	C36	118.5(3)
C2	P1	C3	94.83(16)	C36	C31	P3	115.9(3)
C2	P1	C1	102.13(16)	C31	C32	C33	120.0(3)
C13	P2	Ru1	106.31(11)	C34	C33	C32	120.6(4)
C13	P2	C19	101.66(16)	C35	C34	C33	119.7(3)
C13	P2	C25	104.21(16)	C34	C35	C36	119.9(3)
C19	P2	Ru1	116.52(11)	C35	C36	C31	121.2(3)
C25	P2	Ru1	127.90(12)	C38	C37	P3	122.4(3)
C25	P2	C19	96.97(15)	C38	C37	C42	118.6(3)
C31	P3	Ru1	125.15(11)	C42	C37	P3	119.0(3)
C31	P3	C37	98.04(15)	C37	C38	C39	120.6(3)
C31	P3	C43	104.51(16)	C40	C39	C38	120.2(3)
C37	P3	Ru1	118.54(12)	C39	C40	C41	119.9(3)
C43	P3	Ru1	107.03(11)	C40	C41	C42	120.0(3)
C43	P3	C37	100.28(15)	C41	C42	C37	120.6(3)
C1	N1	C6	110.4(3)	C44	C43	P3	120.6(3)
C4	N1	C1	112.6(3)	C48	C43	P3	120.9(3)
C4	N1	C6	110.0(3)	C48	C43	C44	118.2(3)
C2	N2	C5	110.9(3)	C45	C44	C43	120.4(3)
C2	N2	C7	109.7(3)	C46	C45	C44	120.4(3)
C7	N2	C5	109.6(3)	C47	C46	C45	119.8(3)
C3	N3	C4	114.2(3)	C46	C47	C48	120.0(3)

C5	N3	C3	113.5(3)	C47	C48	C43	121.2(3)
C5	N3	C4	115.0(3)	O1TF	S1TF	O2TF	114.14(18)
N3	C3	P1	109.2(2)	O1TF	S1TF	C1TF	103.04(19)
N1	C1	P1	110.8(2)	O2TF	S1TF	C1TF	102.92(19)
N2	C2	P1	112.4(2)	O3TF	S1TF	O1TF	115.24(18)
N1	C4	N3	112.2(3)	O3TF	S1TF	O2TF	115.55(19)
N3	C5	N2	113.7(3)	O3TF	S1TF	C1TF	103.51(19)
C9	C8	Ru1	71.79(18)	F1TF	C1TF	S1TF	111.3(3)
C12	C8	Ru1	71.89(18)	F2TF	C1TF	S1TF	112.4(3)
C12	C8	C9	107.4(3)	F2TF	C1TF	F1TF	107.8(3)
C8	C9	Ru1	70.99(18)	F2TF	C1TF	F3TF	107.1(4)
C10	C9	Ru1	72.37(19)	F3TF	C1TF	S1TF	111.4(3)
C10	C9	C8	108.7(3)	F3TF	C1TF	F1TF	106.5(3)
C9	C10	Ru1	70.83(19)	Cl1H	C1H	Cl2H	109.3(2)
C9	C10	C11	107.6(3)	Cl1H	C1H	Cl3H	111.7(2)
C11	C10	Ru1	71.5(2)	Cl3H	C1H	Cl2H	110.0(2)

Table S15. Bond Angles for **21**.

Atom	Atom	Atom	Angle/°	Atom	Atom	Atom	Angle/°
P1	Ru1	P2	95.65(5)	C25	C26	P3	120.8(4)
P1	Ru1	P3	97.09(5)	C28	C27	P3	122.9(4)
P2	Ru1	P3	97.14(5)	C32	C27	P3	117.8(4)
C33	Ru1	P1	151.67(15)	C32	C27	C28	119.0(5)
C33	Ru1	P2	101.77(15)	C29	C28	C27	119.7(5)
C33	Ru1	P3	102.64(15)	C30	C29	C28	120.6(5)
C33	Ru1	C35	61.6(2)	C29	C30	C31	120.3(6)
C33	Ru1	C37	37.1(2)	C30	C31	C32	119.6(6)
C34	Ru1	P1	125.19(15)	C31	C32	C27	120.8(5)
C34	Ru1	P2	137.83(15)	C34	C33	Ru1	71.4(3)
C34	Ru1	P3	88.55(14)	C34	C33	C37	107.9(5)
C34	Ru1	C33	36.8(2)	C37	C33	Ru1	71.6(3)
C34	Ru1	C35	36.76(19)	C33	C34	Ru1	71.8(3)
C34	Ru1	C36	62.2(2)	C33	C34	C35	108.8(5)
C34	Ru1	C37	61.7(2)	C35	C34	Ru1	72.1(3)
C35	Ru1	P1	92.50(14)	C34	C35	Ru1	71.1(3)
C35	Ru1	P2	149.60(14)	C34	C35	C36	107.7(5)
C35	Ru1	P3	110.85(14)	C36	C35	Ru1	70.8(3)
C36	Ru1	P1	90.64(17)	C35	C36	Ru1	71.6(3)
C36	Ru1	P2	112.94(15)	C37	C36	Ru1	71.7(3)
C36	Ru1	P3	148.05(15)	C37	C36	C35	107.2(5)
C36	Ru1	C33	62.1(2)	C33	C37	Ru1	71.4(3)
C36	Ru1	C35	37.59(19)	C36	C37	Ru1	71.3(3)
C36	Ru1	C37	36.9(2)	C36	C37	C33	108.4(5)
C37	Ru1	P1	122.24(17)	O1T	S1T	C1T	104.8(3)
C37	Ru1	P2	89.37(15)	O1T	S1T	O3TB	107.5(4)
C37	Ru1	P3	139.35(16)	O1T	S1T	O3TA	98.6(6)
C37	Ru1	C35	61.8(2)	O2TB	S1T	O1T	126.4(5)
C1	P1	Ru1	116.36(19)	O2TB	S1T	C1T	104.6(4)
C2	P1	Ru1	116.15(19)	O2TB	S1T	O3TB	111.1(5)
C2	P1	C1	96.4(3)	O3TB	S1T	C1T	98.5(4)
C3	P1	Ru1	124.07(18)	O2TA	S1T	O1T	127.5(6)
C3	P1	C1	100.5(3)	O2TA	S1T	C1T	115.8(7)

C3	P1	C2	98.5(3)	O2TA	S1T	O3TA	105.7(7)
C8	P2	Ru1	118.41(18)	O3TA	S1T	C1T	99.4(6)
C9	P2	Ru1	115.21(18)	F1T	C1T	S1T	111.2(5)
C9	P2	C8	97.0(3)	F1T	C1T	F2T	106.7(6)
C9	P2	C10	98.5(3)	F1T	C1T	F3T	107.7(5)
C10	P2	Ru1	125.4(2)	F2T	C1T	S1T	111.3(5)
C10	P2	C8	96.9(3)	F2T	C1T	F3T	107.8(6)
C14	P3	Ru1	112.81(16)	F3T	C1T	S1T	111.9(4)
C14	P3	C26	102.1(2)	F4TA	C2T	S2TA	108.0(6)
C26	P3	Ru1	120.31(16)	F4TA	C2T	F6TA	107.6(7)
C27	P3	Ru1	115.07(16)	F5TA	C2T	S2TA	110.4(7)
C27	P3	C14	99.9(2)	F5TA	C2T	F4TA	110.0(8)
C27	P3	C26	104.0(2)	F5TA	C2T	F6TA	113.0(8)
C1	N1	C5	111.5(5)	F6TA	C2T	S2TA	107.5(7)
C1	N1	C7	110.5(5)	F4TB	C2T	S2TB	104.1(7)
C7	N1	C5	109.2(5)	F5TB	C2T	S2TB	106.6(7)
C4	N2	C2	111.9(5)	F5TB	C2T	F4TB	108.0(9)
C5	N2	C2	113.0(5)	F6TB	C2T	S2TB	111.2(8)
C5	N2	C4	114.6(5)	F6TB	C2T	F4TB	111.1(9)
C3	N3	C6	109.1(5)	F6TB	C2T	F5TB	115.2(10)
C4	N3	C3	111.5(5)	O4TA	S2TA	C2T	106.5(10)
C4	N3	C6	113.8(5)	O4TA	S2TA	O5TA	116.8(12)
C11	N4	C8	111.5(5)	O5TA	S2TA	C2T	104.6(8)
C12	N4	C8	112.9(5)	O6TA	S2TA	C2T	105.8(7)
C12	N4	C11	110.3(5)	O6TA	S2TA	O4TA	110.0(12)
C11	N5	C10	111.9(5)	O6TA	S2TA	O5TA	112.2(12)
C13	N5	C10	112.9(5)	O4TB	S2TB	C2T	102.9(7)
C13	N5	C11	110.9(5)	O5TB	S2TB	C2T	102.6(6)
C9	N6	C12	110.9(5)	O5TB	S2TB	O4TB	113.3(9)
C9	N6	C13	111.1(5)	O6TB	S2TB	C2T	106.0(6)
C12	N6	C13	108.1(5)	O6TB	S2TB	O4TB	115.6(9)
N1	C1	P1	114.4(4)	O6TB	S2TB	O5TB	114.5(10)
N2	C2	P1	110.5(4)	O7TA	S3TB	C3TA	103.6(5)
N3	C3	P1	113.5(4)	O8TA	S3TB	O7TA	113.5(5)
N2	C4	N3	112.3(5)	O8TA	S3TB	C3TA	105.0(5)
N2	C5	N1	113.1(5)	O9TA	S3TB	O7TA	114.6(5)
N4	C8	P2	112.4(4)	O9TA	S3TB	O8TA	115.3(5)
N6	C9	P2	112.0(4)	O9TA	S3TB	C3TA	102.9(5)
N5	C10	P2	112.2(4)	F7TA	C3TA	S3TB	112.4(7)
N5	C11	N4	112.4(5)	F7TA	C3TA	F9TA	106.4(8)
N4	C12	N6	111.3(5)	F8TA	C3TA	S3TB	112.1(7)
N5	C13	N6	111.3(5)	F8TA	C3TA	F7TA	109.6(8)
C15	C14	P3	121.8(4)	F8TA	C3TA	F9TA	105.6(8)
C15	C14	C19	118.5(5)	F9TA	C3TA	S3TB	110.3(7)
C19	C14	P3	119.7(4)	O7TB	S3TA	O9TB	108.6(10)
C16	C15	C14	120.2(6)	O7TB	S3TA	C3TB	102.2(10)
C17	C16	C15	121.1(6)	O8TB	S3TA	O7TB	116.5(12)
C16	C17	C18	119.7(5)	O8TB	S3TA	O9TB	119.2(11)
C17	C18	C19	120.3(6)	O8TB	S3TA	C3TB	107.4(10)
C18	C19	C14	120.2(5)	O9TB	S3TA	C3TB	100.1(9)
C22	C21	C26	120.3(6)	F7TB	C3TB	S3TA	114.1(13)
C23	C22	C21	121.1(6)	F7TB	C3TB	F9TB	105.7(14)
C22	C23	C24	119.1(5)	F8TB	C3TB	S3TA	111.8(12)
C25	C24	C23	120.7(6)	F8TB	C3TB	F7TB	108.5(14)
C24	C25	C26	120.6(5)	F8TB	C3TB	F9TB	108.4(14)
C21	C26	P3	120.8(4)	F9TB	C3TB	S3TA	108.1(12)
C21	C26	C25	118.2(5)				

Table S16. Bond Angles for **22**.

Atom	Atom	Atom	Angle/°	Atom	Atom	Atom	Angle/°
P1	Ru1	P3	95.95(3)	N6	C8	P2	113.4(2)
P2	Ru1	P1	94.12(3)	N4	C9	P2	113.4(2)
P2	Ru1	P3	98.64(3)	N5	C10	P2	112.3(2)
C32	Ru1	P1	93.70(9)	N4	C11	N5	114.7(3)
C32	Ru1	P2	111.32(9)	N6	C12	N4	114.3(3)
C32	Ru1	P3	147.71(9)	N5	C13	N6	114.3(3)
C33	Ru1	P1	95.45(9)	C15	C14	P3	117.7(2)
C33	Ru1	P2	147.81(9)	C19	C14	P3	123.6(3)
C33	Ru1	P3	110.80(10)	C19	C14	C15	118.7(3)
C33	Ru1	C32	37.43(12)	C16	C15	C14	121.0(3)
C33	Ru1	C36	61.79(12)	C15	C16	C17	120.3(3)
C34	Ru1	P1	128.06(10)	C16	C17	C18	119.2(3)
C34	Ru1	P2	136.35(10)	C17	C18	C19	120.6(3)
C34	Ru1	P3	88.66(9)	C14	C19	C18	120.2(3)
C34	Ru1	C32	61.50(12)	C21	C20	P3	120.6(2)
C34	Ru1	C33	36.76(13)	C25	C20	P3	121.3(2)
C34	Ru1	C35	37.01(13)	C25	C20	C21	118.1(3)
C34	Ru1	C36	61.50(13)	C22	C21	C20	120.9(3)
C35	Ru1	P1	154.65(9)	C23	C22	C21	120.5(3)
C35	Ru1	P2	99.88(9)	C22	C23	C24	119.2(3)
C35	Ru1	P3	102.64(9)	C25	C24	C23	120.6(3)
C35	Ru1	C32	61.55(12)	C24	C25	C20	120.7(3)
C35	Ru1	C33	61.96(12)	C27	C26	P3	123.7(3)
C35	Ru1	C36	37.07(12)	C31	C26	P3	117.3(2)
C36	Ru1	P1	123.78(9)	C31	C26	C27	118.8(3)
C36	Ru1	P2	87.49(9)	C28	C27	C26	120.1(3)
C36	Ru1	P3	139.38(9)	C29	C28	C27	120.7(3)
C36	Ru1	C32	36.39(12)	C28	C29	C30	119.6(3)
C1	P1	Ru1	124.36(11)	C31	C30	C29	119.8(3)
C2	P1	Ru1	112.19(11)	C30	C31	C26	120.9(3)
C2	P1	C1	100.09(15)	C33	C32	Ru1	71.12(18)
C2	P1	C3	96.60(15)	C36	C32	Ru1	71.78(18)
C3	P1	Ru1	121.81(11)	C36	C32	C33	108.3(3)
C3	P1	C1	96.57(15)	C32	C33	Ru1	71.44(18)
C8	P2	Ru1	115.33(11)	C34	C33	Ru1	71.01(18)
C8	P2	C9	96.65(16)	C34	C33	C32	106.8(3)
C8	P2	C10	98.68(16)	C33	C34	Ru1	72.22(19)
C9	P2	Ru1	116.55(12)	C33	C34	C35	109.3(3)
C10	P2	Ru1	127.88(11)	C35	C34	Ru1	71.78(18)
C10	P2	C9	95.87(17)	C34	C35	Ru1	71.21(19)
C14	P3	Ru1	110.63(11)	C34	C35	C36	107.3(3)
C14	P3	C20	101.28(15)	C36	C35	Ru1	71.91(19)
C14	P3	C26	102.28(14)	C32	C36	Ru1	71.83(18)
C20	P3	Ru1	117.42(10)	C32	C36	C35	108.4(3)
C26	P3	Ru1	122.51(11)	C35	C36	Ru1	71.02(19)
C26	P3	C20	99.75(14)	O1TA	S1TA	O2TA	113.73(18)
C1	N1	C4	113.6(3)	O1TA	S1TA	O3TA	115.59(18)
C1	N1	C6	110.4(3)	O1TA	S1TA	C1TA	103.1(2)
C4	N1	C6	109.8(3)	O2TA	S1TA	C1TA	103.4(2)
C5	N2	C2	110.1(3)	O3TA	S1TA	O2TA	115.18(18)
C5	N2	C7	110.5(3)	O3TA	S1TA	C1TA	103.5(2)
C7	N2	C2	109.2(3)	F1TA	C1TA	S1TA	111.8(4)
C3	N3	C4	113.9(3)	F1TA	C1TA	F2TA	107.2(4)

C5	N3	C3	112.3(3)	F1TA	C1TA	F3TA	109.1(4)
C5	N3	C4	114.5(3)	F2TA	C1TA	S1TA	111.7(3)
C11	N4	C9	110.5(3)	F2TA	C1TA	F3TA	106.5(4)
C11	N4	C12	108.4(3)	F3TA	C1TA	S1TA	110.3(3)
C12	N4	C9	111.2(3)	O3TB	S1TB	O1TB	120(2)
C11	N5	C10	111.0(3)	O3TB	S1TB	O2TB	122(2)
C11	N5	C13	108.1(3)	O3TB	S1TB	C1TB	99.0(13)
C13	N5	C10	111.9(3)	O1TB	S1TB	O2TB	113(2)
C8	N6	C13	110.5(3)	O1TB	S1TB	C1TB	98.3(13)
C12	N6	C8	110.3(3)	O2TB	S1TB	C1TB	95.3(13)
C12	N6	C13	109.5(3)	F1TB	C1TB	S1TB	120(3)
N1	C1	P1	111.3(2)	F2TB	C1TB	F1TB	101(4)
N2	C2	P1	113.3(2)	F2TB	C1TB	S1TB	107.0(17)
N3	C3	P1	109.5(2)	F3TB	C1TB	F1TB	110(4)
N1	C4	N3	113.2(3)	F3TB	C1TB	S1TB	108.4(18)
N3	C5	N2	114.0(3)	F3TB	C1TB	F2TB	110(3)

References

(1) Daigle, D. J. Volume 32. In *Inorganic Chemistry*; 1998; Vol. 32, pp 40–45. https://doi.org/10.1016/0020-0255(84)90055-0.

(2) Daigle, D. J.; Pepperman, A. B.; Boudreaux, G. Phosphaadamantanes. Synthesis of 2-thia-1,3,5-triaza-7-phosphaadamantane 2,2-dioxide and Derivatives. *Journal of Heterocyclic Chemistry*. 1974. https://doi.org/10.1002/jhet.5570110644.

(3) Daigle, D. J.; Pepperman, A. B.; Vail, S. L. Synthesis of a Monophosphorus Analog of Hexamethylenetetramine. *Journal of Heterocyclic Chemistry*. 1974, pp 407–408. https://doi.org/10.1002/jhet.5570110326.

(4) Fisher, K. J.; Alyea, E. C.; Shahnazarian, N. A 31P Nmr Study of the Water Soluble Derivatives of l, 3, 5-Triaza-7-Phosphaadamantane (Pta). *Phosphorus, Sulfur, and Silicon and the Related Elements* **1990**, *48* (1–4), 37–40. https://doi.org/10.1080/10426509008045879.

(5) Mena-Cruz, A.; Lorenzo-Luis, P.; Romerosa, A.; Saoud, M.; Serrano-Ruiz, M. Synthesis of the Water Soluble Ligands DmPTA and DmoPTA and the Complex [RuClCp(HdmoPTA)(PPh3)](OSO2CF3) (DmPTA = N,N′-Dimethyl-1,3,5-Triaza-7-Phosphaadamantane, DmoPTA = 3,7-Dimethyl-1,3,7-Triaza-5-Phosphabicyclo[3.3.1]Nonane, HdmoPTA = 3,7-H-3,7-Dimethy. *Inorganic Chemistry* **2007**, *46* (15), 6120–6128. https://doi.org/10.1021/ic070168m.

(6) Darensbourg, D. J.; Ortiz, C. G.; Kamplain, J. W. A New Water-Soluble Phosphine Derived from 1,3,5-Triaza-7-Phosphaadamantane (PTA), 3,7-Diacetyl-1,3,7-Triaza-5-Phosphabicyclo[3.3.1]Nonane. Structural, Bonding, and Solubility Properties. *Organometallics* **2004**, *23* (8), 1747–1754. https://doi.org/10.1021/om0343059.

(7) Akbayeva, D. N.; Gonsalvi, L.; Oberhauser, W.; Peruzzini, M.; Vizza, F.; Brüggeller, P.; Romerosa, A.; Sava, G.; Bergamo, A. Synthesis, Catalytic

Properties and Biological Activity of New Water Soluble Ruthenium Cyclopentadienyl PTA Complexes [(C 5 R 5)RuCl(PTA) 2] (R = H, Me; PTA = 1,3,5-Triaza-7-Phosphaadamantane) †. **2002**. https://doi.org/10.1039/b210102e.

(8) Scalambra, F.; Serrano-Ruiz, M.; Romerosa, A. Water and Catalytic Isomerization of Linear Allylic Alcohols by [RuCp(H2O-KO)(PTA)2]+ (PTA = 1,3,5-Triaza-7-Phosphaadamantane). *Dalton Transactions* **2017**, *46* (18), 5864–5871. https://doi.org/10.1039/c6dt04262g.

(9) Serrano-Ruiz, M.; Lorenzo-Luis, P.; Romerosa, A.; Mena-Cruz, A. Catalytic Isomerization of Allylic Alcohols in Water by [RuClCp(PTA)2], [RuClCp(HPTA)2]Cl2·2H2O, [RuCp(DMSO-KS)(PTA)2]Cl, [RuCp(DMSO-KS)(PTA)2](OSO2CF3) and [RuCp(DMSO-KS)(HPTA)2]Cl3·2H2O. *Journal of the Chemical Society. Dalton Transactions* **2013**, *42* (21), 7622–7630. https://doi.org/10.1039/c3dt32998d.

(10) Serrano-Ruiz, M.; Imberti, S.; Bernasconi, L.; Jadagayeva, N.; Scalambra, F.; Romerosa, A. Study of the Interaction of Water with the Aqua-Soluble Dimeric Complex [RuCp(PTA)2-μ-CN-1KC:2K2 N-RuCp(PTA)2](CF3SO3) (PTA = 1,3,5-Triaza-7-Phosphaadamantane) by Neutron and X-Ray Diffraction in Solution. *Chemical Communications* **2014**, *50* (78), 11587–11590. https://doi.org/10.1039/c4cc05225k.

(11) Scalambra, F.; Serrano-Ruiz, M.; Gudat, D.; Romerosa, A. Amorphization of a Ru-Ru-Cd-Coordination Polymer at Low Pressure. *ChemistrySelect* **2016**, *1* (5), 901–905. https://doi.org/10.1002/slct.201600242.

(12) Romerosa, A.; Campos-Malpartida, T.; Lidrissi, C.; Saoud, M.; Serrano-Ruiz, M.; Peruzzini, M.; Garrido-Cárdenas, J. A.; García-Maroto, F. Synthesis, Characterization, and DNA Binding of New Water-Soluble Cyclopentadienyl Ruthenium(II) Complexes Incorporating Phosphines. *Inorganic Chemistry* **2006**, *45* (3), 1289–1298. https://doi.org/10.1021/ic051053q.

(13) Mendoza, Z.; Lorenzo-Luis, P.; Serrano-Ruiz, M.; Martín-Batista, E.; Padrón, J. M.; Scalambra, F.; Romerosa, A. Synthesis and Antiproliferative Activity of [RuCp(PPh3)2(HdmoPTA)](OSO2CF3)2 (HdmoPTA = 3,7-H-3,7-Dimethyl-1,3,7-Triaza-5-Phosphabicyclo[3.3.1]Nonane). *Inorganic Chemistry* **2016**, *55* (16), 7820–7822. https://doi.org/10.1021/acs.inorgchem.6b01207.

(14) Mendoza, Z.; Lorenzo-Luis, P.; Scalambra, F.; Padrón, J. M.; Romerosa, A. One Step Up in Antiproliferative Activity: The Ru-Zn Complex [RuCp(PPh3)2-μ-DmoPTA-1κP:2κ2N,N′-ZnCl2](CF3SO3). *European Journal of Inorganic Chemistry* **2018**, *2018* (43), 4684–4688. https://doi.org/10.1002/ejic.201800857.

(15) Romerosa, A.; Campos-Malpartida, T.; Lidrissi, C.; Saoud, M.; Serrano-Ruiz, M.; Peruzzini, M.; Garrido-Cárdenas, J. A.; García-Maroto, F. Synthesis, Characterization, and DNA Binding of New Water-Soluble Cyclopentadienyl Ruthenium(II) Complexes Incorporating Phosphines. *Inorganic Chemistry* **2006**, *45* (3), 1289–1298. https://doi.org/10.1021/ic051053q.

(16) Sheldrick, G. M. SHELXT - Integrated Space-Group and Crystal-Structure Determination. *Acta Crystallographica Section A: Foundations of Crystallography* **2015**, *71* (1), 3–8. https://doi.org/10.1107/S2053273314026370.

(17) Sheldrick, G. M. Crystal Structure Refinement with SHELXL. *Acta Crystallographica Section C: Structural Chemistry* **2015**, *71* (Pt 1), 3–8. https://doi.org/10.1107/S2053229614024218.

(18) Dolomanov, O. V.; Bourhis, L. J.; Gildea, R. J.; Howard, J. A. K.; Puschmann, H. OLEX2: A Complete Structure Solution, Refinement and Analysis Program. *Journal of Applied Crystallography* **2009**, *42* (2), 339–341. https://doi.org/10.1107/S0021889808042726.
